# Projections of Global Mortality and Burden of Disease from 2002 to 2030

**DOI:** 10.1371/journal.pmed.0030442

**Published:** 2006-11-28

**Authors:** Colin D Mathers, Dejan Loncar

**Affiliations:** Evidence and Information for Policy Cluster, World Health Organization, Geneva, Switzerland; Johns Hopkins School of Public Health, United States of America

## Abstract

**Background:**

Global and regional projections of mortality and burden of disease by cause for the years 2000, 2010, and 2030 were published by Murray and Lopez in 1996 as part of the Global Burden of Disease project. These projections, which are based on 1990 data, continue to be widely quoted, although they are substantially outdated; in particular, they substantially underestimated the spread of HIV/AIDS. To address the widespread demand for information on likely future trends in global health, and thereby to support international health policy and priority setting, we have prepared new projections of mortality and burden of disease to 2030 starting from World Health Organization estimates of mortality and burden of disease for 2002. This paper describes the methods, assumptions, input data, and results.

**Methods and Findings:**

Relatively simple models were used to project future health trends under three scenarios—baseline, optimistic, and pessimistic—based largely on projections of economic and social development, and using the historically observed relationships of these with cause-specific mortality rates. Data inputs have been updated to take account of the greater availability of death registration data and the latest available projections for HIV/AIDS, income, human capital, tobacco smoking, body mass index, and other inputs. In all three scenarios there is a dramatic shift in the distribution of deaths from younger to older ages and from communicable, maternal, perinatal, and nutritional causes to noncommunicable disease causes. The risk of death for children younger than 5 y is projected to fall by nearly 50% in the baseline scenario between 2002 and 2030. The proportion of deaths due to noncommunicable disease is projected to rise from 59% in 2002 to 69% in 2030. Global HIV/AIDS deaths are projected to rise from 2.8 million in 2002 to 6.5 million in 2030 under the baseline scenario, which assumes coverage with antiretroviral drugs reaches 80% by 2012. Under the optimistic scenario, which also assumes increased prevention activity, HIV/AIDS deaths are projected to drop to 3.7 million in 2030. Total tobacco-attributable deaths are projected to rise from 5.4 million in 2005 to 6.4 million in 2015 and 8.3 million in 2030 under our baseline scenario. Tobacco is projected to kill 50% more people in 2015 than HIV/AIDS, and to be responsible for 10% of all deaths globally. The three leading causes of burden of disease in 2030 are projected to include HIV/AIDS, unipolar depressive disorders, and ischaemic heart disease in the baseline and pessimistic scenarios. Road traffic accidents are the fourth leading cause in the baseline scenario, and the third leading cause ahead of ischaemic heart disease in the optimistic scenario. Under the baseline scenario, HIV/AIDS becomes the leading cause of burden of disease in middle- and low-income countries by 2015.

**Conclusions:**

These projections represent a set of three visions of the future for population health, based on certain explicit assumptions. Despite the wide uncertainty ranges around future projections, they enable us to appreciate better the implications for health and health policy of currently observed trends, and the likely impact of fairly certain future trends, such as the ageing of the population, the continued spread of HIV/AIDS in many regions, and the continuation of the epidemiological transition in developing countries. The results depend strongly on the assumption that future mortality trends in poor countries will have a relationship to economic and social development similar to those that have occurred in the higher-income countries.

## Abstract 2

The presented projections suggest a dramatic shift in the distribution of deaths from younger to older ages and from communicable, maternal, perinatal, and nutritional causes to non-communicable disease causes. HIV/AIDS and tobacco remain major killers and possible targets for intervention.

## Editors' Summary

**Background.:**

For most of human history, little has been known about the main causes of illness in different countries and which diseases kill most people. But public-health officials need to know whether heart disease kills more people than cancer in their country, for example, or whether diabetes causes more disability than mental illness so that they can use their resources wisely. They also have to have some idea about how patterns of illness (morbidity) and death (mortality) are likely to change so that they can plan for the future. In the early 1990s, the World Bank sponsored the 1990 Global Burden of Disease study carried out by researchers at Harvard University and the World Health Organization (WHO). This study provided the first comprehensive, global estimates of death and illness by age, sex, and region. It also provided projections of the global burden of disease and mortality up to 2020 using models that assumed that health trends are related to a set of independent variables. These variables were income per person (as people become richer, they, live longer), average number of years of education (as this “human capital” increases, so does life expectancy), time (to allow for improved knowledge about various diseases), and tobacco use (a major global cause of illness and death).

**Why Was This Study Done?:**

These health projections have been widely used by WHO and governments to help them plan their health policies. However, because they are based on the 1990 estimates of the global burden of disease, the projections now need updating, particularly since they underestimate the spread of HIV/AIDS and the associated increase in death from tuberculosis. In this study, the researchers used similar methods to those used in the 1990 Global Burden of Disease study to prepare new projections of mortality and burden of disease up to 2030 starting from the 2002 WHO global estimates of mortality and burden of disease.

**What Did the Researchers Do and Find?:**

As before, the researchers used projections of socio-economic development to model future patterns of mortality and illness for a baseline scenario, a pessimistic scenario that assumed a slower rate of socio-economic development, and an optimistic scenario that assumed a faster rate of growth. Their analysis predicts that between 2002 and 2030 for all three scenarios life expectancy will increase around the world, fewer children younger than 5 years will die, and the proportion of people dying from non-communicable diseases such as heart disease and cancer will increase. Although deaths from infectious diseases will decrease overall, HIV/AIDS deaths will continue to increase; the exact magnitude of the increase will depend on how many people have access to antiretroviral drugs and the efficacy of prevention programs. But, even given the rise in HIV/AIDS deaths, the new projections predict that more people will die of tobacco-related disease than of HIV/AIDS in 2015. The researchers also predict that by 2030, the three leading causes of illness will be HIV/AIDS, depression, and ischaemic heart disease (problems caused by a poor blood supply to the heart) in the baseline and pessimistic scenarios; in the optimistic scenario, road-traffic accidents will replace heart disease as the third leading cause (there will be more traffic accidents with faster economic growth).

**What Do These Findings Mean?:**

The models used by the researchers provide a wealth of information about possible patterns of global death and illness between 2002 and 2030, but because they include many assumptions, like all models, they can provide only indications of future trends, not absolute figures. For example, based on global mortality data from 2002, the researchers estimate that global deaths in 2030 will be 64.9 million under the optimistic scenario. However, the actual figure may be quite a bit bigger or smaller because accurate baseline counts of deaths were not available for every country in the world. Another limitation of the study is that the models used assume that future increases in prosperity in developing countries will affect their population's health in the same way as similar increases affected health in the past in countries with death registration data (these are mostly developed countries). However, even given these and other limitations, the projections reported in this study provide useful insights into the future health of the world. These can now be used by public-health officials to plan future policy and to monitor the effect of new public-health initiatives on the global burden of disease and death.

**Additional Information.:**

Please access these Web sites via the online version of this summary at http://dx.doi.org/10.1371/journal.pmed.0030442.

- World Health Organization, provides information on the Global Burden of Disease Project and links to other related resources Global Burden of Disease Project
- Harvard School of Public Health, Burden of Disease Unit, offers information on the 1990 Global Burden of Disease study and its projections Harvard School of Public Health

## Introduction

As part of the groundbreaking Global Burden of Disease (GBD) study for 1990, Murray and Lopez [1,2] prepared projections of mortality and burden of disease by cause to 2000, 2010, and 2020 under three alternative scenarios. These projections have been widely used and continue to be widely quoted to provide information on likely future trends in global health, for example in the Atlas of Heart Disease and Stroke [3]. However, these projections were based on the GBD 1990 estimates and on projections of HIV/AIDS, smoking, income, and human capital from 1990 to 2020, and are now outdated. The HIV/AIDS projections in particular substantially underestimate the spread of the HIV epidemic and the level of HIV/AIDS mortality around 2000.

We have thus prepared updated projections of mortality and burden of disease from 2002 to 2030 using methods similar to those of the original GBD study and new input information. An earlier version of these projections has already been extensively used in the recently released World Health Organization (WHO) global report “Preventing chronic diseases: A vital investment” [4].

There is a substantial literature on the projection or forecasting of all-cause mortality rates and mortality rates for specific diseases. The methods used fall into two broad groups. First are those methods based on time-series analysis of historical trends in mortality rates. These “aggregate models,” whether for all-cause mortality or for specific causes, use the previous trend of the variable of interest as the basis for predicting its future value. By their data requirements, such methods are generally limited to high-income countries with good death registration data (see, for example, Lee and Carter [5,6]). Second are the “structural models,” which are based on relationships between mortality and a set of independent variables, and are necessarily projections of those independent variables. To the extent that the structural model identifies the important components—and the relationships among them—of the “system” that determines the variable of interest, they offer the potential for more robust predictions. When the underlying system is complex and sensitive to one or more of its components, a shift in some of the system variables can introduce large changes in the outcome that may be missed by extrapolation (such as the discovery of antibiotics and infectious disease trends or the change in tuberculosis mortality after the HIV epidemic). Aggregate models, in contrast, require considerably less knowledge of the system components and the relationships among them. These models can therefore provide more reliable estimates when such information is not available, especially when the system is not very sensitive to its inputs in time intervals that are in the order of the prediction time.

The GBD projections fall into the structural models class, as do a number of more sophisticated and data-demanding risk factor–based models for specific causes [7–10]. Methods also vary as to whether mortality is projected for all age groups simultaneously or separate models are developed for each age–sex group, and as to whether separate cause-specific projections are carried out. To our knowledge, the original GBD projections represent the only attempt to date to project cause-specific mortality, rather than all-cause mortality, for a complete set of causes at global and regional levels. The GBD projections use separate structural models for each of a small set of major cause groups and cause-compositional models for detailed causes within these groups. The latter are a special case of more general cause-compositional models that relate the proportional distributions of causes of death to the levels of all-cause mortality [11].

Recently, both types of projection model have been extended to allow incorporation of prior knowledge and enforce coherence in the resulting mortality estimates across age groups, causes, and regions. Czado et al. [12] extended the Lee-Carter Poisson log-bilinear model into a Bayesian model, in which the prior data impose smoothness of age and period effects in the projections. Girosi and King [13] developed a forecasting model that models future values of log-mortality as functions of covariates known to affect risk of death. They also used a Bayesian approach to allow different country–age–group cross-sections to “borrow strength” from each other in order to overcome the serious data limitations faced for many countries. Their model also constrains the projections to vary smoothly over time and across age groups in accordance with known age profiles for specific causes of death.

The detailed application of such methods may result in substantially improved forecasts of global mortality and burden of disease trends in the next few years. In the meantime, these updated projections provide a comprehensive update of the original GBD projections, with some methodological improvements and extensions.

## Methods

The methods used in the present study for the projections of mortality and burden of disease are similar to those used in the original GBD study [2]. Separate projections models for males and females and for seven age groups (0–4, 5–14, 15–29, 30–44, 45–59, 60–69, and 70 y and older) were developed to produce parsimonious equations for ten cause-of-death clusters. Baseline, pessimistic, and optimistic projections of covariates in the regression equations were developed and used to project mortality rates for the cause clusters. Separate projection models were developed for HIV/AIDS, tuberculosis, lung cancer, diabetes mellitus, and chronic respiratory diseases. Separate regression equations were developed relating age- and sex-specific mortality rates for 132 detailed causes to the age- and sex-specific mortality rates from corresponding cause clusters, based on International Classification of Diseases Ninth Edition (ICD-9) coded vital registration data from 98 countries.

Whereas the original GBD study carried out projections for eight regions of the world, we carried out these projections at country level, but aggregated the results into regional or income groups for presentation of results. Four income groups were defined based on World Bank estimates of GDP per capita in 2001 (see Table S1 for definitions of the regional and income groups). Baseline estimates at country level for 2002 were derived from the GBD analyses for 2002 as published in the World Health Report 2004 [14]. Data sources and methods for the 2002 estimates were comprehensively documented by Mathers et al. [15], together with an analysis of uncertainty levels of the results [16]. Mortality and burden of disease trends from 1990 to 2001 were recently analyzed by Lopez et al. [17].

Projections of burden of disease measured in disability-adjusted life years (DALYs) were then developed for the three scenarios based on projected mortality rates and alternative assumptions for those causes with little or no mortality. Population projections were developed based on United Nations Population Division projections of fertility and net migration and our projected mortality rates. We describe the main features of the methods, assumptions, and input data in this section, with a particular emphasis on revisions and changes to the original methods used by Murray and Lopez. Protocol S1 and Tables S1–S7 provide full details.

### Regressions for Major-Cause Clusters

Disease and injury causes of death are classified in the GBD using a tree structure in which the first level comprises three broad cause groups: Group I (communicable, maternal, perinatal, and nutritional conditions), Group II (noncommunicable diseases), and Group III (injuries) [15]. We projected age- and sex-specific death rates at country level for ten major-cause clusters: Group I conditions excluding HIV/AIDS (communicable, maternal, perinatal, and nutritional conditions as defined in Table S2), malignant neoplasms (excluding lung cancer), diabetes mellitus, cardiovascular diseases, digestive disorders, chronic respiratory conditions, other Group II diseases, road traffic accidents, other unintentional injuries, and intentional injuries. In contrast to the original GBD projections, diabetes mellitus was treated as a separate cause category from other Group II diseases, since the available evidence suggests that its dominant risk factor, overweight and obesity, is becoming more prevalent over time in both developed and developing regions and is projected to continue to rise [18].

Rather than attempt to model the effects of the many separate direct determinants or risk factors for disease from the limited data that are available, the GBD methodology considered a limited number of socioeconomic variables: (1) average income per capita, measured as gross domestic product (GDP) per capita; (2) the average number of years of schooling in adults, referred to as “human capital”; and (3) time, a proxy measure for the impact of technological change on health status. This latter variable captures the effects of accumulating knowledge and technological development, allowing the implementation of more cost-effective health interventions, both preventive and curative, at constant levels of income and human capital [1].

These socioeconomic variables show clear historical relationships with mortality rates, and may be regarded as indirect, or distal, determinants of health, although it is not necessary for the purposes of projection to know whether these associations are causal. In addition, a fourth variable, tobacco use, was included in the projections for cancers, cardiovascular diseases, and chronic respiratory diseases, because of its overwhelming importance in determining trends for these causes. Tobacco use was measured in terms of “smoking impact”—that component of observed lung cancer mortality attributable to tobacco smoking [19]. This indirect measure of the accumulated hazards provides a better measure than do current smoking rates for the overall health impact of tobacco, taking into account lag times as well as important aspects of exposure such as duration, type, amount, and mode of smoking (see Protocol S1 for more details).

We used a regression equation of the same form as in the original GBD projections:

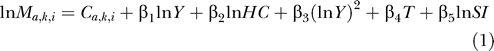
where *C_a,k,i_* is a constant term, *M_a,k,i_* is the mortality level for age group *a,* sex *k,* and cause *i,* and *Y, HC,* and *T* denote GDP per capita, human capital, and time, respectively. The log of the smoking impact *SI* is included in the equation only for malignant neoplasms, cardiovascular diseases, and respiratory diseases. This basic functional relationship makes no specific assumptions about the relationships between these more distal socioeconomic factors and more proximate determinants of mortality rates such as environment, lifestyle, and physiological risk factors. Nevertheless, our regression results indicate that a considerable proportion of the variance in age-, sex-, and cause-specific mortality rates can be explained by this limited set of distal determinants.

Murray and Lopez used a variety of econometric approaches to estimate the equations (Equation 1), including methods that take into account auto-correlation and heteroscedasticity. Because these methods substantially restricted the subset of panel data that could be used, and in particular resulted in much loss of information for moderate- to high-mortality populations, they chose to use ordinary least-squares regression based on the entire dataset for the final set of parameter estimates. We followed the same approach and estimated equations (of the form illustrated by Equation 1) separately for each age–sex–cause group.

Death registration data from 106 countries for 1950–2002 [20] were used to estimate the regression equations. In total, 2,605 country-years were available, almost double those used for the original projections by Murray and Lopez. Although the dataset is extensive, it does not include many observations from populations with high rates of mortality. Whereas the original GBD projections applied a single set of models based on all observed death registration data for projections in all regions, the new projections for low and lower-middle-income countries (income in international dollars less than $3,000 per capita in 2002) were based on the observed relationships for a dataset consisting of 1,734 country-years of observation where income per capita was less than $10,000 per year. Additionally, observed regional trends in child mortality from 1990 to 2002 were compared with those predicted by the projection model for low-income countries conditional on the observed time series for the model covariates for that period. As a result, the regression coefficient for time was set to zero for sub-Saharan Africa, and to 25% of its original value for other low-income countries.

Final parsimonious regression results are listed in Tables S3 and S4. The low-income regression results give somewhat more conservative declines for Group II (noncommunicable) diseases in low-income regions. In general, the final regression equations are broadly similar to those estimated for the original projections. Apart from injuries, they explain a surprising proportion of the variance for many age–sex–cause categories. For males and females aged 70 y and above, the *R* ^2^ for many cause clusters was generally lower than for other age groups, probably reflecting poorer quality of coding of causes of death or the smaller range of variation in mortality rates between countries at these older ages, as well as the inherent reductions in the explanatory power of covariates with increasing death rates with age. The lower proportions of variance explained for injuries may reflect lower levels of temporal variation in these causes as well as more heterogeneous injury causes that may be less well-correlated with income and human capital.

For cause clusters in age–sex groups in which the *R* ^2^ was less than 10%, we carefully examined the consistency of the projections with other age–sex groups, and made a choice between using the final parsimonious regression equation, an alternative assumption such as stable (constant) rates, or a separate prediction model based on projections for the major relevant risk factor (diabetes mellitus and chronic respiratory diseases).

Model predictions of age-, sex-, and cause-specific mortality rates in 2002 for each of the ten clusters of causes were compared for each country with the results of the GBD study for that year. A series of scalars were then derived so that projected values for 2002 were identical to the 2002 GBD results. It was then assumed that these scalars would remain constant over the period 2002–2030.

### Diabetes and Chronic Respiratory Conditions

Initial regression analysis for diabetes mellitus found inconsistent trends between males and females, probably reflecting the large variations across countries and inaccuracies in recording diabetes as the underlying cause of death in many death registration systems. While overall trends for diabetes mortality showed no consistent relationship across the sexes or levels of development, the regression analysis found significant betas for *T* (year) with death rates increasing with time. Since a substantial proportion of diabetes mortality is attributable to overweight and obesity [21], a separate projection model for diabetes mortality was developed using WHO projection of trends in body mass index distributions from 2000 to 2010 (see Protocol S1 for more details).

Initial projections for chronic respiratory diseases resulted in substantially increasing rates for high-income countries, although smoking is the main risk factor and smoking impact has been generally decreasing. It is likely that the initial projections may reflect increasing propensity to code chronic obstructive pulmonary disease (COPD) as the underlying cause of death with time. Projections of mortality for chronic respiratory diseases were thus adjusted for projected changes in smoking impact (see Protocol S1 for more details).

### Projections of Income, Human Capital, and Smoking Impact

Revised country-level projections for income per capita, human capital, and smoking impact were developed for the baseline, pessimistic, and optimistic projections. Income per capita was measured using average GDP per capita expressed in international dollars (purchasing power parity adjusted). Country-specific and regional income growth forecasts by the World Bank were used to project GDP per capita for all WHO member states. Beyond 2015, projected growth rates for most regions approach 3% per annum under the baseline scenario, with somewhat lower growth rates in sub-Saharan Africa, the Middle East, and high-income countries (see Protocol S1 for more details).

Revised estimates and projections of human capital for WHO member states were prepared for the period 1950–2030 drawing on previous estimates by Barro and Lee for 98 countries [22] and observed relationships between growth in human capital and growth in GDP per capita.

Smoking impact was calculated for the historical mortality country–year observations by subtracting nonsmoker lung cancer rates from observed total lung cancer mortality rates in the data. Higher nonsmoker lung cancer mortality rates were used in China and Southeast Asian countries, to reflect the higher levels of lung cancer in nonsmokers due to indoor air pollution from exposure to smoke from solid fuels [23,24].

Ezzati and Lopez developed projections of smoking intensity for 14 sub-regions of the WHO regions for the Comparative Risk Assessment (CRA) project, based on an analysis of past and current age–sex-specific smoking prevalence calibrated to regional characteristics of the tobacco epidemic for different sub-regions [23,24]. These regional projections were used to develop country-specific projections drawing also on more recent World Bank data on trends in adult per capita consumption of cigarettes and recent observed trends in age–sex-specific lung cancer mortality (see Protocol S1 for more details). For four high-income countries, recent projections were used based on an age–period–cohort model estimated using historical data on age–sex-specific tobacco consumption, average tar content, and adult tobacco consumption per capita [25].

### Regression Equations for Detailed Causes

To project death rates for specific causes within the major-cause clusters, we followed the original GBD methodology in estimating the relationship between trends in age–sex-specific mortality for each specific cause with the trend in the age–sex-specific mortality for the major-cause cluster to which the specific cause belongs. To avoid biasing the results due to changes in specific cause death rates associated with use of different versions of ICD, we restricted the analysis to country-years for which deaths were coded using ICD-9. The regression parameters were estimated on 1,357 country-year observations for 98 countries.

For each individual regression, observations were excluded from analysis if they related to fewer than 50 deaths. Regression results were used only where the relationship was reasonably strong, as measured by an *R* ^2^ greater than 0.25 and a *p*-value less than 0.001 for the regression coefficient. The resulting coefficients are shown in Table S5.

The major-cause cluster regression equation for intentional injury predicted generally falling death rates in middle- and low-income countries and rising death rates in high-income countries. For homicide, this is the opposite of the observed trends in the larger high-income countries. For this reason, homicide death rates were assumed to remain constant under the baseline scenario, with some modifications for country-specific observed trends. Similarly, war deaths were assumed to remain constant under the baseline scenario.

For countries with good death registration data and populations of 5 million or more, trends in mortality rates were estimated for ischaemic heart disease, cerebrovascular disease, tuberculosis, suicide, and homicide. These were used to adjust the initial years of relevant country-specific projections to match the recent observed trends.

### Projections for HIV/AIDS and Tuberculosis

We used separate projections for HIV/AIDS mortality under several scenarios derived from existing models. The Joint United Nations Programme on HIV/AIDS (UNAIDS) and WHO [26] have prepared projections of HIV/AIDS mortality under a range of assumptions about the future of the HIV epidemics in all regions and with varying treatment scale-up assumptions for both adult and children using a transmission model previously used to assess the impact of preventive interventions [10,27].

The model includes underlying regional demography, acquisition of HIV and other sexually transmitted infections, and progression from HIV infection to AIDS and death. For countries with generalized epidemics (mostly in sub-Saharan Africa), the model variables were estimated from sentinel site prevalence data. For countries with epidemics concentrated in groups with higher-risk behaviour, the size and HIV prevalence was estimated for each of these groups, and prevalence in low-risk populations was estimated by allowing for transmission from high-risk to low-risk groups via sexual mixing. Projections of these epidemics were based on assumptions about degree of saturation for each of the high-risk groups, time to saturation, and spread from high-risk to low-risk populations over time (see Protocol S1 for further information).

Our baseline projections for HIV/AIDS age–sex-specific mortality rates were based on the UNAIDS and WHO “medium” scenario for treatment scale-up. Under this scenario, antiretroviral therapy (ART) coverage will reach 80% by 2012 in all regions, remaining constant beyond that year. The treatment scenarios assumed no effect of treatment on transmission and incidence rates, and no additional prevention efforts resulting in reduction of transmission and incidence rates.

Our pessimistic projections for HIV/AIDS age–sex-specific mortality rates were based on the UNAIDS and WHO “slow” scenario for treatment scale-up. Under this scenario, ART coverage will reach 60% by 2012 in all regions except Latin America, where it reaches 70% in 2013. HIV/AIDS mortality rates in high-income countries were assumed to remain constant as in the baseline scenario.

Salomon et al. [28] have modelled scenarios for sub-Saharan Africa combining treatment and additional prevention efforts. In one scenario, ART coverage is scaled up and optimal assumptions are made about treatment impact on transmissibility and patient behaviour. A second scenario assumes that an emphasis on treatment leads to less effective implementation of prevention, resulting in only 25% attainment of the maximum potential impact of prevention efforts. For our optimistic projection of HIV/AIDS mortality, we used a third unpublished scenario prepared by Salomon et al. that is approximately halfway between their two published mixed treatment-prevention scenarios. For low- and middle-income countries outside sub-Saharan Africa, we assumed similar proportional reductions in incidence rates for HIV infection due to increased prevention, as estimated for sub-Saharan Africa (see Protocol S1 for more details).

Because of the powerful interaction between tuberculosis and HIV infections in regions such as sub-Saharan Africa, Murray and Lopez modified the original projections from 1990 to 2020 for tuberculosis death rates. As part of the Global Plan to Stop TB covering the period 2006–2015, the Stop TB Partnership has prepared three projection scenarios for tuberculosis incidence, prevalence, and mortality based on different assumptions about the pace of scale-up and coverage of interventions to achieve the UN's Millennium Development Goals for tuberculosis [29]. The “Global Plan” scenario assumes massive scale-up in tuberculosis control activities, achieving case detection levels of more than 70% and using the WHO DOTS (directly observed therapy, short-course) treatment strategy to reach cure rates of more than 85%. The “Sustained DOTS” scenario assumes that case detection and treatment success rates increase until 2005 and then remain constant to 2015. A third “No DOTS” scenario assumes that the DOTS treatment strategy was never introduced in any region, so case detection, treatment, and cure rates would continue as they were pre-DOTS (see Protocol S1 for more details).

The projected annual regional trends in tuberculosis death rates for HIV-negative cases under these three scenarios were used to project tuberculosis death rates for countries in each region as follows. Annual projected trends under the “Sustained DOTS” scenario were used for the baseline projections, and those under the “Global Plan” scenario were used for the optimistic projections. For the pessimistic projections, annual trends in rates were estimated as halfway between those projected under the “sustained DOTS” scenario and those projected under the “No DOTS” scenario from 2006 onwards. For 2016–2030, annual trends from 2015 to 2030 were assumed to converge on the regional projected trends for Group I causes excluding HIV/AIDS under the baseline scenario. For the optimistic and pessimistic scenarios, annual regional trends were assumed to remain constant from 2015 to 2030.

### Projections of Tobacco-Caused Deaths

As part of the WHO's CRA project, Ezzati and Lopez [23] estimated the total mortality attributable to tobacco smoking in 2000 for the world and for WHO sub-regions. Current levels of smoking impact ratio (*SIR*) were used as an indirect indicator of accumulated smoking risk based on excess lung cancer mortality. *SIR* measures the *absolute* excess lung cancer mortality due to smoking in the study population, relative to the *absolute* excess lung cancer mortality in lifelong smokers of the reference population. *SIR* values were calculated from the projected smoking impact variables for individual countries for age groups 30–44 y and older.

Projected mortality attributable to tobacco was estimated using the CRA methods for lung cancer, upper aerodigestive cancer, all other cancers, COPD, other respiratory diseases, cardiovascular diseases, tuberculosis, and selected other disease causes.

### Projecting the Burden of Disease and Injury

The WHO has undertaken new assessments of the GBD for 2000–2002, with consecutive revisions and updates published annually in WHO's World Health Reports. These assessments use a health gap measure, the DALY, developed by Murray and Lopez [30] to quantify the equivalent years of full health lost due to diseases and injury in WHO member states. The data sources and methods used for the GBD 2002 are documented elsewhere [15–17], and summary results for 14 regions of the world are published in the World Health Report 2004 [14] and available at http://www.who.int/evidence/bod.

The burden of disease estimates for 2002 were used as a base for projections of burden of disease to 2030. Years of life lost (YLL) were calculated from the projections of mortality by cause, age, and sex, according to the GBD method. To project DALYs, it is also necessary to project years lived with disability (YLD). Given the lack of good information on trends in disability and health state distributions, the approach used here is an elaboration of the methods and assumptions used by Murray and Lopez in the original GBD projections [1].

YLD projections were generally derived from the YLL projections by applying the ratio of YLD to YLL for 2002. For ischaemic heart disease and stroke, future incidence rates were assumed to decline at 50% of their mortality rate declines reflecting declining case fatality rates as well as incidence rates. For causes where there is little or no mortality, age–sex-specific YLD rates per capita were generally assumed to remain constant into the future. For certain mental disorders, musculoskeletal conditions, and hearing loss, disability weights were assumed to decline somewhat with improvements in income per capita reflecting increasing treatment coverage. YLD rates for nonfatal communicable diseases and nutritional deficiencies were assumed to decline at between 50% and 100% of the mortality rate declines for Group I causes.

### Projections of Population and Numbers of Deaths and DALYs

Our projections of mortality rates, together with UN medium variant assumptions for fertility rate projections and projected migration rates [31], were also used to prepare consistent population projections for all regions. The projected global population in 2015 was 7.1 billion compared to the UN medium variant projection of 7.2 billion, reflecting somewhat higher adult death rates in our mortality projections.

Projected death and DALY rates for 192 WHO member states for 2003–2030 were applied to the projected populations to generate projected numbers of deaths and DALYs for each of the three scenarios. The resulting country projections were added back into regional groups for presentation of results (see Table S1).The choices and assumptions incorporated in each of the baseline, pessimistic, and optimistic scenarios are summarized in Table S7.

## Results

This section provides an overview of the projections. Detailed results for 2002, 2015, and 2030 are presented in Dataset S1 for deaths and Dataset S2 for DALYs. These tables include baseline, pessimistic, and optimistic projected totals for each cause category, as well as a sex and age breakdown for the baseline category.

**Figure 1:**
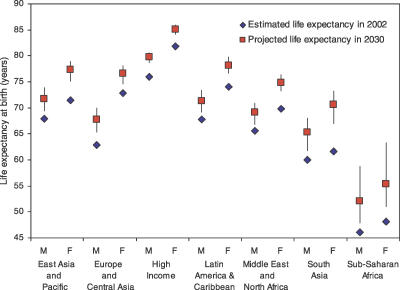
Projected Life Expectancy at Birth in 2030 by World Bank Region and Sex: Baseline, Optimistic, and Pessimistic Scenarios Compared with 2002 Estimates

Figure 1 shows projected life expectancies at birth in 2030 under the three scenarios, for World Bank regions. Life expectancy at birth is projected to increase in all World Bank regions, with the largest increases in the African region and the South Asian regions. However, under the baseline scenario, male life expectancy in the African region will still remain less than 55 y. In all regions except the European region, life expectancy increases are greater for females than for males. Life expectancy for women in the high-income countries may reach 85.0 y by 2030, compared with 79.7 y for men. The highest projected life expectancy in 2030 is for Japanese women at 88.5 y (with a range of 87.7 to 89.2 across the pessimistic and optimistic scenarios). The female–male difference in life expectancy at birth is projected to narrrow from 5.9 y in 2002 to 5.3 y in 2030 in the high-income countries, whereas the gap will more than double in low-income countries to 5.2 y in 2030.

**Figure 2:**
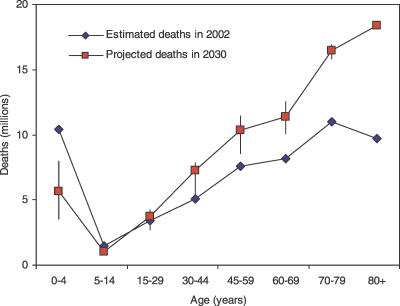
Global Numbers of Deaths by Age and Sex: Baseline, Optimistic, and Pessimistic Scenarios for 2030 Compared with 2002 Estimates

Projected global deaths in 2030 ranged from 64.9 million under the optimistic scenario to 80.7 million under the pessimistic scenario, variations of −11% to +10% relative to the baseline projection of 73.2 million. Figure 2 shows the projected global numbers of deaths in 2030 by age in the three scenarios, compared with the numbers of deaths by age in 2002. In all three scenarios there is a dramatic shift in the distribution of deaths from younger to older ages. The risk of death for children younger than 5 y is projected to fall substantially in the baseline scenario, by almost 25% between 2005 and 2015, and by more than 40% between 2005 and 2030. These rates of decline are similar to those projected between 2000 and 2015 in the original GBD projections. The vertical bars attached to the points for 2030 in Figure 2 represent the range of deaths projected under the optimistic and pessimistic scenarios.

### Trends in Cause-Specific Mortality

Table 1 summarizes the projected annual average changes in age-standardized death rates for selected major causes for the baseline projections for the period 2002–2020. For all the Group I and Group II cause groups in which the projections were based on the major-cause cluster regression equations, age-specific and age-standardized death rates are projected to decline over the next 20 years. The average annual rate of decline is greater (at about 3%) for Group I causes than for Group II causes. The HIV/AIDS projections, discussed in more detail below, have a substantial projected average annual rate of increase of 3% for males and 2% for females. Other causes with projected increases in age-standardized rates include lung cancer, diabetes, chronic respiratory diseases, road traffic accidents, violence, and war.

**Table 1:**
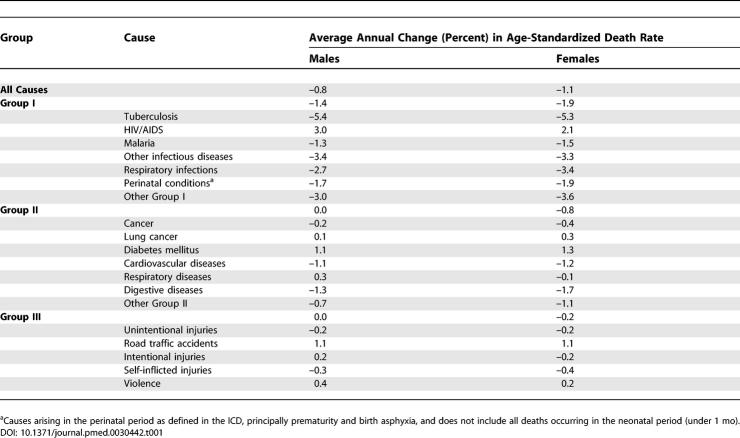
Projected Average Annual Rates of Change in Age-Standardized Death Rates for Selected Causes: World, 2002–2020

The Group I causes, excluding HIV/AIDS and tuberculosis, decline with average annual rates of change typically about one-third slower than the original GBD projections of Murray and Lopez. To some extent, this reflects the more conservative projections for low-income countries, where the coefficient of the time factor was reduced or set to zero. Average rates of decline for Group II causes (excluding lung cancer and chronic respiratory conditions) are similar for females to those in the original projections. However, the differential between males and females in the original projections has disappeared in the current projections, with males having a greater average annual rate of decline for Group II conditions than previously projected.

**Figure 3:**
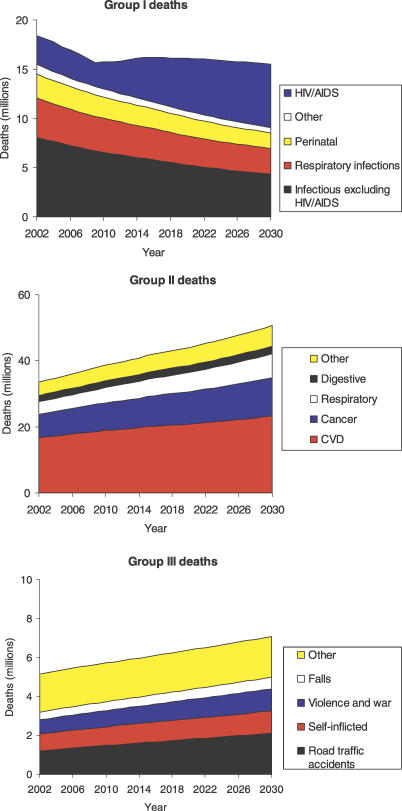
Baseline Projections of Deaths from Group I, Group II, and Group III Causes, World, 2002–2030

Figure 3 summarizes the contributions of major causes to global trends in numbers of deaths for the three major cause groups. Large declines in mortality between 2002 and 2030 are projected for all of the principal Group I causes, with the exception of HIV/AIDS. Under the baseline scenario involving scale-up of ART coverage to 80% by 2012, but not additional prevention efforts, HIV/AIDS deaths increase from 2.8 million in 2002 to 6.5 million in 2030. Total deaths due to other Group I causes decline from 15.5 million in 2002 to 9.0 million in 2030. Unfortunately, this is substantially offset by the projected rise in HIV/AIDS deaths. Under the optimistic scenario involving additional HIV prevention activity, 3.7 million HIV/AIDS deaths are projected for 2030, so that total deaths due to Group I conditions would decline from 32% of all deaths in 2002 to 14% of all deaths in 2030.

Although age-specific death rates for most Group II conditions are projected to decline, ageing of the population will result in significantly increasing total deaths due to most Group II conditions over the next 30 years (Figure 3). Global cancer deaths are projected to increase from 7.1 million in 2002 to 11.5 million in 2030, and global cardiovascular deaths from 16.7 million in 2002 to 23.3 million in 2030. Overall, Group II conditions will account for almost 70% of all deaths in 2030 under the baseline scenario.

The projected 40% increase in global deaths due to injury between 2002 and 2030 are predominantly due to the increasing numbers of road traffic accident deaths, together with increases in population numbers more than offsetting small declines in age-specific death rates for other causes of injury. Road traffic accident deaths are projected to increase from 1.2 million in 2002 to 2.1 million in 2030, primarily due to increased motor vehicle fatalities associated with economic growth in low- and middle-income countries.

### Projections of HIV/AIDS Mortality and Other Selected Causes

**Figure 4:**
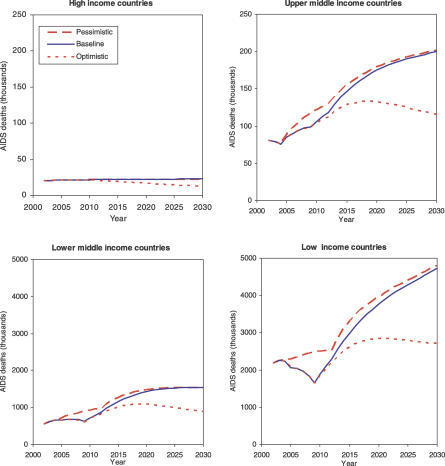
Projections of Total AIDS Deaths (Thousands) by Income Group for Three Scenarios Scenarios are indicated: baseline (solid lines), optimistic (dotted lines), and pessimistic (dashed lines).

Figure 4 summarizes projected HIV/AIDS deaths by income group for the three scenarios. The declining death rates for 2005–2010 in the baseline and pessimistic scenarios, followed by increasing death rates, reflect the effects of the assumed treatment scale-up scenarios. Rapidly increasing levels of ART coverage result in postponement of deaths for a number of years, but once the ART coverage plateaus at its final level, numbers of deaths continue to rise, reflecting largely the underlying growth in population. HIV incidence rates essentially remain constant in the baseline scenario for sub-Saharan Africa, and the global growth in incident cases and in deaths is largely driven by population growth in sub-Saharan Africa.

Under the baseline scenario, the total deaths from HIV/AIDS over the 25-y period 2006–2030 are projected to be 117 million. Under the optimistic scenario, in which additional prevention efforts result in a long-term 3% decline in incidence rates, the projected total deaths over 2006–2030 are 89 million, a saving of 28 million lives.

**Figure 5:**
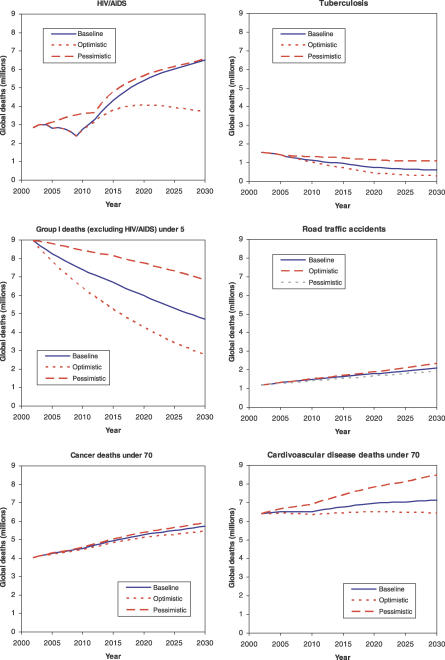
Projections of Global Deaths (Millions) for Selected Causes, for Three Scenarios: Baseline, Optimistic, and Pessimistic, 2002–2030

The ranges defined by the optimistic and pessimistic projections differ substantially by cause (Figure 5). For example, the range for cardiovascular disease before age 70 y is much wider than that for cancers before age 70 y or for road traffic accidents. Group I deaths (excluding HIV/AIDS) have a wider range than most other causes, although the total deaths decline substantially in all three scenarios.

### Comparison with GBD 1990 Projections

**Figure 6:**
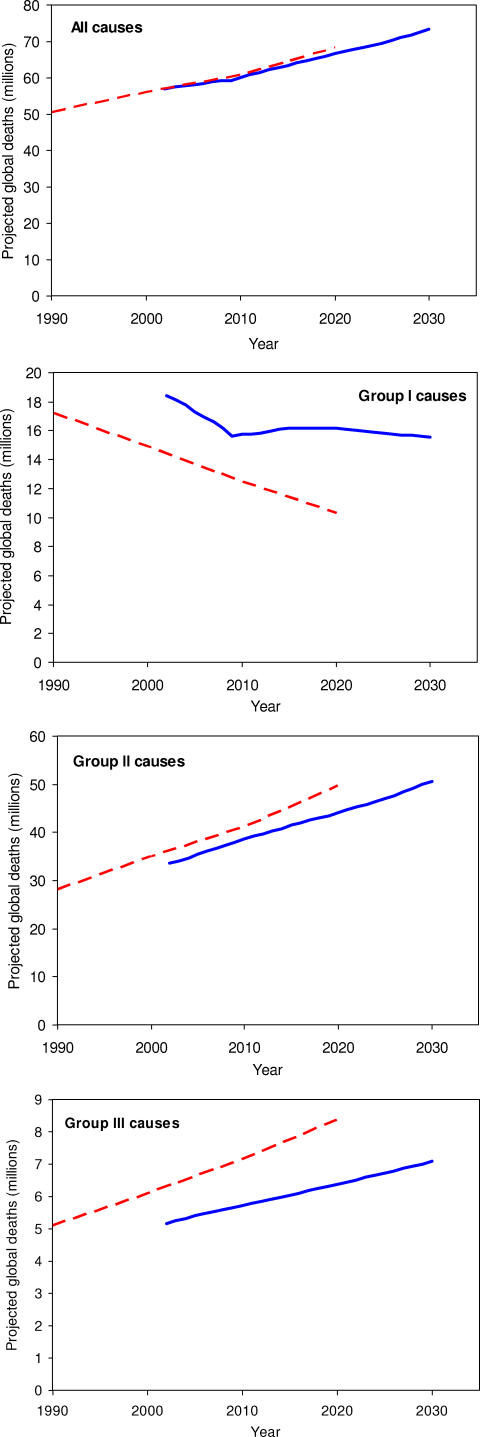
Comparison of Baseline Projections 2002–2030 with the Original GBD Projections 1990–2020: Global Deaths for All Causes, and Major Cause Groups

Figure 6 compares projected global deaths for each of the three major cause groups for 2002–2030 with the corresponding projections for 1990–2020 from the original GBD study [1]. Projections for Group I causes are substantially different, mainly because of the very large difference in projected HIV/AIDS mortality. Total global deaths for Group II and Group III causes are projected to increase at a somewhat slower rate than the original GBD projections, and in addition, the base levels for these causes in 2002 are somewhat lower than the levels projected by the original GBD study.

Despite these differences for all three major cause groups, and differences in projected global population numbers, the projections of total global all-cause deaths are almost identical to those in the original GBD study. Global deaths in 2020 under the baseline scenario are 66.5 million, compared with 68.3 million projected by Murray and Lopez from the 1990 base, and the overall trend is almost identical.

Projected trends for global deaths due to most Group I causes other than HIV/AIDS are broadly similar. The new baseline projections for lung cancer give lower global numbers and a lower rate of increase. Trends in global respiratory deaths are similar for the two sets of projections, but the projected rates of increase in global deaths due to cancers, cardiovascular disease, and digestive disorders are all somewhat slower for the current projections. Increases for road traffic accidents are somewhat slower in the current projections, whereas trends for other unintentional causes are quite similar. There are some substantial differences in trends for intentional causes, reflecting different assumptions and decisions concerning use of the regression parameters.

### Leading Causes of Death

Table 2 lists the 15 leading causes of death according to the baseline scenario for males, females, and both sexes combined as projected for 2030 globally. Table 3 provides similar lists of the ten leading causes of death according to the baseline scenario for the four income groups of countries. The four leading causes of death in all scenarios are projected to be ischaemic heart disease, cerebrovascular disease (stroke), HIV/AIDS, and COPD, although HIV/AIDS moves from third to fourth position in the optimistic scenario.

**Table 2:**
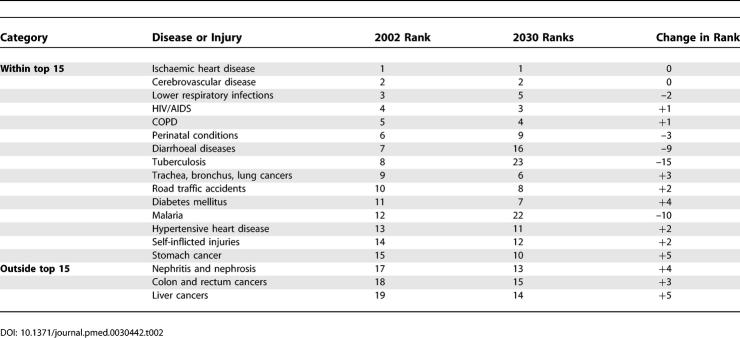
Changes in Rankings for 15 Leading Causes of Death, 2002 and 2030 (Baseline Scenario)

**Table 3:**
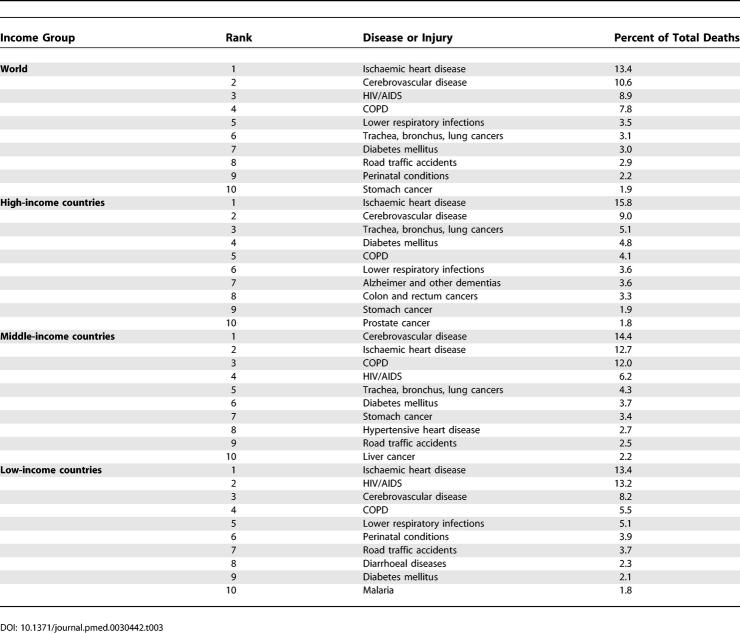
Ten Leading Causes of Death, by Income Group, 2030 (Baseline Scenario)

Table 2 also summarises the changes in rank order of deaths between 2002 and 2030 for the 15 leading causes. Lower-respiratory infections, perinatal conditions, diarrhoeal diseases, malaria, and measles are all projected to decline substantially in importance. On the other hand, diabetes mellitus, lung cancer, stomach cancer, and liver and colorectal cancer are all projected to move up three or more places in the rankings.

### Decomposition

The results discussed so far have described projected changes in mortality in terms of the absolute (and relative) numbers of deaths expected under the various scenarios. These changes may be due to changes in age-specific disease and injury mortality risks, or due to demographic change that alters the size and age composition of the population, or both. Because mortality risks are strongly age dependent for most causes, changes in the age structure of a population may result in substantial changes in the number of deaths, even when the age-specific risks remain unchanged.

**Figure 7:**
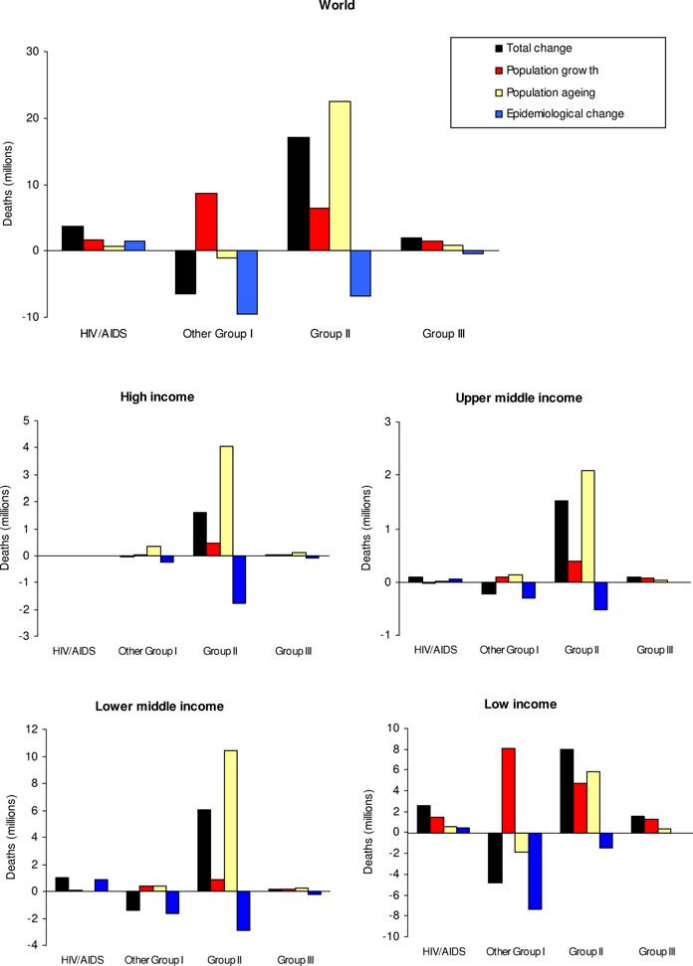
Decomposition of Projected Change in Numbers of Deaths into Demographic and Epidemiological Components, by Broad Cause Group and Income Group, 2002–2030

We further analysed the relative impact of demographic and epidemiological change on the projected numbers of deaths by cause by calculating three hypothetical alternatives. In the first, we calculated the expected number of deaths in 2030 given the 2030 projected age-specific rates under the baseline scenario and the 2002 population. The difference between this and the 2002 mortality estimates is a measure of the change in mortality expected solely on the basis of changing age-specific mortality rates, and is labelled “epidemiological change” in Figure 7.

Second, we calculated the expected number of deaths in 2030 by taking the 2002 age-specific death rates and applying them to the 2030 projected population. The difference between this and the 2002 mortality estimates is a measure of the change in mortality expected solely on the basis of changing demography (including size and age composition of the population). We then repeated this calculation, but applied the 2002 age-specific death rates to projected population numbers for 2030 that matched the total projected male and female population numbers for each country but retained the 2002 age distribution of the population. The difference between this and the 2002 mortality estimates is a measure of the change in mortality expected solely on the basis of population growth excluding changes in age composition. The difference between the total change in mortality due to demography and this latter estimate gives a measure of the effect of the change in age composition of the population alone. These two components of demographic change are labelled “population growth” and “population ageing” in Figure 7. The total projected change in numbers of deaths between 2002 and 2030 is the sum of the population growth, population ageing, and epidemiological change components.

In almost all cases, demographic and epidemiological factors are operating in opposing directions in determining mortality in 2030. The major exception is HIV/AIDS, where demographic and epidemiological change are acting in the same direction to increase total HIV/AIDS deaths to 6.5 million deaths in 2030 under the baseline scenario. Demographic change dominates, as the majority of HIV/AIDS deaths are in sub-Saharan Africa, where population growth is highest and where HIV/AIDS incidence rates are assumed to remain largely constant under the baseline scenario.

For Group I conditions other than HIV/AIDS for which substantial declines in mortality rates are projected, the effect of these declines will be attenuated in most regions by demographic change leading to an increase in the child population most at risk for these conditions. Population growth and population ageing act in opposite directions for Group I mortality excluding HIV/AIDS in low-income countries, but not in other income groups. If future fertility rates are higher than projected, then the higher child population numbers will further offset the projected reductions in death rates for Group I conditions.

For Group II (noncommunicable diseases), demographic changes in all regions will tend to increase deaths substantially, with offsetting reductions in projected death rates in all regions. Population growth and population ageing both act to increase Group II deaths in all regions, although the impact of population ageing is generally much more important than population growth. Population growth has the largest relative impact for low-income countries, and the smallest for lower-middle-income countries. The latter group includes Eastern European populations such as Russia that will experience negative population growth.

For Group III (injuries), demographic change similarly dominates the epidemiological change. The latter is small at group level in most regions, because the projected increase in road traffic fatalities is offset by projected decreases in death rates for other unintentional injuries.

### Tobacco-Attributable Deaths

**Figure 8:**
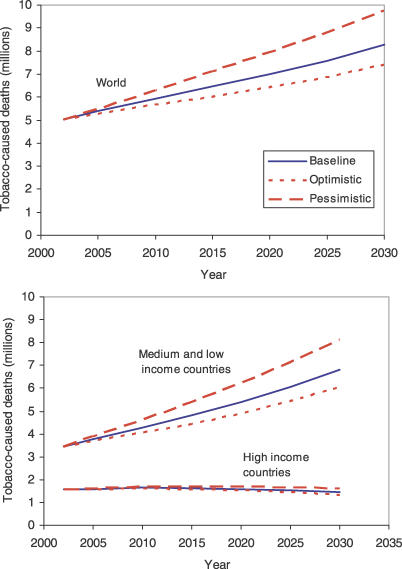
Projected Numbers of Tobacco-Caused Deaths for the World and for High-Income and Middle- plus Low-Income Countries, Three Scenarios, 2002–2030

Figure 8 summarises the projected number of tobacco-attributable deaths for the world, and for high-income and low- and middle-income countries. Under the baseline scenario, total tobacco-attributable deaths will rise from 5.4 million in 2005 to 6.4 million in 2015 and 8.3 million in 2030. Projected deaths for 2030 range from 7.4 million in the optimistic scenario to 9.7 million in the pessimistic scenario. Tobacco-attributable deaths are projected to decline by 9% between 2002 and 2030 in high-income countries, but to double from 3.4 million to 6.8 million in low- and middle-income countries.

Table 4 divides the projected 2015 global mortality due to smoking into leading causes: cancers are responsible for one-third of the deaths, followed by cardiovascular diseases and chronic respiratory diseases, each responsible for 30% of the deaths. According to our baseline projection, smoking will kill 50% more people in 2015 than HIV/AIDS, and will be responsible for 10% of all deaths globally.

**Table 4:**
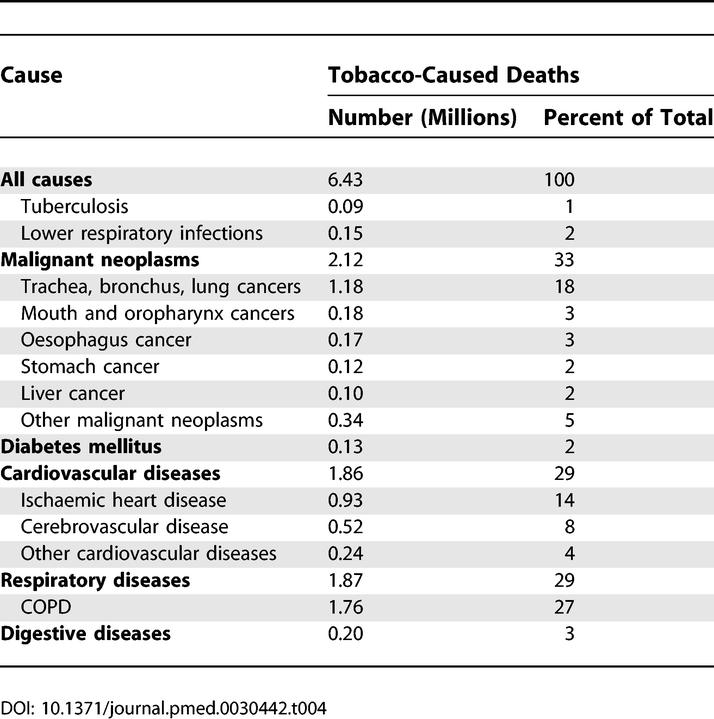
Projected Global Tobacco-Caused Deaths, by Cause, 2015 Baseline Scenario

### Burden of Disease

Global DALYs lost are projected to increase from 1.48 billion in 2002 to 1.54 billion in 2030, an overall increase of only 3%. Since the population increase is projected to be 27% during the same period, there is actually a decrease in the global per capita burden. Unlike deaths, where the overall global death rate is projected to increase by 1%, the DALY rate decreases because the increasing number of deaths is offset by the shift in age at death to older ages, associated with fewer lost years of life. Even with the assumption that the age-specific burden for most nonfatal causes remains constant into the future, and hence that the overall burden for these conditions increases with the ageing of the population, there is still an overall projected decrease in the global burden of disease per capita of 19% from 2002 to 2030.

The proportional contribution of the three major cause groups to the total disease burden is projected to change substantially, however. Group I causes are projected to account for 30% of total DALYs lost in 2030, compared with more than 40% in 2002. In low-income countries, the decline is even greater, from 56% in 2002 to 41% in 2030, even including the doubling of the HIV/AIDS burden. The noncommunicable disease (Group II) burden is projected to increase to 57% in 2030, and to represent a greater burden of disease than Group I conditions in all income groups, including low-income countries.

Table 5 lists the 15 leading causes of DALYs globally in 2002 and in 2030 according to the baseline scenario, and Table 6 provides similar lists of the ten leading causes of burden of disease for high-, middle-, and low-income country groups. The three leading causes of DALYs in the baseline and pessimistic scenarios are projected to be HIV/AIDS, unipolar depressive disorders, and ischaemic heart disease. Road traffic accidents become the third leading cause under the optimistic scenario, ahead of ischaemic heart disease and cerebrovascular disease. Perinatal conditions are the fourth leading cause under the pessimistic scenario and the fifth leading cause under the baseline scenario, after road traffic accidents. HIV/AIDS becomes the leading cause of burden of disease in middle-income countries, as well as low-income countries, by 2015.

**Table 5:**
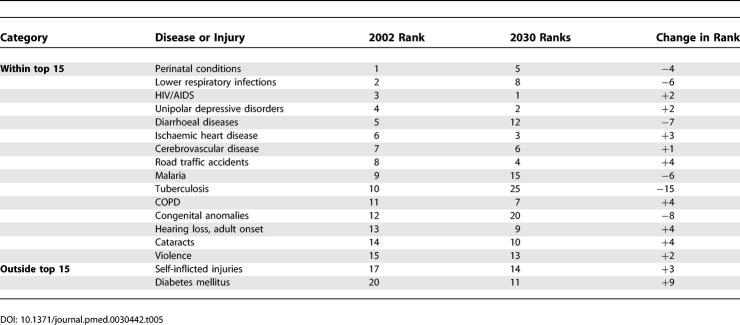
Changes in Rankings for 15 Leading Causes of DALYs, 2002 and 2030 (Baseline Scenario)

**Table 6:**
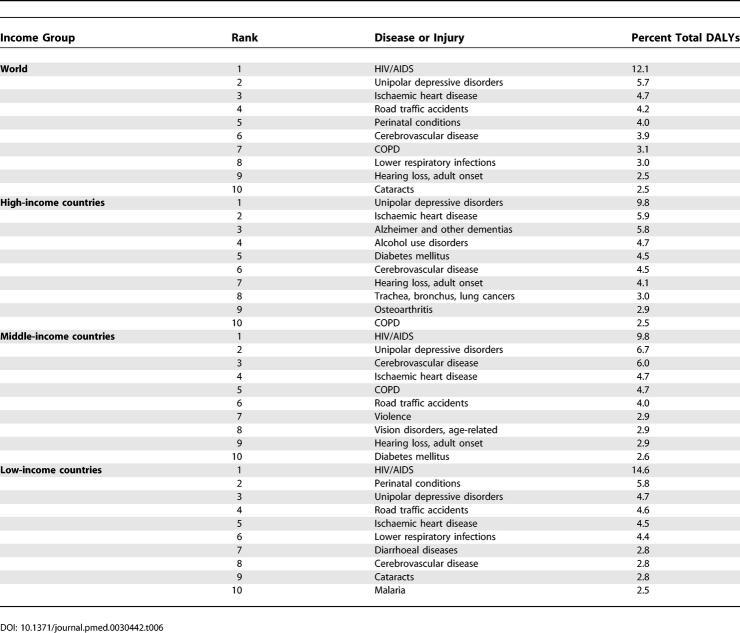
Ten Leading Causes of DALYs, by Income Group and Sex, 2030 (Baseline Scenario)

Table 5 also illustrates the changes in rank order of DALYs between 2002 and 2030 for the 15 leading causes globally. Lower-respiratory infections, perinatal conditions, diarrhoeal diseases, malaria, measles, tuberculosis, and congenital anomalies are all projected to decline substantially in importance. On the other hand, ischaemic heart disease, diabetes mellitus, road traffic accidents, self-inflicted injuries, COPD, hearing loss, and cataracts are all projected to move up three or more places in the rankings. Hearing loss is projected to be among the top ten causes of burden of disease in high- and middle-income countries, and Alzheimer disease and other dementias and alcohol-use disorders among the top four causes in high-income countries in 2030. In low-income countries in 2030, Group I conditions continue to account for five of the ten leading causes of burden of disease. These are HIV/AIDS, perinatal conditions, diarrhoeal diseases, malaria, and acute lower-respiratory infections.

## Discussion

We have addressed the need for updated projections of mortality and burden of disease using methods similar to those of the original GBD study. Our intent was not to undertake major new methodological developments, but rather to provide updated projections for the first 30 years of the 21st century, using as much relevant new information as is available. We project that life expectancy will increase around the world for all three scenarios, fewer children younger than 5 y will die (a 50% decline under the baseline scenario), and the proportion of people dying from non-communicable diseases will increase (from 59% in 2002 to 69% in 2030 under the baseline scenario). Although deaths from infectious diseases will decrease overall, HIV/AIDS deaths will continue to increase; the exact magnitude of the increase will depend to a limited extent on how many people have access to antiretroviral drugs and much more on whether there are increased prevention efforts. Although a projected 6.5 million people will die from HIV/AIDS under the 2030 baseline projection, an even larger number will die from disease attributable to tobacco smoking (8.3 million). By 2030, the three leading causes of burden of disease will be HIV/AIDS, depression, and ischaemic heart disease in the baseline and pessimistic scenarios. Road traffic accidents are the fourth leading cause in the baseline scenario, and the third leading cause ahead of ischaemic heart disease in the optimistic scenario.

These updated projections of mortality and burden of disease have been prepared using a similar methodology to that of the original GBD study, but with some changes described above and with updated inputs and an updated base set of estimates for 2002. We have incorporated a number of methodological improvements and changes. These include carrying out the projections at country rather than regional level, use of separate regression equations for low-income countries, incorporation of information from death registration datasets on recent observed trends for selected causes, and calibration of the regression equations through a comparison of back-projections with observed child mortality trends from 1990 to 2002. Additionally, separate risk factor–based projection models were developed for diabetes mellitus and chronic respiratory diseases. Population projections also included UN projections for net migration rates.

Baseline estimates of deaths, disability, and DALYs lost in 2002 have been projected into the future based on an explicit set of assumptions and methods, and the results are nothing more than the numerical consequences of the assumptions and methods employed. In this paper and the accompanying Protocol S1 and Tables S1–S7, we have tried to explicitly describe and summarize all inputs and assumptions for the three scenarios to enable replication of these projections for different scenarios. Additional information and detailed datasets are available from the authors on request.

We do not claim that the scenarios presented here necessarily represent the best predictions of future global and regional health trends, and it is possible that more sophisticated causal models incorporating projections of important determinants may provide better predictions for specific diseases. However, we do believe that these projections, as with the previous GBD projections of Murray and Lopez, represent the most comprehensive and consistent set of health status projections available today.

### Comparisons with Other Projections

We are not aware of any projections of global and regional mortality, apart from the previous GBD projections, that have included a comprehensive set of causes of death. A review of other published studies containing global and regional projections for specific single causes identified relatively few studies that did not either apply base estimates of age–sex-specific death rates (assumed not to change in the future) to UN population projections, or were based on the previous GBD projections, or were earlier versions of the projections by WHO and/or UNAIDS for HIV/AIDS and tuberculosis used here.

Our projected global population in 2015 under the baseline scenario was 7.1 billion, compared with the UN medium variant projection of 7.2 billion [32], a difference of only −1.6% (see Protocol S1 for details of projection methods). By 2030, the difference between WHO and UN baseline projections of population grew to −3.5%, and the range from pessimistic to optimistic scenarios to 7.75 billion to 8.07 billion. The largest difference in the population growth projections is for the European region, where we project the total population to decline at a faster rate than the UN projections.

Projected global deaths in 2030 ranged from 64.9 million under the optimistic scenario to 80.7 million under the pessimistic scenario, with a baseline projection of 73.2 million. Projected global deaths in 2030 under the UN medium variant projections were 1% higher, at 74.0 million [32]. Our global projections for all-cause mortality are remarkably close to the UN projections, given that our projections are the sum of independent projections for 13 separate cause groups, whereas the UN projections are based on estimated trends in all-cause mortality, with adjustments for projected HIV/AIDS mortality.

Our baseline global projection for all-cause mortality in 2020 (66.5 million deaths) is also remarkably similar to the original GBD baseline projection for 2020 of 68.3 million deaths, although the projected numbers of deaths for each of the three major cause groups differ quite significantly, as do projections for HIV/AIDS and tuberculosis. The congruence in numbers and trends in rates for all-cause mortality is almost certainly a coincidence, since the global total death projections are derived from summing separate projections across 13 cause groups, and almost all of the inputs to those projections have changed significantly from those used in the original projections.

Under our baseline projection, mortality of children younger than 5 y will decline globally by 29% from 2002 to 2015 (range 44% to 14% under the optimistic and pessimistic scenarios). For sub-Saharan Africa the decline is projected to be 25%. In contrast, child mortality declined by only 12% globally and 7% in sub-Saharan Africa between 1990 and 2001 [17]. The more-than doubling of the decline in child mortality for sub-Saharan Africa projected for the next decade is largely determined by the relatively high projected economic growth for this region according to the World Bank (about 2% annual increase in income per capita). Income per capita growth averaged only 0.5% in sub-Saharan Africa during the period 1990–2002. Despite this, our back-projections to 1990 predicted a larger decline in child mortality than observed. The major regression coefficients were adjusted for low-income countries, and in particular for sub-Saharan Africa, to match observed trends (see Protocol S1 for details). Even with this adjustment, we are predicting more than double the decline in child mortality in the next decade compared to that observed in the 1990s. This reflects the higher levels of economic growth projected for the next decade in sub-Saharan Africa.

Trends in tuberculosis death rates projected by the Stop TB Partnership for deaths of HIV-negative persons were used here, as the GBD classifies deaths of HIV-positive persons as due to HIV/AIDS. The GBD base estimates of tuberculosis deaths for 2002 are higher than the Stop TB Partnership 2002 estimates for deaths in sub-Saharan Africa and substantially lower in Southeast Asia (see Protocol S1). As a result of these differences in base estimates, our baseline projection gives 960,000 tuberculosis deaths in 2015, compared to the 888,000 projected by the Stop TB Partnership under the “Sustained DOTS” scenario. Both these estimates are substantially lower than the approximately 2.2 million tuberculosis deaths projected for 2015 by Murray and Lopez in the original GBD study [1,2].

Bray and Møller have published global projections for cancer incidence in 2020 [33]. They projected that total cancer incidence would rise from 11 million new cases in 2002 to 16.5 million in 2020, assuming that 2002 age–sex-specific incidence rates remained unchanged. Taking into account projected changes in age–sex–site-specific incidence rates, we project global incidence for all types of cancer to be 15.5 million cases in 2020, 6% lower than Bray and Møller's estimate. They also estimated global incidence in 2010 of 1.5 million new breast cancer cases and 1 million new prostate cancer cases, taking into account current trends in incidence rates. These are higher than our projections of 1.3 million and 0.85 million, respectively, mainly reflecting differences in the base incidence estimates for 2002.

Wilde et al. [34] estimated that the global diabetes prevalence would rise from 171 million in 2000 to 366 million in 2030, assuming that age–sex-specific diabetes prevalence rates remain unchanged within urban and rural populations in each region, but taking into account projected increases in urbanization to 2030. Their estimate is somewhat higher than our projection of 328 million diabetes cases in 2030. Our projections took into account projected trends for overweight and obesity, which may capture some of the impact of urbanization but did not explicitly account for trends in urbanization. Projections based on prevalence trends have been made for diabetes in Canada and the US [35,36]. These studies estimated that diabetes prevalence would reach 2.4 million for Canada in 2016, and 19.9 million for the US in 2025. Our projections of diabetes prevalence for the same years are one-third higher for Canada (3.2 million) and slightly higher for the US (20.9 million).

Projected tobacco-attributable deaths in 2030 under the baseline scenario are lower than the 10 million predicted by Peto et al. [37] to occur “sometime in the 2020s or early 2030s.” This earlier prediction is fairly consistent with our projection of 9.7 million tobacco-attributable deaths under the pessimistic scenario. Murray and Lopez projected 8.4 million tobacco-attributable deaths in 2020, also somewhat higher than our baseline projection of 7.0 million. Our lower projection reflects our downward adjustment of projected lung cancer mortality to reflect recent trends in tobacco consumption. It must be emphasized that there is very substantial uncertainty in the tobacco-attributable mortality projections, as the trends in apparent consumption of tobacco do not necessarily account for changes in duration, type, per capita amount, and mode of smoking.

**Figure 9:**
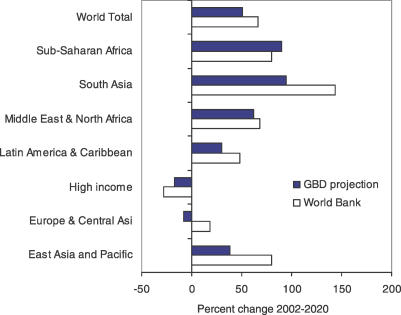
Projected Growth in Road Traffic Fatalities, 2002–2020: A Comparison of World Bank Projections with GBD Projections

A recent World Bank report projected deaths due to road traffic accidents from 1990 to 2020 using an economic model that related death rates to changes in fatalities per motor vehicle and motor vehicle ownership per person due to economic growth [38]. An overall growth of 66% was projected for road traffic fatalities from 2000 to 2020, somewhat higher than our projection of a 51% increase in global deaths due to road traffic accidents from 2002 to 2020 (Figure 9). Projected regional increases were generally similar, although the World Bank projected a substantially greater increase in South Asia than we did, and a small growth for Europe and Central Asia, rather than a decline. In terms of absolute numbers of deaths, the projections are quite different, reflecting large differences in base estimates. The World Bank study estimated global road fatalities in 2000 as 723,000, compared with our estimate for 2002 of 1.2 million. The World Bank base estimates were derived from police statistics, unadjusted for under-reporting. Police statistics are known to generally substantially underestimate deaths due to road traffic accidents. For example, the World Bank estimate of 32,000 deaths for Europe and Central Asia in 2000 is 60% lower than the GBD estimate of 82,000 for 2002, based on reasonably complete death registration data.

### Uncertainties and Limitations

There have been substantial improvements in the data and methods available for the assessment of global and regional mortality by cause. The GBD 2002 estimates of deaths by cause, age, and sex were carried out separately for 226 countries and territories, drawing on a total of 770 country-years of death registration data, as well as 535 additional sources of information on levels of child and adult mortality, and in excess of 2,600 datasets providing information on specific causes of death in regions not well covered by death registration systems [15].

There remain, however, a large number of methodological and empirical challenges to more reliably estimating global, regional, and national disease burden. For regions with limited death registration data, such as the Eastern Mediterranean region, sub-Saharan Africa, and parts of Asia and the Pacific, there is considerable uncertainty in estimates of deaths by cause. Uncertainty ranges for all-cause deaths in 2001 were estimated by Mathers et al. [16] to increase from around ±1% for high-income countries to around ±20% for sub-Saharan Africa. For most specific causes, uncertainty ranges are greater than those of the all-cause mortality estimates, since there is additional uncertainty associated with cause attribution. For example, the relative uncertainty ranges for ischaemic heart disease in 2001 were estimated to range from about 12% for high-income countries to around ±30% for sub-Saharan Africa. The uncertainty range for HIV/AIDS deaths in sub-Saharan Africa was narrower at ±15%, reflecting the substantial data base for these estimates.

The projections of burden are not intended as forecasts of what will happen in the future but as projections of current and past trends, based on certain explicit assumptions. The methods used base the disease burden projections largely on broad mortality projections driven by World Bank projections of future growth in income and WHO projections of increases in human capital in different regions of the world, together with a model relating these to cause-specific mortality trends based on the historical observations in countries with death registration data from the past 50 years. The results depend strongly on the assumption that future mortality trends in developing countries will have a similar relationship to economic and social development as has occurred in the more developed countries. If this assumption is not correct, then the projections for low-income countries will be over-optimistic in the rate of decline of communicable diseases and the speed of the epidemiological transition.

The predictions of the projections model were compared with historical trends in child mortality from 1990 to 2002, and as a result, certain regression coefficients were modified for low-income countries. As a result, our revised projections assume that projected rates of change for cause-specific mortality rates over time, given levels of constant income and human capital, will be slower than those observed in the mainly high- and middle-income countries with death registration data from the past 50 years. This has reduced the projected rates of decline in Group I conditions for low-income countries compared to the original GBD projections, and it is entirely possible that this adjustment may be too conservative. On the other hand, the many problems facing low-income countries in improving and sustaining access to effective health interventions, and in scaling up health systems to cost-effectively address these challenges, may mean that the low-income countries do not experience the temporal pace of health improvement at constant levels of income and human capital that have been seen in the high-income countries in the past 50 years.

The projections have also not taken explicit account of trends in major risk factors apart from tobacco smoking, and, to a limited extent, overweight and obesity. If broad trends in risk factors are for worsening of risk exposures with development, rather than the improvements observed in recent decades in many high-income countries, then again the projections for low- and middle-income countries presented here will be too optimistic. There is a need to develop much more comprehensive projection models that take explicit account of available information on trends in a wide range of risk factors. The HIV/AIDS baseline projections in particular assume that transmission probabilities will remain largely unchanged in the future and there will not be substantial reductions in risk factors for HIV.

A projections exercise such as this by its nature involves substantial assumptions about the similarity of future trends to past trends, and about the future trends in broad drivers of health improvement. There are thus wide uncertainty ranges around future projections. Nevertheless, there are some aspects of the projections that clearly involve more uncertainty than others. For example, the projections of HIV/AIDS mortality are strongly affected by the assumptions made about the levels of additional prevention effort that occur over the next two decades. Additionally, there are substantial uncertainties about the future trends in chronic respiratory disease mortality for non-smokers, and diabetes mortality for persons not overweight. Also, the evidence on the associations of injury mortality with income and human capital was weaker than for Group I and Group II conditions, and stronger assumptions were thus required for injury projections for some external causes. In the absence of any realistic approach to forecasting future war deaths, rates for these were assumed to remain constant over time in the baseline scenario. This may be too conservative, given the substantial decrease in the number of wars and civil conflicts in the past decade or two.

Finally, as did Murray and Lopez, we recognize that the approach taken to projecting YLD is extremely crude, and that the projections of DALYs are likely to be even more uncertain than the projections of deaths. It may be the case that case fatality rates for many diseases decline during the next 30 years, so that YLD become an increasing proportion of the total DALYs for these causes. On the other hand, improvements in risk factors and/or health interventions may lead to decreases in burden for some nonfatal conditions. Substantial research remains to develop robust and unbiased methods for measuring trends in case fatality rates, survival times, and disability due to specific causes, let alone collecting such data across all regions of the world.

### Conclusions

Despite these uncertainties, projections provide a useful perspective on population health trends and health policies, provided that they are interpreted with a degree of caution. Projections enable us to appreciate better the implications for health and health policy of currently observed trends, and the likely impact of fairly certain future trends, such as the ageing of the population, and the continuation of the epidemiological transition in developing countries. These projections represent a set of three visions of the future for population health, under an explicit set of assumptions and for specific projections of income, human capital, and of future trends in tobacco smoking, HIV/AIDS transmission and survival, and overweight and obesity. If the future is not like the past—for example, through sustained and additional efforts to address the Millennium Development Goals, or through major scientific breakthroughs—then the world may well achieve faster progress than projected here, even under the optimistic scenario. On the other hand, if economic growth in low-income countries is lower than the forecasts used here, then the world may achieve slower progress and widening of health inequalities.

## Supporting Information

Alternative Language Text S1 Translation of the Article into French (37 KB DOC)

Dataset S1 Deaths by Cause, Scenario, Sex, and Age for 2002, 2015, and 2030 (1 MB XLS)

Dataset S2 DALYs by Cause, Scenario, Sex, and Age for 2002, 2015, and 2030 (1 MB XLS)

Protocol S1 Technical Appendix (200 KB PDF)

Table S1 Country Classifications Used for Reporting Results: World Bank Regional Groups and World Bank Income Groups (69 KB DOC)

Table S2 GBD Cause Categories and ICD Codes (184 KB DOC)

Table S3 Parsimonious Regression Equations for Nine Major Cause Clusters Based on the Full Country Panel Dataset, 1950–2002 (334 KB DOC)

Table S4 Parsimonious Regression Equations for Nine Major Cause Clusters Based on the Low-Income Country Panel Dataset, 1950–2002 (335 KB DOC)

Table S5 Results of Regressions of Age–Sex-Specific Mortality for Detailed Causes on the Respective Cause Cluster Based on the Full Country Panel Dataset, 1950–2002 (765 KB DOC)

Table S6 Results of Log-Linear Poisson Regressions for Deaths Due to Selected Causes, by Age and Sex, for Countries with Complete Death Registration Data and Population of More Than 5 Million (1.2 MB DOC)

Table S7 Summary of Assumptions and Inputs for Baseline, Optimistic, and Pessimistic Projection Scenarios (67 KB DOC)

## Abbreviations

ART: antiretroviral therapy
COPD: chronic obstructive pulmonary disease
CRA: Comparative Risk Assessment
DALY: disability-adjusted life year
DOTS: directly observed therapy, short-course
GBD: Global Burden of Disease
GDP: gross domestic product
ICD: International Classification of Diseases
*SIR*: smoking impact ratio
UNAIDS: Joint United Nations Programme on HIV/AIDS
YLD: years lived with disability
YLL: years of life lost due to mortality
