# Supplementary material for: Projections of Global Mortality and Burden of Disease from 2002 to 2030
Source: PLoS Med. 2006 Nov 28;3(11):e442. doi: 10.1371/journal.pmed.0030442 (PMC1664601; doi:10.1371/journal.pmed.0030442)
Supplement: Alternative Language Text S1 — (37 KB DOC) [file pmed.0030442.sd001.doc]

**Les projections de la mortalité mondiale et de la charge de morbidité de 2002 à 2030**

**Colin D. Mathers^1^, Dejan Loncar^1^**

**Résumé**

**Objectif**

Les projections globales et régionales de la mortalité ainsi que de la charge de morbidité par cause, pour les années 2000, 2010 et 2030 ont été publiées par Murray et Lopez en 1996 au sein du projet *" Global Burden of Disease"*. Ces projections, basées sur l'année 1990, continuent d'être largement citées, bien qu'étant substantiellement périmées, en particulier parce qu'elles sous-estiment l'étendue de l'épidémie du VIH/SIDA. Afin de répondre à la forte demande d'information pour les tendances futures en santé globale, et soutenir les lignes de conduites en santé internationale pour la mise en place des priorités, nous avons préparé de nouvelles projections de la mortalité et de la charge de morbidité jusqu'en 2030, à partir des estimations de l'Organisation mondiale de la Santé (OMS) pour l'année 2002. Cet article décrit les méthodes, les données, puis les résultats.

**Méthodes et Résultats**

Des modèles relativement simples ont été utilisés pour estimer les tendances futures selon trois scénarios basés en grande partie sur les projections du développement économique et social, en tenant compte des relations historiquement observées entre celles-ci et les taux de mortalité par cause. Les chiffres ont été mis à jour pour intégrer plus de données provenant des registres de décès rendues disponibles, de même que les dernières projections publiées pour le VIH/SIDA, les sources de revenus, le capital humain, le tabagisme et l’indice de la masse corporelle entre autres.

Dans chacun des trois scénarios, il y a un changement dramatique dans la répartition des décès, des âges les plus jeunes aux plus avancés d'une part, et des causes transmissibles, maternelles, périnatales et nutritionnelles aux causes non transmissibles d'autre part. Le risque de mortalité pour les enfants âgés de moins de cinq ans diminuerait presque de 50 % selon le scénario de base, entre 2002 et 2030. On estime que la proportion des décès dus aux maladies non transmissibles va croître de 59 % en 2002 à 69 % en 2030. De plus, globalement, les décès dus au VIH/SIDA s'élèveraient à 2.8 millions en 2002 et augmenteraient jusqu'à 6.5 millions en 2030 toujours selon le scénario de base, qui prend comme hypothèse une hausse de la couverture du traitement antirétroviral à 80 % d'ici l'an 2012. Par contre selon le scénario optimiste, qui suppose également une activité accrue de la prévention, les décès dus au VIH/SIDA baisseraient à 3.7 millions en 2030.

Les décès dus au tabagisme devraient augmenter de 5.4 millions en 2005 à 6.4 millions en 2015 et à 8.3 millions en 2030, selon notre scénario de base. Le tabagisme devrait ainsi tuer 50 % de personnes de plus que le VIH/SIDA en 2015, et, de ce fait, serait responsable de 10 % de toutes les causes de décès en général.

Les trois principales causes de morbidité en 2030, à savoir, le VIH/SIDA, la dépression unipolaire majeure et les maladies cardio-vasculaires sont projetées selon le scénario de base et selon le scénario pessimiste. Les accidents de la route sont la quatrième cause de décès dans notre scénario de base, tandis que dans le scénario optimiste, ils sont à la troisième position, devant les maladies cardio-vasculaires. D'après le scénario de base, le VIH/SIDA passe en tête des causes de morbidité dans les pays aux revenus moyens, comme dans les pays aux revenus faibles et ceci à partit de l'an 2015.

**Conclusion**

Ces projections représentent un ensemble de trois visions en matière de santé de la population, fondées sur certaines hypothèses explicites. Malgré la grande incertitude autour de ces projections, elles nous permettent néanmoins de mieux apprécier les implications dans le domaine de la santé et de la politique sanitaire par rapport aux tendances observées actuellement, et l'impact probable des tendances plausibles, telles que le vieillissement de la population, la diffusion continue du VIH/SIDA dans de nombreuses régions, ou encore la continuation de la transition épidémiologique dans les pays en voie de développement. Les résultats dépendent fortement de l'hypothèse que l'évolution future de la mortalité dans les pays pauvres suit le même rapport avec le développement économique et social que dans les pays à revenus plus élevés.
