# Supplementary material for: Projections of Global Mortality and Burden of Disease from 2002 to 2030
Source: PLoS Med. 2006 Nov 28;3(11):e442. doi: 10.1371/journal.pmed.0030442 (PMC1664601; doi:10.1371/journal.pmed.0030442)
Supplement: Protocol S1 — (200 KB PDF) [file pmed.0030442.sd004.pdf]

New projections of global mortality and burden of disease from 2002 to 2030

Colin D. Mathers, Dejan Loncar

Protocol S1. Technical Appendix

The original Global Burden of Disease (GBD) study used a set of relatively simple models to project future health trends, based largely on projections of economic and social development, and using the historically observed relationships of these with cause-specific mortality rates [1,2]. Rather than attempt to model the effects of the many separate direct determinants or risk factors for disease from the limited data that are available, the GBD methodology considered a limited number of socio-economic variables: average national income per capita, average years of schooling in adults, and time. In addition, a fourth variable, tobacco use, was included in the projections for cancers, cardiovascular diseases and chronic respiratory diseases, because of its overwhelming importance in determining trends for these causes.

The updated projections have essentially used the same approach, with updated regression models based on substantially more data, updated projections for regression covariates and for HIV/AIDS mortality, and a number of variations and enhancements of the original methodology. As with the original GBD projections, we have developed three projection scenarios with baseline, pessimistic and optimistic projections of covariates in the regression equations and corresponding pessimistic and optimistic choices for other parameters and assumptions. The revised projection methods involves six separate analytical or computational steps documented in this technical appendix.

Major cause regressions

We projected age and sex specific death rates at country level for ten major cause clusters (defined in Table 1), with separate projection models for males and females and for seven age groups: 0-4 years, 5-14, 15-29, 30-44, 45-59, 60-69 and 70 years and over. The GBD broad cause group category 'Group I' shown in Table 1 comprises communicable, maternal, perinatal and nutritional conditions, and 'Group II' comprises non-communicable diseases including mental disorders: the full GBD cause list is defined in terms of ICD-9 and ICD-10 codes of the International Classification of Diseases in Table S2. Diabetes mellitus was treated as a separate cause category since the available evidence suggests that its dominant risk factor, overweight and obesity, is becoming more prevalent over time in both developed and developing regions, and is projected to continue to rise. In contrast, age-specific death rates for 'Other Group II' diseases are generally projected to decrease with continuing economic development. Projections for HIV/AIDS mortality were carried out separately.

We used a regression equation of the same form as in the original GBD projections:

$$\ln M_{a,k,i} = C_{a,k,i} + \beta_1 \ln Y + \beta_2 \ln HC + \beta_3 (\ln Y)^2 + \beta_4 T + \beta_5 \ln SI_{a,k} \qquad (1)$$

where $C_{a,k,i}$ is a constant term, $M_{a,k,i}$ is the mortality level for age group a, sex k, and cause i, and Y, HC and T denote income per capita, human capital and time, respectively. The log of the smoking impact SI is included in the equation only for malignant neoplasms, cardiovascular diseases, and respiratory diseases. With this functional form, the elasticity of the mortality rate with respect to GDP per capita and human capital is constant, as is the rate of change of mortality with respect to time. Hence, for instance, a 1 per cent change in per-capita income leads to a β_1 per cent change in the mortality rate for the particular age-cause group. The squared term for the log of GDP per capita is also included in the equation in order to allow for further non- linearity in the relationship between age-, sex- and cause-specific mortality and these independent variables.

Table 1. Nine major cause clusters used for estimation of parsimonious regression models

Major cause cluster	Cause	GBD cause code (see Table S2 for definitions)
1	Group I excluding HIV	W001excluding W009
2	Malignant neoplasms	W060
3	Cardiovascular diseases	W104
4	Digestive diseases	W115
5	Respiratory diseases	W111
6	Other Group II (excluding diabetes)	W078, W080, W081, W098, W120, W124, W125, W131, W143
7	Road traffic accidents	W150
8	Other unintentional injuries	W151, W152, W153, W154, W155
9	Intentional injuries	W156
*	Diabetes mellitus	W079

This basic functional relationship makes no specific assumptions about the relationships between these more distal socio-economic determinants and more proximate determinants of mortality rates such as environmental, life style and physiological risk factors. Nevertheless, the regression results presented below indicate that a considerable proportion of the variance in age-, sex- and cause-specific mortality rates can be explained by this limited set of distal determinants.

Income per capita, measured in international dollars, and adjusted for differences in purchasing power not captured in official exchange rates, is used as a general proxy for many aspects of development. Equally importantly, research has consistently shown that education is an important distal determinant of health status [3–5]. Following Murray and Lopez, we use the average number of years of schooling of the population above the age of 25 to reflect average education levels, and. refer to this variable as “human capital”. Estimates of income per capita and human capital have been prepared by the World Health Organization (WHO) for all WHO Member States for the years 1950-2002 as described in the following Section.

In addition, a fourth variable, tobacco use, was included in the projections for cancers (malignant neoplasms), cardiovascular diseases and chronic respiratory diseases, because of its overwhelming importance in determining trends for these causes. Smoking prevalence rates from community surveys do not well predict the overall health impact of smoking, since they do not take account of the long lag times between exposure and many disease outcomes or important aspects of exposure such as duration, type, amount and mode of smoking. Tobacco use was thus measured in terms of “smoking impact” - that component of observed lung cancer mortality that is attributable to tobacco smoking [6]. Smoking impact was calculated from the death registration data for each country-year, by subtracting estimated non-smoker lung cancer mortality rates from the overall lung cancer mortality rates. This smoking impact variable was included in the regression equations for malignant neoplasms, cardiovascular diseases and chronic respiratory diseases for males and females aged 30 years and older.

The fourth independent variable used in the projections is time itself. Technology has profoundly changed in the health sector over the last 50 years and continues to change with substantial investments in research and development. Obtaining a measurement of technological change is not only difficult (such data are usually not available) but also controversial. Following Murray and Lopez, we used calendar year as a proxy measure of the impact of technological change on health status.

Murray and Lopez used a variety of econometric approaches to estimate the equations of this form, including methods which take into account auto-correlation and heteroscedasticity. Because these methods substantially restricted the subset of panel data which could be used, and in particular resulted in much loss of information for moderate to high mortality populations, they chose to use ordinary least squares (OLS) regression based on the entire dataset for the final set of parameter estimates. We follow the same approach

Death registration dataset

The original GBD projections used a panel dataset of 1,394 country-years based on death registration data from 47 countries for the years 1950-1990. For these new projections, death registration data from 106 countries for the years 1950-2002 were used to develop the regression equations. For many countries, the data series was incomplete. In total, 2,605 observation years were available, almost double those used for the original projections. The dataset includes most countries of Europe, the Americas, Australia, New Zealand and Japan, as well as a number of Eastern Mediterranean, Asian and African countries with useable death registration data. Country-years of death registration data were distributed as follows: high income countries (1261 country-years), other European countries (531), Latin America and the Caribbean (609), Eastern Mediterranean (36), sub-Saharan Africa (44), South East Asia (25), middle and low income countries of the Western Pacific (89).

Although the dataset is extensive, it does not include many observations from populations with high rates of mortality. The original GBD projections used only observations considered reasonably complete and reliable, and the applications of predictive equations based on a largely high income country dataset to regions such as sub-Saharan Africa is obviously highly tentative. For the revised projections, all observations for which levels of completeness of child and adult death registration could be estimated were included after adjustments for incomplete registration. A number of regression models were run to test the sensitivity of the projection results to the completeness adjustments. Regressions were rerun on a dataset where country-years with estimated levels of adult completeness below 90% were excluded. Regressions were also rerun with observations weighted according to level of completeness.In addtion, the analyses were also carried out with observations weighted by the inverse of the square root of the number of deaths in the observation (to reflect greater uncertainty associated with smaller numbers of deaths)These alternate regressions did not result in any substantial change in the resulting projections for major cause groups.

In order to estimate mortality for the major cause clusters over a long period of time, causes of death coded according to the 6th, 7th, 8th, 9th and 10th Revisions of the International Classification of Disease (ICD) were analysed and a mapping of ICD codes for each of the nine cause-clusters across the five revisions of the ICD was developed. Additional pre-processing of country-year death registration data included redistribution of deaths with unknown age and redistribution of deaths coded to certain ill-defined cause categories in accordance with the GBD methodology [7]. For the major cause regressions, country-year observations were also excluded where there were less than 10 deaths for the age-sex-cause category, or where the total male plus female population of all ages was less than 50,000.

Whereas the original GBD projections applied a single set of models based on all observed death registration data for projections in all regions, our revised projections have used a second set of models for low income countries based on the observed relationships for a low income data set consisting of 1,734 country-years of observation where GDP per capita was less than $10,000. This limit was chosen as all low income countries in 2002 (GDP per capita less than $3,000) had projected GDP per capita of less than $10,000 in 2030.

Selection of parsimonious predictive equations

Following the same criteria as the original GBD projections, parsimonious equations were developed based on two criteria. First, if the sign for a variable was consistent with our prior hypothesis, but the parameter estimate was not significant at the 5 per cent level, this variable was excluded from the model. If the sign of a parameter estimate was the opposite of our prior hypothesis, then the variable was excluded from the model if the parameter estimate was not significant at the 1 per cent level. In the case where the coefficient for $\ln Y$ was not significant, but that for $(\ln Y)^2$ was, then the term for $(\ln Y)^2$ was dropped from the equation first. Two variables were dropped only if an F-test for the combination of variables was not significant. Tables S3 and S4 summarize the final parsimonious regression equations for each of the major cause clusters for the fourteen age-sex groups.

The regression result for low income countries (table S4) gave somewhat more conservative declines for Group II in low income regions. We applied the low income regression betas for all countries in sub-Saharan Africa and, in other regions, for countries with GDP per capita in 2002 less than 3,000 international dollars.

In general, these equations are broadly similar to those estimated for the original projections. Apart from injuries, they explain a surprising proportion of the variance for many age-sex-cause categories. For males and females aged 70 and above, the R^2 for many cause-clusters was generally lower than for other age groups, probably reflecting poorer quality of coding of causes of death or the smaller range of variation in mortality rates between countries at these older ages. The lower proportions of variance explained for injuries may reflect lower levels of temporal variation in these causes as well as more heterogeneous injury causes that may be less correlated with income and human capital.

In the original GBD projections, it was assumed that the age-sex specific mortality rate will stay constant for those causes where the R^2 was less than 10 per cent. In addition, constant rates were assumed for intentional injuries despite R^2 values as high as 0.30 for females aged 45-59 years, due to implausibly rapid predicted rises and the generation of implausible age-patterns. For most of these groups in the current regression results, the projected trends by region were consistent with those for other age groups. So to preserve age structure, we have left these regression betas in the projections.

We carefully examined the regional trends resulting from the application of the regression results for the injury cause groups, and decided to use the regression equations for road traffic accidents and unintentional injuries. Due to low R^2 for older age groups, we assumed unintentional injury rates were constant for males aged 70 and over, and for females aged 60 and over. For intentional injuries, we assumed constant death rates for males aged 0-4, 60-69, 70 and over, and for females aged 0-4. 45-59, 60-69 and 70 and over.

Model predictions of age-, sex- and cause-specific mortality rates in 2002 for each of the ten clusters of causes were compared for each country with the results of the Global Burden of Disease Study for that year. A series of scalars were then derived so that projected values for 2002 were identical to the 2002 GBD results. It was then assumed that these scalars would remain constant over the period 2002-2030.

Diabetes and chronic respiratory diseases

Initial regression analysis for diabetes mellitus found inconsistent trends between males and females, probably reflecting the large variations across countries and inaccuracies in recording diabetes as underlying cause of death in many death registration systems. While overall trends for diabetes mortality showed no consistent relationship across the sexes or levels of development, the regression analysis found significant betas for T (year) with death rates increasing with time.

For this reason, a separate projection model for diabetes mortality was developed using a recent analysis of the relative risk of diabetes mortality with increasing prevalence of overweight and obesity (as measured by body mass index) [8] and projections of population distributions of body mass index (BMI) [9]. This model was based on the finding that one half or more of global diabetes mortality was attributable to high BMI in 2000: 50% for males and 66% for females. In the European region and other high income countries around 80% or more of diabetes mortality was attributable to high BMI relative to a counterfactual normal distribution of BMI with mean 21 and standard deviation 1 kg/m^2 [8].

For a population aged a and sex k, with BMI distribution $P_{a,k}(x)$, where x is the BMI level, the relative risk of diabetes mortality compared with a population with the counterfactual BMI distribution of $P'_{a,k}(x)$ is given by:

$$RR_{a,k} = \int_{x=0}^{m} RR_{a,k}(x) P_{a,k}(x)dx - \int_{x=0}^{m} RR_{a,k}(x) P'_{a,k}(x)dx \qquad (2)$$

where $RR_{a,k}(x)$ is relative risk of diabetes mortality at BMI level x, and m is the maximum BMI level [10].

We used WHO projections of BMI distributions by country [9] to estimate the relative risk of diabetes mortality in future year t, for ages 30 and over, compared to the CRA counterfactual BMI distribution, as follows:

$$RR_{a,k,t} = \int_{x=10}^{61} \exp\left[\ln(rr_{a,k}) \times \left(x - 21 - \frac{x - m_{a,k,t}}{sd_{a,k,t}} \right)\right] \times N\left(\frac{x - m_{a,k,t}}{sd_{a,k,t}} \right) dx \qquad (3)$$

where $rr_{a,k}$ is the relative risk of diabetes mortality for a one unit increase in BMI. BMI distributions in year t were assumed to be normally distributed with mean $m_{a,k,,t}$and standard deviation $sd_{a,k,,t}$. James et al. reviewed the evidence on the association of diabetes mortality with BMI and found that a constant relative risk of diabetes mortality for each one unit increase in BMI. fitted the available data well for each age-sex group [8]. The estimated $rr_{a,k}$ ranged from around 1.4 and 1.5 for males and females aged 30-44 respectively to around 1.2 in males and females in older age groups.

Country-specific projections of BMI distributions for WHO Member States from 2000 to 2010 were used [9]. Projected trends in mean BMI were assumed to flatten between 2010 and 2015 and to be constant beyond 2015. For the baseline projections, the diabetes mortality rate associated with the counterfactual BMI distribution (mean mean 21 and standard deviation 1 kg/m^2) was assumed to be declining at three-quarters the rate for 'Other Group II' causes on the assumption that the rate of decline for the latter group reflected not only treatment improvements but to some extent improvements in risk factor exposures not relevant for diabetes. The rate of decline was

varied for the optimistic and pessimistic scenarios to 100% and one half of the rate of decline for 'Other Group II' causes respectively.

Initial projections for chronic respiratory diseases resulted in substantially increasing rates for high income countries. However, smoking is the main risk factor for chronic obstructive pulmonary disease (COPD) in high income countries and has been declining in most of these countries. It is likely that the initial projections may reflect increasing propensity to code COPD as underlying cause of death with time, particularly with shift to use of ICD-10 in the 1990s. Recent analyses of the mortality risk of COPD associated with tobacco smoking [11,12] were used, together with projections of smoking impact, to project future trends in COPD mortality. Similar projections were carried out for asthma and other chronic respiratory diseases, where the smoking attributable fractions are much smaller. The non-smoker rates for all these chronic respiratory diseases were assumed to be declining with socioeconomic growth at three-quarters the rate for 'Other Group II' causes. This assumption was varied for the optimistic and pessimistic scenarios as for diabetes.

Projections of income per capita, human capital, smoking impact and technological change

Revised country-level projections were developed for income per capita, human capital and smoking impact. In addition, the regression betas for time, reflecting technological change, were modified for low income countries as described below.

Income per capita projections

Income per capita was measured using average GDP per capita expressed in international dollar terms. WHO has prepared consistent estimates of GDP per capita in international dollars for the years in the 1990s through to 2001; for details refer to the Statistical Annex Notes of the World Health Report 2004 [13]. For earlier years, income series for WHO Member States were estimated using information from the Penn World Tables [14,15] and, for some missing years, using growth rates of real GDP per capita in local currency units. International dollar estimates were derived by dividing local currency units by an estimate of their purchasing power parity (PPP) compared to US dollars. PPPs are the rates of currency conversion that equalise the purchasing power of different currencies by eliminating the differences in price levels between countries.

Country-specific and regional income growth forecasts by the World Bank were used to project GDP per capita for all WHO Member States. Country-specific projections were used for 144 countries for the period 2002-2015. For other countries, relevant World Bank regional growth rates for the period 2002-2012 were applied to the country-specific GDP per capita for 2002. Beyond 2015, projected growth rates for most regions approach 3% per annum, with somewhat lower growth rates in sub-Saharan Africa, the Middle East and high income countries (around 2.5%). Table 2 summarizes the resulting regional average annual growth rates in GDP per capita by World Bank region for the periods 2002-2005, 2006-2015, and 2015-2030. For the optimistic scenario, growth rates in GDP per capita were assumed to be approximately 40% higher than the baseline projection, and for the pessimistic scenario to be around 50% of the growth rate in the baseline projection (Table 2).

Table 2. Projected average annual growth rates in GDP per capita, by World Bank region, 2002-2030

	World Bank Region						
Scenario/Period	East Asia and Pacific	Europe and Central Asia	High Income	Latin America and Caribbean	Middle East & N. Africa	South Asia	Sub-Saharan Africa
**Baseline**							
2002-2005	6.4	5.9	2.3	2.0	2.9	4.7	2.0
2006-2015	5.4	3.6	2.4	2.4	2.6	4.2	2.1
2015-2030	3.7	3.0	2.5	2.7	2.5	3.3	2.5
**Pessimistic**							
2002-2005	3.2	0.5	1.0	1.0	0.5	2.0	0.0
2006-2015	2.7	1.0	1.2	1.2	1.0	2.0	0.5
2015-2030	1.5	1.5	1.5	1.5	1.5	1.5	1.0
**Optimistic**							
2002-2005	7.0	6.0	2.9	2.6	3.2	5.2	2.5
2006-2015	7.5	5.0	3.4	3.4	3.7	4.8	3.0
2015-2030	4.5	4.0	3.5	3.8	3.5	4.4	3.5

Human capital projections

Human capital was measured in terms of the average years of schooling in the adult population as constructed by Barro and Lee [16]. Revised estimates and projections of human capital for WHO Member States have been prepared by WHO for the period 1950-2030 drawing on the Barro-Lee estimates for 98 countries for five-yearly intervals from 1950 to 1990 and observed relationships between growth in human capital and growth in GDP. In order to fill in the historical estimates for countries for which average years of schooling were not available from Barro-Lee, a regression was run with average years of schooling as the dependent variable and lagged 15-year primary school enrollment, lagged 10-year secondary school enrollment, lagged 10-year tertiary school enrollment, and current literacy as independent variables. For some countries and for some years, average years of schooling were available from an alternate source: the Demographic and Health Survey (DHS). In such cases, this was also used as an explanatory variable and predictions generated for those years and those countries for which DHS data were available were used to scale the entire panel of years.

In the dataset for 1950-2002, there was a clear relationship between the growth rate in human capital and levels of income and human capital. OLS regression was used to estimate the following equation, pooling 2,733 observations for both males and females, with R^2 of 43%:

$$r = -0.006 + 0.00137 * HC + 0.00476 * \ln Y - 0.00053 * HC * \ln Y \qquad (4)$$

where r is the the growth rate in human capital *HC,* and *Y* is income per capita. For typical low income countries, with ln*Y* around 8 and *HC* around 5, the average annual growth rate in human capital is around 2 per cent per annum.

This equation was used to fill in some missing *HC* values for some countries for early years, and also to generate the three scenarios for human capital projections. For the optimistic and pessimistic scenarios, we arbitrarily modified the equation so that the coefficient for *HC* was 0.001 higher or lower, resulting in around 30% higher or lower annual growth rate in *HC* for a typical

low income country. Table 3 summarizes projections of human capital for the three scenarios by World Bank income group.

Table 3. Projections of human capital by sex and income group, for three scenarios: 2002-2030

	World Bank Region						
Scenario/Period	East Asia and Pacific	Europe and Central Asia	High Income	Latin America and Caribbean	Middle East & N. Africa	South Asia	Sub-Saharan Africa
**Baseline**							
2002-2005	6.4	5.9	2.3	2.0	2.9	4.7	2.0
2006-2015	5.4	3.6	2.4	2.4	2.6	4.2	2.1
2015-2030	3.7	3.0	2.5	2.7	2.5	3.3	2.5
**Pessimistic**							
2002-2005	3.2	0.5	1.0	1.0	0.5	2.0	0.0
2006-2015	2.7	1.0	1.2	1.2	1.0	2.0	0.5
2015-2030	1.5	1.5	1.5	1.5	1.5	1.5	1.0
**Optimistic**							
2002-2005	7.0	6.0	2.9	2.6	3.2	5.2	2.5
2006-2015	7.5	5.0	3.4	3.4	3.7	4.8	3.0
2015-2030	4.5	4.0	3.5	3.8	3.5	4.4	3.5

Smoking impact projections

Smoking impact (SI) was calculated for the historical mortality country-year observations by subtracting non-smoker lung cancer rates from observed total lung cancer mortality rates in the data. Higher non-smoker lung cancer mortality rates were used in China and South East Asian countries reflecting the higher levels of lung cancer in non-smokers due to indoor air pollution from exposure to smoke from solid fuels [17]. Ezzati and Lopez developed SI projections for 14 sub-regions of the WHO regions, based on an analysis of past and current age-sex-specific smoking prevalence and trends in per capita tobacco consumption calibrated to regional characteristics of the tobacco epidemic for different subregions [17,18].

Detailed age-sex-specific SI projections at country level ideally require historical information on trends in age-sex-specific tobacco consumption, both in terms of proportions of the population smoking, and in terms of quantity and types of tobacco consumed by smokers. Shibuya et al. have carried out such projections for four high income countries (Australia, Canada, United Kingdom and the United States of America) using a three-factor age-period-cohort model for lung cancer mortality [19]. The SI projections for these four countries were used for 2002 to 2030 under all three scenarios.

For other countries, where such detailed age-period-cohort projection models were not available, the subregional SI projections of Ezzati and Lopez were used to prepare country-level projections, by shifting the regional projections with a variable time lag to match the base SI estimates for each country for 2002. Any differences between the regional SI pattern and the country base SI estimates for 2002 that could not be accommodated by adjusting the time lag, were adjusted by scaling the regional SI estimates. These country-specific estimates were then used for the pessimistic scenario.

Recent data on country-specific trends in adult per capita consumption of cigarettes [20] were used to prepare a separate set of age-sex-specific SI trends assuming a 25-year time lage between consumption and SI. For the baseline scenario, country-specific SI projections were prepared using

the average of the annual trends for these SI projections and those prepared using the subregional projections of Ezzati and Lopez. In other words, the regional-based projections were adjusted to some extent by using the most recently available country-specific information on trends in apparent tobacco consumption. For many countries, the SI projections based on apparent consumption trends were lower than those based on the regional projections. For the optimistic scenario, country-specific SI projections were based on the apparent consumption trends where they gave lower SI levels, and on the regional-based projections where they gave lower SI levels.

For all three scenarios, the resulting country-specific SI projections were further adjusted for countries with recent death registration data using estimated trends in age-sex-specific lung cancer mortality. Annual trends were estimated for age-sex-specific lung cancer mortality using log-linear Poisson regression models. Country-specific SI projections were adjusted for these countries by applying revised age-sex-specific annual SI trends calculated as a weighted sum of the trend estimated from the country mortality data and the trend projected under the scenario. The weight given to the trend estimated from the death registration data decreased from 100% for 2003, to 75% for 2004, and decreasing by 25% for each subsequent year. By 2015 the weight given to the Poisson trend estimate declines to 5%.

The resulting projections of smoking impact (SI) under the pessimistic, baseline and optimistic scenarios are shown in figure 1 by sex and country income group. For some regions, the baseline SI projections are substantially lower than the pessimistic SI projections (based on the previous regional projections of Ezzati and Lopez), reflecting flat or declining per capita consumption trends in these regions. It is possible that tobacco consumption has not increased as fast as previously projected, perhaps in part due to global tobacco control efforts, but it is also possible that the apparent consumption trends understate the true levels of tobacco exposure, and that the pessimistic scenario is more realistic. These differences between scenarios affect mainly the projections for lung cancer and chronic lung diseases. Projections for cardiovascular diseases, and other diseases caused by tobacco smoking, are less sensitive to these variations in projections, in part because tobacco smoking is a less dominant risk factor (that is, there are a number of other major risk factors for these diseases also), and in part, because the broad trends in the projections are also dominated by population ageing.

Modification of regression parameters for low income countries

The original GBD projections assumed that the improvement in mortality rates with time, holding socioeconomic conditions constant, that had been observed in the historical data drawn largely from middle income and high income countries, applied in low income countries such as those of sub-Saharan Africa. This assumption has been retained for the optimistic projection scenario. For the baseline scenario, we tested this assumption by using the regression equations to back-project child mortality under age 5 years from 2002 to 1990 and to compare the regional results with the observed regional trends in child mortality for 1990 to 2002 [13,21].

For low income countries, the regression equations predicted a greater decline in child mortality from 1990 to 2002 than was observed, and the difference was greater for sub-Saharan Africa than for other low income countries. For middle and high income countries, the observed and predicted trends were reasonably consistent. We attempted to improve the regression predictions for low income countries by adjusting the regression coefficients for time, and if needed, also for human capital. A reasonable fit to the observed trends for 1990-2002 was achieved by halving the regression coefficients for human capital for low income countries, and setting the time coefficients to zero for sub-Saharan Africa and, for other low income countries, to one quarter of the regression estimates. We used these adjustments for low income countries in the baseline scenario and varied them for the optimistic and pessimistic scenarios as summarized in table S7.

Figure 1: Projections of smoking impact (SI) by sex and income group, for three scenarios: baseline (solid lines), optimistic (dashed lines) and pessimistic (dotted lines).

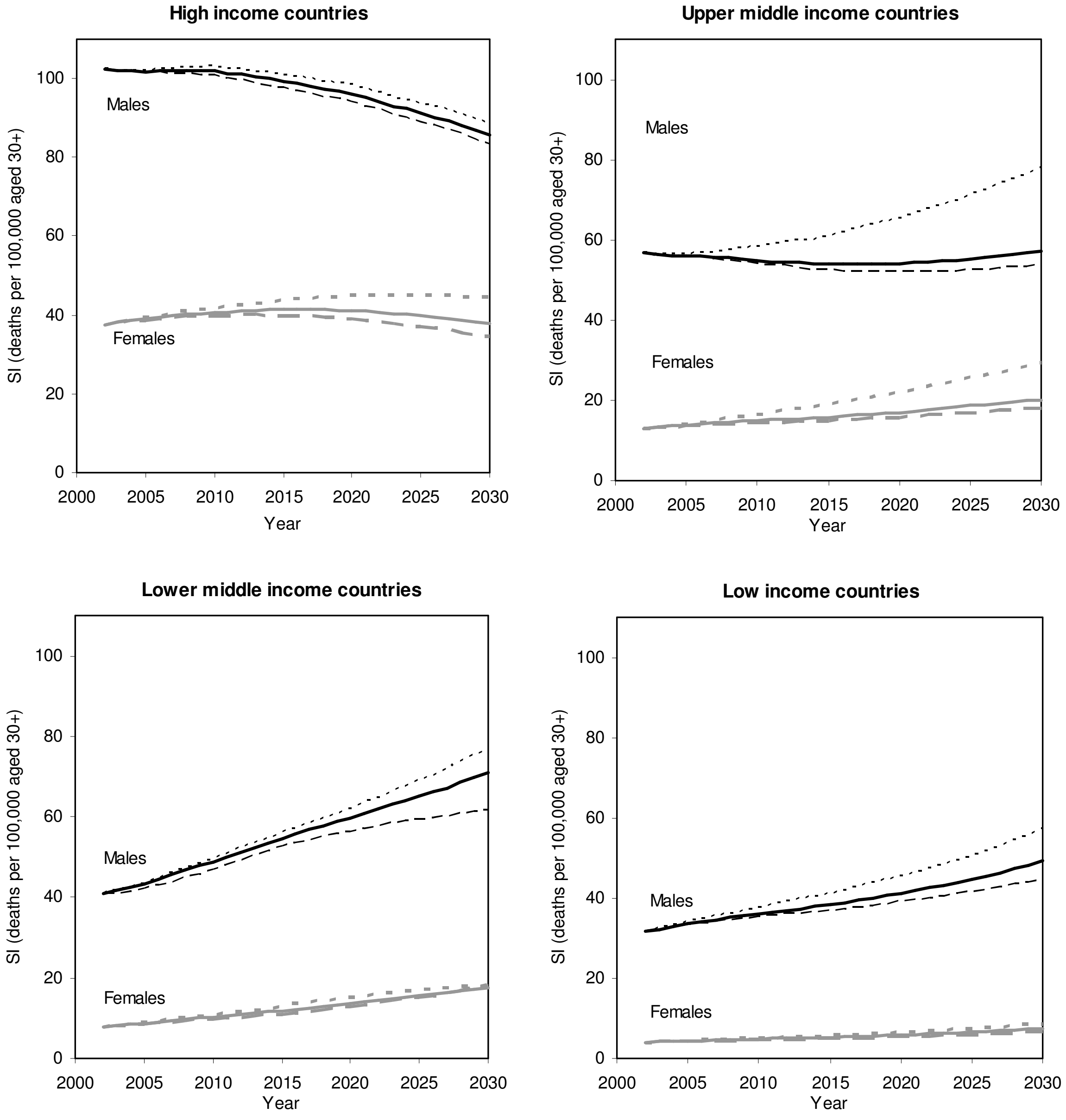

For the pessimistic scenario, the regression coefficients for both time and human capital were set to zero for low income countries. For the optimistic scenario, the regression coefficients for human capital were set to three quarters of the regression values for low income countries. For sub-Saharan African countries in the optimistic scenario, the coefficients for time were set to one quarter of the regression values, and for other low income countries to three quarters of the regression values.

Projections for detailed cause categories

For the original GBD projections, Murray and Lopez chose not to attempt to model the relationship between GDP per capita, human capital, smoking impact and time, with more specific causes of death such as tuberculosis, liver cancer etc, due to the difficulties of comparing detailed cause-of-death data coded according to different revisions of the International Classification of Diseases. Instead, they applied a variant of the Preston cause-of-death model approach, which models the relationship of the trend for a detailed cause of death with the trend for the broader cause group containing it.

Regression equations for detailed causes

We followed the original GBD approach in estimating the following relationships:

$$\ln M_{a,k,i,d} = \beta_{a,k,i,d} \ln M_{a,k,i} + C_{a,k,i,d} \quad (5)$$

where $M_{a,k,i,d}$ is the mortality rate from specific cause *d* within cause group *i* for age group *a* and sex *k*, $M_{a,k,i}$ is the mortality rate for cause group *i* for age group *a* and sex *k*, and $\beta_{a,k,i,d}$ is the coefficient from the OLS regression. These constant elasticity regressions provide estimates of the percentage change in death rates from a specific case *d* for a given percentage change in the mortality rate for a broader cause-cluster *i*.

To avoid biasing the results due to coding changes associated with use of different versions of ICD, we restricted the analysis to country-years for which deaths were coded using ICD-9. The regression parameters were estimated on 1,357 country-year observations for 98 countries. For each individual regression, observations were excluded from analysis if they related to less than 50 deaths. Following the original projection methods, we use the regression results only where the relationship was reasonably strong as measured by an R^2 greater than 0.25 and a β coefficient with a p-value <0.001. The resulting coefficients are shown in table S5. For any cause-age-sex groups not shown in these tables, the β coefficient was set to 1.

The predicted detailed age-, sex-, country- and cause-specific mortality rates were adjusted so that they summed across all causes d within cause cluster *i* to the total projected age-, sex-, and country-specific mortality rates for cause cluster *i*.

Modifications for detailed causes in the intentional injury cause cluster

The intentional injury cause cluster contains three detailed cause: suicide, homicide and war (we ignore the other intentional injury category which contains negligible estimated deaths). The major cause cluster regression equation for intentional injury predicted generally falling death rates in middle and low income countries and rising death rates in high income countries. For homicide (deaths due to intentional violence), this is the opposite of the observed trends in the larger high

income countries. For this reason, homicide death rates were assumed to remain constant under the baseline scenario (and modified by country-specific observed trends as described below). Similarly, war deaths were assumed to remain constant under the baseline scenario.

For the optimistic scenario, homicide death rates were held constant in high income countries (and modified by country-specific observed trends), but allowed to follow the intentional injury projected trends for middle and low income countries. For the pessimistic scenario, projected trends for intentional injuries were assumed to apply for all countries.

For the optimistic scenario, war death rates were assumed to decline at 1.5% per annum from 2006 onwards. For the pessimistic scenario, war death rates were assumed to have risen between 2002 and 2005 for the Eastern Mediterranean and the United States, reflecting largely the impact of the wars in Afghanistan and Iraq, and then to remain constant over time in all regions.

Adjustments for recent observed trends for selected causes

For high income countries with populations of 5 million or more, and with at least 5 years of recent death registration data, cause-age-sex specific trends in mortality rates were estimated for ischaemic heart disease, cerebrovascular disease, tuberculosis, suicide and homicide using a log-linear Poisson regression model for country-years of data from 1985 onwards, and with an exponentially decreasing weight for earlier country years (each earlier year given 0.85 times the weight of the next year in the regression). This weighting scheme was chosen to give greater weight to the trends in more recent years.

The resulting estimates for recent annual trend rates are shown in table S6. These estimated trends by cause, age and sex were used to adjust the initial years of projection for these causes for the selected countries. The country-specific trends were given 100% weight for the first projection year (2003), then a weight decreasing by a factor of 0.85 for each subsequent year, until by 2015 the trend is fully determined by the projection models trends. This adjustment ensured that available country-specific information on recent trends in mortality was incorporated into the projections for selected important causes.

At the global level, the largest impacts of these adjustments were for ischaemic heart disease (around 4% increase in projected deaths in 2015) and for homicide (around 2% increase in projected deaths in 2015). At the level of income groups, the largest impacts were for males in high income countries: a 15% decrease for tuberculosis deaths in 2015, a 10% decrease in cerebrovascular disease deaths in 2015, and a 16% decrease in homicide deaths in 2015.

Projections for HIV/AIDS and TB

UNAIDS and WHO [22] have prepared projections of HIV/AIDS mortality under a range of assumptions about the future of the HIV epidemics in all regions and with varying treatment scale-up assumptions both for adult and children using a transmission model previously used to assess the impact of preventive interventions [23,24]. Projected prevalence curves were based on current country-specific models for 2004 and assumptions about future trends in prevalence in 125 low-income and middle-income countries [25,26]. For countries with generalized epidemics (mostly in Sub-Saharan Africa), the model variables were estimated from sentinel site prevalence data. In most cases the projected epidemics remained stable.

For countries with epidemics concentrated in groups with higher risk behaviour, the size and HIV prevalence was estimated for each of these groups and prevalence in low-risk populations was estimated by allowing for transmission from high-risk to low-risk groups via sexual mixing.

Projections of these epidemics were based on assumptions about degree of saturation for each of the high-risk groups, time to saturation, and spread from high-risk to low-risk populations over time

Baseline adult antiretroviral treatment (ART) coverage estimates for each scenario were based on December 2004 coverage estimates published by WHO and UNAIDS [27,28]. Three additional scenarios assuming fast, medium or slow increase in coverage of ART, PMTCT programs and CTX treatment were applied to project future trends for the HIV epidemic (Table 4). For each scenario the maximum coverage (i.e., the percent of individuals in need of treatment who receive it) was fixed at a certain level and year, while assuming a linear increase from current coverage. Similar scenarios were applied to children ART and co-trimoxazole (CTX) coverage. The treatment scenarios assumed no effect of treatment on transmission and incidence rates, and no additional prevention efforts resulting in reductions of transmission and incidence rates. Under these scenarios, there will be between 4.3 and 4.8 million deaths due to AIDS in 2015, rising to 6.5 - 6.6 million in 2030.

Table 4: Peak coverage (and year) for HIV/AIDS scenarios for different regions in the world

Region	Model	Africa	Asia	Eastern Europe	Latin America and Caribbean
	Slow	60 (2012)	60 (2012)	60 (2012)	70 (2013)
Adult ART coverage (%)	Medium	80 (2012)	80 (2012)	80 (2012)	80 (2012)
	Fast	80 (2009)	80 (2009)	80 (2009)	80 (2007)
	Slow	60 (2012)	60 (2012)	60 (2012)	70 (2013)
Child ART coverage (%)	Medium	80 (2012	80 (2012)	80 (2012	80 (2012)
	Fast	80 (2009)	80 (2009)	80 (2009)	80 (2007)
	Slow	60 (2012)	60 (2012)	60 (2012)	60 (2012)
PMTCT coverage (%)	Medium	80 (2012)	80 (2012)	80 (2012)	80 (2012)
	Fast	80 (2009)	80 (2009)	80 (2009)	80 (2009)
	Slow	60 (2012)	60 (2012)	60 (2012)	70 (2013)
Co-trimoxazole coverage (%)	Medium	80 (2012)	80 (2012)	80 (2012)	80 (2012)
	Fast	80 (2009)	80 (2009)	80 (2009)	80 (2009)

Baseline projections

Baseline projections for HIV/AIDS mortality rates were based on the UNAIDS and WHO "medium" scenario for treatment scale-up. Under this scenario, ART coverage reaches 80% by 2012 in all regions (Table 4). For the 67 mainly high income countries not included in the UNAIDS and WHO scenario projections, we assumed zero trends in HIV/AIDS mortality for these countries. For small countries with middle or low income, AIDS death rates were assumed to follow the same time trend as regional projections.

The GBD estimates of HIV/AIDS mortality for 2002 were revised to reflect country-level estimates consistent with those published for 2003 in the 2004 Report on the Global AIDS Epidemic [25], with the exception of certain countries, including Russia and Brazil, where HIV/AIDS mortality estimates were based on a detailed analysis of death registration data for recent years.

Pessimistic scenario

The pessimistic projections for HIV/AIDS age-sex specific mortality rates were based on the UNAIDS and WHO "slow" scenario for treatment scale-up. Under this scenario, ART coverage reaches 60% by 2012 in all regions except Latin America, where it reaches 70% in 2013 (Table 4). HIV/AIDS mortality rates in high income countries were assumed to remain constant as in the baseline scenario.

Optimistic scenario

Salomon et al. [29] have modeled scenarios for sub-Saharan Africa combining treatment and additional prevention efforts. In one scenario, ART coverage is scaled up and optimal assumptions are made about treatment impact on transmissibility and patient behaviour. A second scenario assumes that an emphasis on treatment leads to less effective implementation of prevention resulting in only 25% attainment of the maximum potential impact of prevention efforts. For our optimistic projection of HIV/AIDS mortality we used a third unpublished scenario prepared by Salomon et al. which is approximately halfway between their two published mixed treatment-prevention scenarios. It was not possible to directly use their projected mortality rates, because the ART scale-up scenario that they used differed somewhat from the UNAIDS and WHO medium scenario described above, with ART coverage reaching 80% in 2010 rather than 2012. We thus estimated the additional impact of the prevention effort on annual mortality trends in sub-Saharan Africa for the period 2003 to 2020 by comparison of Salomon et al.'s treatment-only scenario with the combined treatment-prevention scenario. The additional impact of the modeled prevention effort does not significantly affect HIV/AIDS mortality rates until 2008, after which there is an additional annual decline rising to 3% in 2015, and remaining reasonably constant in the range 3.1-3.3% to 2020.

For preparing the HIV/AIDS projections for the optimistic scenario, we applied these additional regional annual declines for years 2008 and beyond to the baseline projections of HIV/AIDS mortality rates for countries in sub-Saharan Africa. For low and middle income countries in other regions, we applied additional annual rates of decline rising from 0.1% in 2005, to 0.5% in 2008, to 3% in 2015, and remaining constant at 3% for subsequent years. HIV/AIDS mortality rates in high income countries and other regions were assumed to remain constant until 2008, and then to start declining with an annual rate of decline increasing to 3% in 2015, and remaining constant for subsequent years.

Tuberculosis projections

Because of the powerful interaction between tuberculosis and HIV infections in regions such as sub-Saharan Africa, Murray and Lopez modified the original projections from 1990 to 2020 for tuberculosis death rates. They assumed that the increased case-load due to tuberculosis in HIV-positive persons was likely to influence the annual risk of infection of tuberculosis in HIV-negative persons in the community. The increasing global burden of tuberculosis during the 1990s has been linked to the the HIV/AIDS epidemic, and there is some evidence that the increased burden of HIV-associated TB cases also increases TB transmission rates at the community level [30]. Estimated total global tuberculosis deaths among HIV-negative persons descreased by 20 per cent from 1.96 million in 1990 [2] to 1.57 million in 2002, but in sub-Saharan Africa where the HIV epidemic has been strongest, estimated tuberculosis deaths in HIV-negative persons fell from 386,000 in 1990 to 348,000 in 2002, only a 10 per cent decrease. In African countries with an HIV prevalence >4 per cent in adults aged 15-49, tuberculosis incidence and mortality rates for HIV-negative persons increased by an estimated 150 per cent and 90 per cent respectively between 1990 and 2003 [31].

The Stop TB Partnership has prepared three projection scenarios for tuberculosis incidence, prevalence and mortality covering the period 2006-2015 based on different assumptions about the pace of scale-up and coverage of interventions under the Global Plan to Stop TB [32]. Scenarios were developed globally and for seven of the eight TB epidemiological regions: African countries with high HIV prevalence (AFR High), African countries with low HIV prevalence (AFR Low), Latin American and Caribbean countries (LAC), Eastern European region (EEUR), Eastern Mediterranean region (EMR), South East Asian region (SEAR), and Western Pacific Region (WPR). These regions are defined in an Annex of the Global Plan report. Projection scenarios were not developed for the Established Market Economies (EME) and Central Europe, considered together as one epidemiological region with low tuberculosis incidence and mortality rates.

Estimates were made of TB case detection and treatment outcomes over the next ten years, as well as of TB prevalence, incidence and death rates in HIV-negative and HIV-positive persons. Published projections related to HIV-negative and HIV-positive persons combined [32]. Unpublished projected incidence, prevalence and death rates by TB epidemiological region for HIV negative persons were obtained from the Stop TB Partnership for the following three scenarios:

1. “Global Plan” scenario: This assumes massive scale-up in tuberculosis control activities, achieving case detection levels of over 70% and cure rates over 85%. The first new TB drug for 40 years will be introduced in 2010, sensitive and inexpensive diagnostic tests will be available in 2010-2012, and a new cost-effective vaccine will be available by 2015.
2. “Sustained DOTS” scenario: case detection and treatment success rates increase until 2005 and then remain constant to 2030.
3. “No DOTS” scenario: This assumes that the DOTS treatment strategy was never introduced in any region, so case detection, treatment and cure rates would continue as they were pre-DOTS, with variable rates of case detection and typically lower rates of cure.

Figure 2 shows the projected tuberculosis death rates in HIV-negative persons under the three scenarios, by TB epidemiological region. These projections were used to project tuberculosis death rates forward to 2030 from the Global Burden of Disease estimates for 2002 as follows:

Baseline scenario: Projected annual trends in tuberculosis death rates for TB epidemiological regions for 2003 to 2015 under the “Sustained DOTS” scenario were applied to Global Burden of Disease 2002 estimates of tuberculosis death rates for each country within each TB epidemiological region. For the EME plus CE region, tuberculosis mortality was assumed to decline at the rates predicted by the regression equations for Group I conditions excluding HIV/AIDS under the baseline scenario. For years 2016 to 2030, regional annual trends were projected to linearly converge on the baseline projected annual trend for 2030 for Group I causes excluding HIV/AIDS.

Optimistic scenario: Projected annual trends in tuberculosis death rates for TB epidemiological regions for 2003 to 2015 under the “Global Plan” scenario were applied to Global Burden of Disease 2002 estimates. For the EME plus CE region, tuberculosis mortality was assumed to decline at the rates predicted by the regression equations for Group I conditions excluding HIV/AIDS under the optimistic scenario. For years 2016 to 2030, annual trends from 2015 to 2030 were assumed to remain constant at the 2015 level.

Figure2: Regional projections of tuberculosis death rates per 100,000 population, for three scenarios: "Sustained DOTS" (solid lines), "Global Plan" (dashed lines) and"No DOTS" (dotted lines). Source: unpublished projections for HIV negative cases by the Stop TB Partnership.

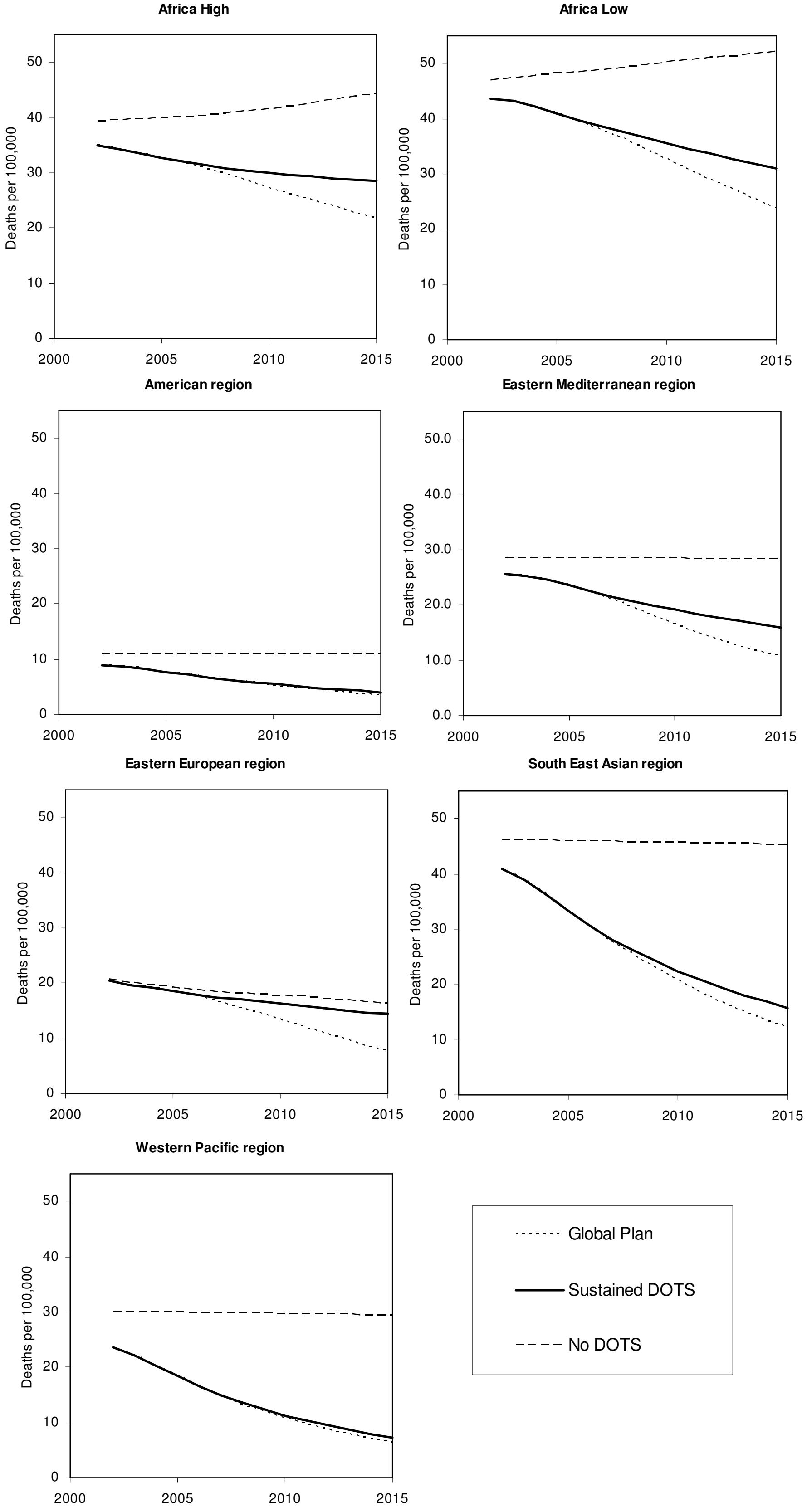

Pessimistic scenario: Annual trends were estimated as halfway between those projected under the "sustained DOTS" scenario and those projected under the "No DOTS" scenario from 2006 onwards. For the EME plus CE region, tuberculosis mortality was assumed to decline at the rates predicted by the regression equations for Group I conditions excluding HIV/AIDS under the pessimistic scenario. For years 2016 to 2030, annual trends from 2015 to 2030 were assumed to remain constant at the 2015 level.

Numbers of tuberculosis deaths under the three scenarios of the Global Plan projections are shown in Table 5 and compared with the final projected numbers of tuberculosis deaths under the baseline, optimistic and pessimistic scenarios. It must be emphasised that, for all regions other than EME & CE (Established Market Economies and Central Europe), the regional annual trends in death rates for HIV-negative cases are identical for the two sets of projections. The differences in projected numbers of tuberculosis deaths in 2015 are due to diffeences in the base estimates of TB deaths in the two sets of projections. The GBD estimates of tuberculosis deaths in 2002 are substantially higher for Sub-Saharan Africa (by over 100,000 deaths) and substantially lower for South East Asia (by 50,000 deaths). The Stop TB Partnership has revised downwards its base estimate of HIV-negative tuberculosis deaths in Sub-Saharan Africa, although the total tuberculosis deaths in HIV-negative and HIV-positive persons remains similar. As can be seen in Figure 2, TB death rates are now estimated to be lower in high HIV prevalence African countries than in low HIV prevalence African countries, although the projected trend is for slower decline in the high HIV countries.

Table 5. Comparison of projected numbers of tuberculosis deaths (000s) by region for the three scenarios of the Stop TB Partnership and the current projections starting from GBD 2002 tuberculosis mortality estimates.

Year/Scenario	AFR High	AFR Low	EEUR	EME & CE	EMR	LAC	SEAR	WPR	World[1]
**Global Plan projections**									
**2002**									
Global Plan	177	80	79	-	131	48	649	erge366	1,546
Sustained DOTS	177	80	79	-	131	48	649	366	1,546
No DOTS	200	86	80	-	145	59	731	468	1,785
**2015**									
Global Plan	144	60	29	-	71	22	229	109	680
Sustained DOTS	190	78	56	-	106	25	296	125	888
No DOTS	294	131	64	-	188	69	851	501	2,109
**Final TB projections**									
**2002**									
All scenarios	282	94	58	16	109	45	599	361	1,565
**2015**									
Optimistic	228	71	35	12	62	23	203	105	739
Baseline	298	92	56	13	92	26	262	120	960
Pessimistic	344	112	60	15	115	35	392	197	1,270
**2030**									
Optimistic	134	38	7	9	23	8	54	29	303
Baseline	282	81	27	10	56	12	106	44	618
Pessimistic	407	128	34	12	105	22	267	111	1,087

1 World totals include estimated deaths for the EME & CE region based on the projections for this region shown in the bottom half of the table.

Because of these differences in base estimates, our baseline projection gives 960,000 tuberculosis deaths in 2015 compared to the 888,000 projected under the "Sustained DOTS" scenario. Both these estimates are substantially lower than the approximately 2.2 million tuberculosis deaths projected for 2015 by Murray and Lopez in the original GBD study [1,2].

Projecting years lived with disability

The World Health Organization has undertaken new assessments of the Global Burden of Disease for the years 2000 to 2002, with consecutive revisions and updates published annually in WHO's World Health Reports. These assessments use a health gap measure, the Disability Adjusted Life Year (DALY), developed by Murray and Lopez [33] to quantify the equivalent years of full health lost due to diseases and injury in WHO Member States. The DALY combines years of life lost from premature death (YLL) and years of life lived with disability (YLD) in a single indicator allowing assessment of the total loss of health from different causes. The data sources and methods used for the GBD 2002 are documented elsewhere [34–36] and summary results for 14 regions of the world are published in the World Health Report 2004 [13] and on the world wide web (www.who.int/evidence/bod).

The GBD estimates for 2002 have been used as a base for projections of burden of disease to 2030. From the projections of mortality by cause, age and sex, it is a simple matter to calculate projected Years of Life Lost (YLL). However, in order to project DALYs, it is also necessary to project Years Lived with Disability (YLD). Given the lack of good information on trends in disability and health state distributions, the approach used here to project YLDs is an elaboration of the methods and assumptions used by Murray and Lopez in the original GBD projections [2].

Methods and assumptions for cause-specific YLD projections

Disease and injury causes were divided into three distinct categories, and a different method used to approximate YLDs for each category:

- For causes with significant case-fatality (see table 6), incidence rates and YLD rates were generally assumed to change in line with projected mortality rates. In other words, age-sex-specific death/incidence ratios were assumed to remain constant, and average durations and disability weights also remain constant For ischaemic heart disease and stroke, future incidence rates were assumed to decline at 50% of their mortality rate declines¸ in other words the mortality rate decline was assumed to result from declines in incidence rates and case fatality rates. For long-term sequelae associated with certain cancer sites, incidence rates were projected to increase with improvements in income per capita in line with the cross-regional variations seen in the 2002 estimates.

- For non-communicable disease causes without significant mortality (table 6), age-sex-specific incidence and prevalence rates were generally assumed to remain constant into the future. For certain mental disorders, hearing loss and musculoskeletal disorders, disability weights were assumed to decline with improvements in income per capita in line with the cross-regional variations seen in the 2002 estimates (reflecting higher treatment coverage in higher income countries).

- The prevalences of non-fatal communicable diseases and nutritional deficiencies were assumed to decline at between 50% to 100% of the mortality rate declines for Group I causes. The prevalences of cataract and age-related vision disorders were assumed to decline at 100% and 25% respectively of the mortality rate declines for Group II causes.

HIV/AIDS was treated separately, as the WHO and UNAIDS Working Group provided incidence and prevalence projections, as well as mortality projections. For the calculation of YLD, the duration for HIV seropositive cases not yet progressed to AIDS was assumed to remain constant, and the duration for cases that have progressed to AIDS was assumed to double between the years 2005 and 2012. For the optimistic scenario, the incidence of HIV was assumed to be decreased by the same ratio as the mortality for HIV ten years later.

Table 6. Methods and assumptions used to project cause-specific YLDs.

Broad category	Projection method*	Causes
**A. Incidence/ mortality ratio**	Age-sex-country specific incidence to mortality ratios assumed to remain constant into the future	Tuberculosis, pertussis, diphtheria, measles, tetanus, meningitis, hepatitis B, hepatitis C, malaria, lower respiratory infections, maternal conditions other than obstructed labour, perinatal conditions, site-specific malignant neoplasms, other malignant neoplasms, diabetes mellitus, endocrine disorders, Parkinson disease, rheumatic heart disease, hypertensive heart disease, inflammatory heart disease, other cardiovascular diseases, chronic obstructive pulmonary disease, other respiratory diseases, digestive diseases, nephritis and nephrosis, other genitourinary system diseases, abdominal wall defect, anencephaly, anorectal atresia, oesophageal atresia, renal agenesis, congenital heart anomalies, spina bifida, other congenital anomalies, all injury cause categories
	Incidence rates decline at 50% of mortality rates	Ischaemic heart disease, cerebrovascular disease
	Inc/mortality ratio for ages 30 and over adjusted as in accordance with GDP projections (see text)	Long-term cancer sequelae for the following sites: colon and rectum, breast, cervix, uterus, ovary, prostate, bladder.
**B. Constant rates**	Age-sex-country specific incidence rates assumed to remain constant into the future	Upper respiratory infections, other neoplasms, unipolar depressive disorders, bipolar affective disorder, schizophrenia, epilepsy, alcohol use disorders,Alzheimer and other dementias, multiple sclerosis, drug use disorders, post-traumatic stress disorder, obsessive-compulsive disorder, panic disorder, insomnia (primary), migraine, mental retardation attributable to lead exposure, other neuropsychiatric disorders, glaucoma, other sense organ disorders, asthma, benign prostatic hypertrophy, skin diseases, musculoskeletal conditions, other musculoskeletal disorders, cleft lip, cleft palate, Down syndrome, oral conditions
	Age-sex-country specific disability weights for ages 15 and over adjusted to decline with increasing GDP (see text)	Bipolar affective disorder, schizophrenia, epilepsy, obsessive-compulsive disorder, panic disorder, migraine, adult-onset hearing loss, rheumatoid arthritis, osteoarthritis, gout
**C. Declining rates**	Constant annual decline in age-sex-country -specific incidence rates	Poliomyelitis (10% per annum), leprosy (5% per annum), lead-caused mental retardation (2% per annum)
	Decline at 100% of Group I mortality excluding HIV and TB	Tropical-cluster diseases, dengue, Japanese encephalitis, trachoma,
	Decline at 75% of Group I mortality excluding HIV and TB	Intestinal nematode infections, other infectious diseases, otitis media, nutritional deficiencies
	Decline at 50% of Group I mortality excluding HIV and TB rates	Sexually transmitted diseases excluding HIV, diarrhoeal diseases, obstructed labour
	Decline with Group II mortality	Cataract (100%), age-related vision disorders (25%)
**D. Incidence projections**	Explicit projection models used also for incidence, prevalence and durations	HIV/AIDS

* Where not specifically mentioned, average durations and disability weights used in the calculation of YLDs are assumed to remain constant.

The detailed assumptions used for the projection of YLDs for each cause are summarized in Table 6. We recognize that the approach taken to projecting YLDs is extremely crude, and that the projections of DALYs are likely to be even more uncertain than the projections of deaths. Substantial research remains to develop robust and unbiased methods for measuring trends in case fatality rates, survival times and disability due to specific causes, let alone collecting such data across all regions of the world.

Population projections

Future population growth and changes in age composition are determined by levels and trends in three factors: fertility, mortality and migration. To ensure consistency between the projected mortality rates and the projected population numbers, we have used our projections of mortality rates, together with UN medium variant assumptions for fertility rates and migration rates [37], to prepare consistent population projections for all regions.

Cause-specific mortality projections were first aggregated to obtain estimates of all-cause mortality in each of the seven age groups for each country. Mortality rates for these seven age groups were then used to estimate mortality rates for each five-year age group from 0-4 through to 95 and over, using the ratio of the five-year age group mortality rate to the larger age band mortality rate, based on the GBD estimates of mortality by country for 2002. UN Population Division 2002 revision medium variant projections of fertility rates by maternal age and country were applied to projected female population numbers in each of the scenarios in order to project the size of the birth cohort for each country for future years. Population projections for each five-year age group were then obtained using the projected birth and death rates, and appying the UN Population Division 2002 revision medium variant projections of net migration rates by age, sex and country.

The projected global population in 2015 under the baseline scenario was 7.1 billion compared to the UN medium variant (2002 revision) projection of 7.2 billion, a difference of -1.4%. The range in the projected global population in 2015 was from 7.06 billion (pessimistic scenario) to 7.13 billion (optimistic scenario). By 2030, the difference between WHO and UN baseline projections of population grew to 3%, and the range from pessimistic to optimistic scenarios to 7.75 - 8.07 billion. The largest difference in the population growth projections is for the European region, where we project the total population to decline at a faster rate than the UN projections.

Summary

Table S7 summarizes the assumptions and inputs for the optimistic, baseline and pessimistic scenarios. These were described in more detail in earlier sections of this Technical Protocol.

Detailed results for 2002, 2015 and 2030 are presented in Dataset S1 for deaths and Dataset S2 for DALYs. These tables include baseline, pessimistic and optimistic projected totals for each cause category, as well as a sex and age breakdown for the baseline category.

Reference List

1. Murray CJL, Lopez AD (1997) Alternative projections of mortality and disability by cause 1990-2020: Global Burden of Disease Study. Lancet 349: 1498-1504.

2. Murray CJL, Lopez A.D. (1996) Alternative visions of the future: projecting mortality and disability, 1990-2020. In: Murray CJL, Lopez AD, editors. The Global Burden of Disease. Cambridge: Harvard University Press. pp. 325-397.

3. Caldwell JC (1979) Education as a factor in mortality decline: an examination of Nigerian data. Population Studies 41: 5-30.

4. Caldwell J, Caldwell P (1991) What have we learnt about the cultural, social and behavioral determinants of health? From selected readings to the first health transition workshop. Health Transition Review 1: 3-20.

5. Cleland J, van Ginneken J (1988) Maternal education and child survival in developing countries. Social Science & Medicine 27: 1357-1368.

6. Peto R, Lopez AD, Boreham J, Thun M, Heath JC (1992) Mortality from tobacco in developed countries: indirect estimation from National Vital Statistics. Lancet 339: 1268-1278.

7. Murray CJL, Lopez AD (1996) Estimating causes of death: new methods and global and regional applications for 1990. In: Murray CJL, Lopez AD, editors. The Global Burden of Disease. Cambridge: Harvard University Press. pp. 118-200.

8. James WPT, Jackson-Leach R, Ni Mhurchu C, Kalamara E, Shayeghi M, et al. (2004) Overweight and obesity (high body mass index). In: Ezzati M, Lopez A, Rodgers A, Murray CJL, editors. Comparative quantification of health risks: global and regional burden of disease attributable to selected major risk factors. Geneva: WHO. pp. 959-1108.

9. WHO Global Infobase Team (2005) The SuRF Report 2. Surveillance of chronic disease risk factors: country-level data and comparable estimates. Geneva: WHO.

10. Murray CJL, Ezzati M, Lopez AD, Rodgers A, Vander Hoorn S (2004) Comparative quantification of health risks: conceptual framework and methodological issues. In: Ezzati M, Lopez A, Rodgers A, Murray CJL, editors. Comparative quantification of health risks: global and regional burden of disease attributable to selected major risk factors. Geneva: WHO. pp. 1-38.

11. Ezzati M, Lopez AD (2003) Estimates of global mortality attributable to smoking in 2000. The Lancet 362: 847-852.

12. Ezzati M, Lopez AD (2004) Regional, disease specific patterns of smoking-attributable mortality in 2000. Tob Control 13: 388-395.

13. World Health Organization (2004) World Health Report 2004: Changing History. Geneva: WHO.

14. Summers R, Heston A (1991) The Penn World Table (Mark 5): An expanded set of international comparisons, 1950-1988. Quarterly Journal of Economics 106: 327-368.

15. Heston A, Summers R, Aten B (2002) Penn World Table Version 6.1.

16. Barro RJ, Lee JW (1996) International measures of schooling years and schooling quality. American Economic Review 86: 218-223.

17. Ezzati M, Lopez AD (2004) Smoking and oral tobacco use. In: Ezzati M, Lopez A, Rodgers A, Murray CJL, editors. Comparative quantification of health risks: global and regional burden of disease attributable to selected major risk factors. Geneva: WHO. pp. 959-1108.

18. Ezzati M, Lopez AD (2003) Measuring the accumulated hazards of smoking: global and regional estimates for 2000. Tob Control 12: 79-85.

19. Shibuya K, Inoue M, Lopez AD (2005) Statistical modeling and projections of lung cancer mortality in 4 industrialized countries. International Journal of Cancer 117: 476-485.

20. World Bank (2005) Economics of Tobacco Control: Country Data. http://www1.worldbank.org/tobacco/database.asp.

21. Ahmad O, Lopez AD, Inoue M (2000) The decline in child mortality: a reappraisal. Bulletin of the World Health Organization 78: 1175-1191.

22. UNAIDS-WHO Working Group on Global HIV-AIDS-STI surveillanceRegional projections of the HIV/AIDS epidemic incorporating the effect of antiretroviral treatment, prevention of mother to child transmission programs and co-trimoxazole treatment for low and middle income countries, 2004-2015.Unpublished report. Geneva: UNAIDS.

23. Stover J, Walker N, Garnett GP, Salomon JA, Stanecki KA, Ghys PD, Grassly NC, Anderson RM, Schwartlander B (2002) Can we reverse the HIV/AIDS pandemic with an expanded response? Lancet 360: 73-77.

24. Walker N, Stanecki KA, Brown T, Stover J, Lazzari S, Garcia-Calleja JM, Schwartländer B, Ghys PD (2003) Methods and procedures for estimating HIV/AIDS and its impact: the UNAIDS/WHO estimates for the end of 2001. AIDS 17: 2215-2225.

25. UNAIDS (2004) 2004 report on the global AIDS epidemic. Geneva: UNAIDS.

26. UNAIDS Reference Group on Estimates Model and Projections. (2002) Improved methods and assumptions for estimation and projection of HIV/AIDS epidemics. AIDS 16: 1-16.

27. WHO and UNAIDS (2004) "3 by 5" Progress Report. Geneva: World Health Organization and UNAIDS.

28. USAID, UNAIDS, WHO, UNICEF and the POLICY Project (2004) Coverage of selected services for HIV/AIDS prevention, care and support in low and middle income countries in 2003 . Washington DC: POLICY Project.

29. Salomon JA, Hogan D, Stover J, Stanecki KA, Walker N, Ghys PD, Schwartlander B (2005) Integrating HIV prevention and treatment: from slogans to impact. PLoS Medicine 2: e16.

30. Corbett EL, Watt CJ, Walker N, Maher D, Williams BG, Raviglione MC, Dye C (2003) The growing burden of tuberculosis: global trends and interactions with the HIV epidemic. Archives of Internal Medicine 163: 1009-1021.

31. World Health Organization (2005) Global Tuberculosis Control: Surveillance, Planning and Financing: WHO Report 2005. Geneva: World Health Organization.

32. Stop TB Partnership (2006) The global plan to stop TB, 2006-2015. Geneva: World Health Organization.

33. Murray CJL, Lopez AD, (eds) (1996) The Global Burden of Disease: a comprehensive assessment of mortality and disability from diseases, injuries and risk factors in 1990 and projected to 2020. Cambridge: Harvard University Press.

34. Mathers CD, Lopez AD, Murray CJL (2006) The burden of disease and mortality by condition: data, methods and results for 2001. In: Lopez AD, Mathers CD, Ezzati M, Murray CJL, Jamison DT, editors. Global burden of disease and risk factors. New York: Oxford University Press. pp. 45-240.

35. Mathers CD, Salomon JA, Ezzati M, Begg S, Lopez AD (2006) Sensitivity and uncertainty analyses for burden of disease and risk factor estimates. In: Lopez AD, Mathers CD, Ezzati M, Murray CJL, Jamison DT, editors. Global burden of disease and risk factors. New York: Oxford University Press. pp. 399-426.

36. Lopez AD, Mathers CD, Ezzati M, Jamison DT, Murray CJL (2006) Global and regional burden of disease and risk factors, 2001: systematic analysis of population health data. Lancet 367: 1747-1757.

37. United Nations Population Division (2003) World Population Prospects - the 2002 revision. New York: United Nations.
