# Supplementary material for: Projections of Global Mortality and Burden of Disease from 2002 to 2030
Source: PLoS Med. 2006 Nov 28;3(11):e442. doi: 10.1371/journal.pmed.0030442 (PMC1664601; doi:10.1371/journal.pmed.0030442)
Supplement: Table S1 — (69 KB DOC) [file pmed.0030442.st001.doc]

Table S1: Country classifications used for reporting results: World Bank regional groups (rows) and World Bank income groups (columns).

| ***Income category^1^*** | ***High income*** | | |
| --- | --- | --- | --- |
| ***High income^2^*** | Andorra, Aruba, Australia, Austria, Bahamas, Bahrain, Belgium, Bermuda, Brunei Darussalam, Canada, Cayman Islands, Channel Islands, Cyprus, Denmark, Faeroe Islands, Finland, France, French Polynesia, Germany, Greece, Greenland, Guam, Iceland, Ireland, Israel, Italy, Japan, Kuwait, Liechtenstein, Luxembourg, Monaco, Netherlands, Netherlands Antilles, New Caledonia, New Zealand, Northern Mariana Islands, Norway, Portugal, Qatar, Republic of Korea, San Marino, Singapore, Slovenia, Spain, Sweden, Switzerland, United Arab Emirates, United Kingdom, United States of America, United States Virgin Islands | | |
|  | ***Upper middle income*** | ***Lower middle income*** | ***Low income*** |
| ***East Asia and Pacific*** | American Samoa, Malaysia, Palau | China, Fiji, Kiribati, Marshall Islands, Micronesia (Federated States of), Philippines, Samoa, Thailand, Tonga, Vanuatu | Cambodia, Democratic People's Republic of Korea, Indonesia, Lao People's Democratic Republic, Mongolia, Myanmar, Papua New Guinea, Solomon Islands, Timor-Leste, Viet Nam |
| ***Europe and Central Asia*** | Croatia, Czech Republic, Estonia, Hungary, Isle of Man, Latvia, Lithuania, Poland, Slovakia | Albania, Belarus, Bosnia and Herzegovina, Bulgaria, Kazakhstan, Romania, Russian Federation, Serbia and Montenegro, The former Yugoslav Republic of Macedonia, Turkey, Turkmenistan | Armenia, Azerbaijan, Georgia, Kyrgyzstan, Republic of Moldova, Tajikistan, Ukraine, Uzbekistan |
| ***Latin America and Caribbean*** | Antigua and Barbuda, Argentina, Barbados, Brazil, Chile, Costa Rica, Dominica, Grenada, Mexico, Panama, Puerto Rico, Saint Kitts and Nevis, Saint Lucia, Trinidad and Tobago, Uruguay, Venezuela | Belize, Bolivia, Colombia, Cuba, Dominican Republic, Ecuador, El Salvador, Guatemala, Guyana, Honduras, Jamaica, Paraguay, Peru, Saint Vincent and the Grenadines, Suriname | Haiti, Nicaragua |
| ***Middle East and North Africa*** | Lebanon, Libyan Arab Jamahiriya, Malta, Oman, Saudi Arabia | Algeria, Djibouti, Egypt, Iran (Islamic Republic of), Iraq, Jordan, Morocco, Occupied Palestinian Territory, Syrian Arab Republic, Tunisia | Yemen |
| ***South Asia*** |  | Maldives, Sri Lanka | Afghanistan, Bangladesh, Bhutan, India, Nepal, Pakistan |
| ***Sub-Saharan Africa*** | Botswana, Gabon, Mauritius, Seychelles | Cape Verde, Namibia, South Africa, Swaziland | Angola, Benin, Burkina Faso, Burundi, Cameroon, Central African Republic, Chad, Comoros, Congo, Côte d'Ivoire, Democratic Republic of the Congo, Equatorial Guinea, Eritrea, Ethiopia, Gambia, Ghana, Guinea, Guinea-Bissau, Kenya, Lesotho, Liberia, Madagascar, Malawi, Mali, Mauritania, Mozambique, Niger, Nigeria, Rwanda, Sao Tome and Principe, Senegal, Sierra Leone, Somalia, Sudan, Togo, Uganda, United Republic of Tanzania, Zambia, Zimbabwe |
|  | ***Not included*** | | |
| ***Not included*** | Anguilla, British Virgin Islands, Cook Islands, Falkland Islands (Malvinas), French Guiana, Gibraltar, Guadeloupe, Holy See, Martinique, Montserrat, Nauru, Niue, Pitcairn, Réunion, Saint Helena, Saint Pierre et Miquelon, Tokelau, Turks and Caicos Islands, Tuvalu, Wallis and Futuna Islands, Western Sahara | | |

Note 1: Categories shown in Table 1 are based on the income categories published in the World Bank's “2003 World Development Indicators” Report (World Bank 2003). Countries are divided according to 2001 GNI per capita, calculated using the World Bank Atlas method. The groups are: low income, $745 or less; lower middle income, $746 - $2,975; upper middle income, $2,976 - $9,205; and high income, $9,206 or more.

Note 2: High income countries are a category in both classifications.
