# Supplementary material for: Projections of Global Mortality and Burden of Disease from 2002 to 2030
Source: PLoS Med. 2006 Nov 28;3(11):e442. doi: 10.1371/journal.pmed.0030442 (PMC1664601; doi:10.1371/journal.pmed.0030442)
Supplement: Table S2 — (184 KB DOC) [file pmed.0030442.st002.doc]

Table S2: GBD cause categories and ICD codes

| **GBD Cause Name** | **ICD-9 code** | **ICD-10 code** |
| --- | --- | --- |
| **All Causes** |  |  |
| **I. Communicable, maternal, perinatal and  nutritional conditions (a)** | 001-139, 243, 260- 269,*279.5*, 280-281, 285.9, 320-323, 381-382,460-465, 466, 480-487, 614-616, 630-676, 760-779 | A00-B99, G00-G04, N70-N73, J00-J06, J10-J18, J20-J22, H65-H66, O00-O99, P00-*P96*, E00-E02, E40-E46, E50, D50-D53, D64.9, E51-64 |
| **A. Infectious and parasitic diseases** | 001-139, *279.5*, 320-323, 614-616, 771.3 | A00-B99, G00, G03-G04, N70-N73 |
| 1. **Tuberculosis** | 010-018, 137 | A15-A19, B90 |
| 2. **Sexually transmitted diseases   excluding HIV** | 090-099, 614-616 | A50-A64, N70-N73 |
| 3. **HIV/AIDS** | 279.5 (=042-044) | B20-B24 |
| 4. **Diarrheal diseases** | 001, 002, 004, 006-009 | *A00, A01,A03, A04*, A06-A09 |
| 5. **Childhood-cluster diseases** | 032, 033, 037, 045,055, 138, 771.3 | A33-A37, A80, B05, B91 |
| a. Pertussis | 033 | A37 |
| b. Poliomyelitis | 045, 138 | A80,B91 |
| c. Diphtheria | 032 | A36 |
| d. Measles | 055 | B05 |
| e. Tetanus | 037, 771.3 | A33-A35 |
| 6. **Meningitis** | 036, 320-322 | A39, G00, G03 |
| 7. **Hepatitis B & C** | 070.2-070.9 | B16-B19 |
| 8. **Malaria** | 084 | B50-B54 |
| 9. **Tropical-cluster diseases** | 085, 086, 120, 125.0, 125.1, 125.3 | B55-B57, B65, B73, B74.0-B74.2 |
| a. Trypanosomiasis | 086.3, 086.4, 086.5, | B56 |
| b. Chagas disease | 086.0, 086.1, 086.2, 086.9 | B57 |
| c. Schistosomiasis | 120 | B65 |
| d. Leishmaniasis | 085 | B55 |
| e. Lymphatic filariasis | 125.0, 125.1 | B74.0-B74.2 |
| f. Onchocerciasis | 125.3 | B73 |
| 10. **Leprosy** | 030 | A30 |
| 11. **Dengue** | 061 | A90-A91 |
| 12. **Japanese encephalitis** | 062.0 | A83.0 |
| 13. **Trachoma** | 076 | A71 |
| 14. **Intestinal nematode infections** | 126-129 | *B76-B81* |
| a. Ascariasis | 127.0 | B77 |
| b. Trichuriasis | 127.3 | B79 |
| c. Hookworm disease  (Ancylostomiasis and necatoriasis) | 126 | B76 |
| **B. Respiratory infections** | 460-466, 480-487, 381-382 | J00-J06, J10-J18, J20-J22, H65-H66 |
| 1. Lower respiratory infections | 466, 480-487 | J10-J18, J20-J22 |
| 2. Upper respiratory infections | 460-465 | J00-J06 |
| 3. Otitis media | 381-382 | H65-H66 |
| **C. Maternal conditions** | 630-676 | O00-O99 |
| **D. Conditions arising during the perinatal   period** | 760-779 minus 771.3 | P00-*P96* |
| **E. Nutritional deficiencies** | 243, 260-269, 280-281, 285.9 | E00-E02, E40-E46, E50, D50-D53,D64.9, E51-E64 |
| 1. Protein-energy malnutrition | 260-263 | E40-E46 |
| 2. Iodine deficiency | 243 | E00-E02 |
| 3. Vitamin A deficiency | 264 | E50 |
| 4. Iron-deficiency anemia | 280, 285.9 | D50, D64.9 |
| Other nutritional disorders | 265-269, 281 | D51-D53, E51-E64 |

Table S2 (continued): GBD cause categories and ICD codes

| **GBD Cause Name** | **ICD-9 code** | **ICD-10 code** |
| --- | --- | --- |
| **II. Noncommunicable diseases (a)** | 140-242, 244-259, 270-279 (minus 279.5),282-285 (minus 285.9), 286-319, 324-380, 383-459, 470-478, 490-611, 617-629, 680-759 | C00-C97, D00-D48,D55-D64 (minus D 64.9) D65-D89, E03-E07, E10-E16, E20-E34, E65-E88, F01-F99, G06-G98, H00-H61, H68-H93, I00-I99, J30-J98, K00-K92, N00-N64, N75-N98, L00-L98, M00-M99, Q00-Q99 |
| **A. Malignant neoplasms** | 140-208 | C00-C97 |
| 1. Mouth and oropharynx cancers (b) | 140-149 | C00-C14 |
| 2. Oesophagus cancer (b) | 150 | C15 |
| 3. Stomach cancer (b) | 151 | C16 |
| 4. Colon and rectum cancers (b) | 153, 154 | C18-C21 |
| 5. Liver cancer | 155 | C22 |
| 6. Pancreas cancer | 157 | C25 |
| 7. Trachea, bronchus and lung cancers | 162 | C33-C34 |
| 8. Melanoma and other skin cancers (b) | 172-173 | C43-C44 |
| 9. Breast cancer (b) | 174, 175 | C50 |
| 10. Cervix uteri cancer (b) | 180 | C53 |
| 11. Corpus uteri cancer (b) | 179, 182 | C54-C55 |
| 12. Ovary cancer | 183 | C56 |
| 13. Prostate cancer (b) | 185 | C61 |
| 14. Bladder cancer (b) | 188 | C67 |
| 15. Lymphomas and multiple myeloma (b) | 200-203 | C81-C90, C96 |
| 16. Leukemia(b) | 204-208 | C91-C95 |
| **B. Other neoplasms** | 210-239 | D00-D48 |
| **C. Diabetes mellitus** | 250 | E10-E14 |
| **D. Endocrine disorders** | 240-242, 244-246, 251-259, 270-279 (minus 274, 279.5),282-285 (minus 285.9), *286*-289 | *D55-D64 (minus D64.9),D65-D89, E03-E07, E15-E16, E20-E34, E65-E88* |
| **E. Neuro-psychiatric conditions** | 290-319, 324-359 | F01-F99, G06-G98 |
| 1. Unipolar depressive disorders | 296.1, 311 | F32-F33 |
| 2. Bipolar affective disorder | 296 (minus 296.1) | F30-F31 |
| 3. Schizophrenia | 295 | F20-F29 |
| 4. Epilepsy | 345 | G40-G41 |
| 5. Alcohol use disorders | 291, 303, 305.0 | F10 |
| 6. Alzheimer and other dementias | 290, 330, 331 | F01, F03, G30-G31 |
| 7. Parkinson disease | 332 | G20-G21 |
| 8. Multiple sclerosis | 340 | G35 |
| 9. Drug use disorders | 304, 305.2-305.9 | F11-F16, F18-F19 |
| 10. Post-traumatic stress disorder | 308-309 | *F43.1* |
| 11. Obsessive-compulsive disorder | 300.3 | F42 |
| 12. Panic disorder | 300.2 | F40.0, F41.0 |
| 13. Insomnia (primary) | 307.4 | F51 |
| 14. Migraine | 346 | G43 |
| **F. Sense organ diseases** | 360-380, 383-389 | H00-H61, H68-H93 |
| 1. Glaucoma | 365 | H40 |
| 2. Cataracts | 366 | H25-H26 |
| 3. Vision disorders, age-related | 367.4 | H524 |
| 4. Hearing loss, adult onset | 389 | H90-H91 |
| **G. Cardiovascular diseases** | 390-459 | I00-I99 |
| 1. Rheumatic heart disease | 390-398 | I01-I09 |
| 2. Hypertensive heart disease | 401-405 | I10-I13 |
| 3. Ischemic heart disease I | 410-414 | I20-I25 |
| 4. Cerebrovascular disease | 430-438 | I60-I69 |
| 5. Inflammatory heart diseases | 420, 421, 422, 425 | I30-I33, I38, I40, I42 |
| **H. Respiratory diseases** | 470-478, 490-519 | J30-J98 |
| 1. Chronic obstructive pulmonary disease | 490-492, 495-496 | J40-J44 |
| 2. Asthma | 493 | J45-J46 |

Table S2 (continued): GBD cause categories and ICD codes

| **GBD Cause Name** | **ICD-9 code** | **ICD-10 code** |
| --- | --- | --- |
| **I. Digestive diseases** | 530-579 | K20-*K92* |
| 1. Peptic ulcer disease | 531-533 | K25-K27 |
| 2. Cirrhosis of the liver | 571 | K70, K74 |
| 3. Appendicitis | 540-543 | K35-K37 |
| **J. Genito-urinary diseases** | 580-611, 617-629 | N00-N64, N75-N98 |
| 1. Nephritis and nephrosis | 580-589 | N00-N19 |
| 2. Benign prostatic hypertrophy | 600 | N40 |
| **K. Skin diseases** | 680-709 | L00-L98 |
| **L. Musculoskeletal diseases** | 710-739, 274 | M00-M99 |
| 1. Rheumatoid arthritis | 714 | M05-M06 |
| 2. Osteoarthritis | 715 | M15-M19 |
| **M. Congenital anomalies** | 740-759 | Q00-Q99 |
| **N. Oral conditions** | 520-529 | K00-K14 |
| **III. Injuries** | E800-999 | V01-Y89 |
| **A. Unintentional injuries (d)** | E800-949 | V01-X59, Y40-Y86, Y88, Y89 |
| 1. Road traffic accidents | E810-819, E826-829, E929.0 | (e) |
| 2. Poisonings | E850-869 | X40-X49 |
| 3. Falls | E880-888 | W00-W19 |
| 4. Fires | E890-899 | X00-X09 |
| 5. Drownings | E910 | W65-W74 |
| 6. Other unintentional injuries | E800-E807, E820-E848, E870-E879, E900-E909, E911-E949 | *Rest of V, W20-*W64, W75-W99, X10-X39, X50-X59, Y40-Y86, Y88,Y89 |
| **B. Intentional injuries (d)** | E950-978, 990-999 | X60-Y09, Y35-Y36, Y870, Y871 |
| 1. Self-inflicted injuries | E950-959 | X60-X84, Y870 |
| 2. Violence | E960-969 | X85-Y09, Y871 |
| 3. War | E990-999 | Y36 |

(a) Deaths coded to “Symptoms, signs and ill-defined conditions” (780-799 in ICD-9 and R00-R99 in ICD-10) are distributed proportionately to all causes within Group I and Group II.

(b) Cancer deaths coded to ICD categories for malignant neoplasms of other and unspecified sites including those whose point of origin cannot be determined, and secondary and unspecified neoplasms (ICD-10 C76, C80, C97 or ICD-9 195, 199) were redistributed pro-rata across the footnoted malignant neoplasm categories within each age–sex group, so that the category ‘Other malignant neoplasms’ includes only malignant neoplasms of other specified sites (53).

(c) Ischemic heart disease deaths may be miscoded to a number of so-called cardiovascular “garbage” codes. These include heart failure, ventricular dysrhythmias, generalized atherosclerosis and ill-defined descriptions and complications of heart disease. Proportions of deaths coded to these causes were redistributed to ischemic heart disease as described in(23). Relevant ICD-9 codes are 427.1, 427.4, 427.5, 428, 429.0, 429.1, 429.2, 429.9, 440.9, and relevant ICD-10 codes are I47.2, I49.0, I46, I50, I51.4, I51.5, I51.6, I51.9, I70.9.

(d) Injury deaths where the intent is not determined (E980-989 of ICD-9 and Y10-Y34, Y872 in ICD-10) are distributed proportionately to all causes below the Group level for injuries.

(e) For countries with 3-digit ICD10 data,for Road traffic accidents use: V01-V04, V06, V09-V80, V87, V89, V99. For countries with 4-digit ICD10 data,for Road traffic accidents use:

V01.1-V01.9, V02.1-V02.9, V03.1-V03.9, V04.1-V04.9, V06.1-V06.9, V09.2, V09.3, V10.4-V10.9, V11.4-V11.9, V12.3-V12.9, V13.3-V13.9, V14.3-V14.9, V15.4-V15.9, V16.4-V16.9, V17.4-V17.9, V18.4-V18.9, V19.4-V19.6, V20.3-V20.9, V21.3-V21.9, V22.3-V22.9, V23.3-V23.9, V24.3-V24.9, V25.3-V25.9, V26.3-V26.9, V27.3-V27.9, V28.3-V28.9, V29.4-V29.9, V30.4.V30.9, V31.4-V31.9, V32.4-V32.9, V33.4-V33.9, V34.4-V34.9, V35.4-V35.9, V36.4-V36.9, V37.4-V37.9, V38.4-V38.9, V39.4-V39.9, V40.4-V40.9, V41.4-V41.9, V42.4-V42.9, V43.4-V43.9, V44.4-V44.9, V45.4-V45.9, V46.4-V46.9, V47.4-V47.9, V48.4-V48.9, V49.4-V49.9, V50.4-V50.9, V51.4-V51.9, V52.4-V52.9, V53.4-V53.9, V54.4-V54.9, V55.4-V55.9, V56.4-V56.9, V57.4-V57.9, V58.4-V58.9, V59.4-V59.9, V60.4-V60.9, V61.4-V61.9, V62.4-V62.9, V63.4-V63.9, V64.4-V64.9, V65.4-V65.9, V66.4-V66.9, V67.4-V67.9, V68.4-V68.9, V69.4-V69.9, V70.4-V70.9, V71.4-V71.9, V72.4-V72.9, V73.4-V73.9, V74.4-V74.9, V75.4-V75.9, V76.4-V76.9, V77.4-V77.9, V78.4-V78.9, V79.4-V79.9, V80.3-V80.5, V81.1, V82.1, V83.0-V83.3, V84.0-V84.3, V85.0-V85.3, V86.0-V86.3, V87.0-V87.8, V89.2, V89.9, V99, Y850.
