# Supplementary material for: Projections of Global Mortality and Burden of Disease from 2002 to 2030
Source: PLoS Med. 2006 Nov 28;3(11):e442. doi: 10.1371/journal.pmed.0030442 (PMC1664601; doi:10.1371/journal.pmed.0030442)
Supplement: Table S3 — (334 KB DOC) [file pmed.0030442.st003.doc]

Table S3: Parsimonious regression equations for nine major cause-clusters based on the full country panel dataset, 1950-2002.

| **Cause-cluster** | **Sex** | **Age group** | **Regression coefficients** | | | | | | |
| --- | --- | --- | --- | --- | --- | --- | --- | --- | --- |
|  |  |  | **Constant** | **lnY** | **lnHC** | **(lnY)^2^** | **Year** | **lnSI** | **R^2^ (%)** |
| *Group I* |  |  |  |  |  |  |  |  |  |
|  | Male | 0-4 | 16.359 | -0.754 | -0.947 |  | -0.025 |  | 79 |
|  |  | 5-14 | 15.126 | -0.917 | -1.814 |  | -0.020 |  | 75 |
|  |  | 15-29 | 13.220 | -0.823 | -1.429 |  | -0.012 |  | 72 |
|  |  | 30-44 | 13.950 | -0.898 | -1.124 |  | -0.010 |  | 69 |
|  |  | 45-59 | 13.759 | -0.747 | -0.960 |  | -0.016 |  | 69 |
|  |  | 60-69 | 12.937 | -0.492 | -1.064 |  | -0.019 |  | 65 |
|  |  | 70+ | 10.176 | 0.000 | -1.222 |  | -0.017 |  | 39 |
|  | Female | 0-4 | 16.282 | -0.786 | -0.953 |  | -0.024 |  | 78 |
|  |  | 5-14 | 14.763 | -0.913 | -1.780 |  | -0.019 |  | 74 |
|  |  | 15-29 | 14.645 | -0.887 | -1.502 |  | -0.021 |  | 74 |
|  |  | 30-44 | 15.137 | -0.865 | -1.494 |  | -0.024 |  | 74 |
|  |  | 45-59 | 13.056 | -0.646 | -1.338 |  | -0.020 |  | 71 |
|  |  | 60-69 | 12.347 | -0.417 | -1.406 |  | -0.022 |  | 65 |
|  |  | 70+ | 9.240 | 0.148 | -1.535 |  | -0.019 |  | 42 |
| *Malignant neoplasms* | | |  |  |  |  |  |  |  |
|  | Male | 0-4 | -12.871 | 3.630 | 0.396 | -0.217 | -0.011 |  | 26 |
|  |  | 5-14 | -7.702 | 2.408 | 0.064 | -0.142 | -0.009 |  | 23 |
|  |  | 15-29 | 0.815 | 0.564 |  | -0.037 | -0.007 |  | 16 |
|  |  | 30-44 | 4.791 | -0.089 | -0.071 |  | -0.006 | 0.096 | 24 |
|  |  | 45-59 | 5.452 | -0.056 | -0.143 |  | -0.004 | 0.196 | 31 |
|  |  | 60-69 | 5.998 |  | -0.091 |  | -0.006 | 0.175 | 31 |
|  |  | 70+ | 5.530 | 0.195 | -0.184 |  | -0.007 | 0.133 | 37 |
|  | Female | 0-4 | -14.155 | 3.894 | 0.325 | -0.230 | -0.011 |  | 26 |
|  |  | 5-14 | -8.115 | 2.435 |  | -0.143 | -0.008 |  | 19 |
|  |  | 15-29 | -0.384 | 0.926 | -0.150 | -0.063 | -0.006 |  | 34 |
|  |  | 30-44 | 1.503 | 0.871 | -0.131 | -0.057 | -0.008 | 0.013 | 30 |
|  |  | 45-59 | 3.394 | 0.639 | -0.057 | -0.038 | -0.010 | 0.037 | 21 |
|  |  | 60-69 | 3.920 | 0.621 | -0.046 | -0.033 | -0.010 | 0.034 | 19 |
|  |  | 70+ | 6.161 | 0.194 | -0.208 |  | -0.012 | 0.062 | 29 |
| *Cardiovascular diseases* | |  |  |  |  |  |  |  |  |
|  | Male | 0-4 | -4.656 | 2.071 | -0.798 | -0.140 | 0.012 |  | 31 |
|  |  | 5-14 | 7.875 | -0.480 | -1.040 |  | -0.009 |  | 62 |
|  |  | 15-29 | 5.697 | 0.000 | -0.593 | -0.028 |  |  | 60 |
|  |  | 30-44 | -0.898 | 1.597 | -0.074 | -0.115 |  | 0.087 | 38 |
|  |  | 45-59 | 3.613 | 0.821 |  | -0.066 | -0.005 | 0.172 | 34 |
|  |  | 60-69 | 8.312 | 0.000 | 0.109 | -0.019 | -0.008 | 0.158 | 33 |
|  |  | 70+ | 6.924 | 0.545 | 0.235 | -0.046 | -0.005 | 0.072 | 23 |
|  | Female | 0-4 | -5.246 | 2.138 | -0.677 | -0.146 | 0.013 |  | 31 |
|  |  | 5-14 | 8.281 | -0.525 | -1.007 |  | -0.011 |  | 65 |
|  |  | 15-29 | 9.397 | -0.582 | -0.821 |  | -0.009 |  | 72 |
|  |  | 30-44 | 1.721 | 1.315 | -0.376 | -0.108 | -0.009 | 0.017 | 67 |
|  |  | 45-59 | -2.062 | 2.409 |  | -0.170 | -0.010 | 0.033 | 63 |
|  |  | 60-69 | 1.117 | 1.882 | 0.186 | -0.135 | -0.011 |  | 58 |
|  |  | 70+ | 7.171 | 0.531 | 0.204 | -0.045 | -0.002 | -0.069 | 36 |
| *Digestive diseases* | | |  |  |  |  |  |  |  |
|  | Male | 0-4 | -1.562 | 2.356 | -0.825 | -0.173 | -0.022 |  | 56 |
|  |  | 5-14 | 0.829 | 1.640 | -1.247 | -0.140 | -0.018 |  | 73 |
|  |  | 15-29 | 0.371 | 1.423 | -1.188 | -0.118 |  |  | 68 |
|  |  | 30-44 | -1.653 | 1.726 | -0.923 | -0.124 | 0.013 |  | 44 |
|  |  | 45-59 | -0.676 | 1.669 | -0.767 | -0.115 | 0.010 |  | 41 |
|  |  | 60-69 | 1.608 | 1.355 | -0.720 | -0.095 | 0.004 |  | 47 |
|  |  | 70+ | 8.429 | -0.121 | -0.792 |  |  |  | 42 |

Table S3: (continued):Parsimonious regression equations for nine major cause-clusters based on the full country panel dataset, 1950-2002.

| **Cause-cluster** | **Sex** | | | **Age group** | | **Regression coefficients** | | | | | | | | | | | | |
| --- | --- | --- | --- | --- | --- | --- | --- | --- | --- | --- | --- | --- | --- | --- | --- | --- | --- | --- |
|  |  |  |  |  |  | **Constant** | | **lnY** | | **lnHC** | | **(lnY)^2^** | | **Year** | | **lnSI** | | **R^2^ (%)** |
| *Digestive diseases* | | | |  | |  | |  | |  | |  | |  | |  | |  |
|  | Female | | | 0-4 | | -2.754 | | 2.491 | | -0.843 | | -0.181 | | -0.018 | |  | | 53 |
|  |  | | | 5-14 | | 0.463 | | 1.569 | | -1.173 | | -0.134 | | -0.017 | |  | | 65 |
|  |  | | | 15-29 | | -1.869 | | 1.941 | | -1.111 | | -0.148 | | -0.007 | |  | | 72 |
|  |  | | | 30-44 | | 7.554 | | -0.381 | | -1.028 | |  | |  | |  | | 51 |
|  |  | | | 45-59 | | 3.645 | | 0.698 | | -0.785 | | -0.062 | |  | |  | | 47 |
|  |  | | | 60-69 | | 3.447 | | 0.919 | | -0.770 | | -0.073 | |  | |  | | 53 |
|  |  | | | 70+ | | 12.834 | | -1.289 | | -0.806 | | 0.071 | |  | |  | | 35 |
| *Respiratory diseases* | | | | | |  | |  | |  | |  | |  | |  | |  |
|  | Male | | | 0-4 | | 10.369 | | -0.504 | | -1.896 | |  | |  | |  | | 53 |
|  |  | | | 5-14 | | 7.044 | | -0.519 | | -1.190 | |  | |  | |  | | 55 |
|  |  | | | 15-29 | | 8.179 | | -1.425 | | -1.069 | | 0.071 | | 0.019 | |  | | 45 |
|  |  | | | 30-44 | | -4.721 | | 2.028 | | -0.778 | | -0.141 | | 0.013 | | 0.047 | | 50 |
|  |  | | | 45-59 | | -0.140 | | 1.126 | | -0.446 | | -0.087 | | 0.007 | | 0.209 | | 30 |
|  |  | | | 60-69 | | 5.801 | | -0.249 | | -0.272 | |  | | 0.011 | | 0.201 | | 20 |
|  |  | | | 70+ | | 6.354 | | -0.230 | | -0.298 | |  | | 0.020 | | 0.175 | | 30 |
|  | Female | | | 0-4 | | -0.578 | | 2.026 | | -1.566 | | -0.156 | |  | |  | | 52 |
|  |  | | | 5-14 | | 4.380 | | 0.153 | | -1.166 | | -0.049 | | 0.004 | |  | | 52 |
|  |  | | | 15-29 | | 4.219 | | -0.367 | | -0.869 | |  | | 0.014 | |  | | 44 |
|  |  | | | 30-44 | | 4.848 | | -0.384 | | -0.765 | |  | | 0.016 | |  | | 43 |
|  |  | | | 45-59 | | 5.756 | | -0.387 | | -0.678 | |  | | 0.018 | | 0.141 | | 44 |
|  |  | | | 60-69 | | 6.714 | | -0.357 | | -0.747 | |  | | 0.018 | | 0.151 | | 47 |
|  |  | | | 70+ | | 10.369 | | -0.504 | | -1.896 | |  | |  | |  | | 53 |
| *Other Group II* | | | | | |  | |  | |  | |  | |  | |  | |  |
|  | Male | | | 0-4 | | 9.383 | | -0.286 | | -0.558 | |  | | -0.010 | |  | | 57 |
|  |  | | | 5-14 | | 7.815 | | -0.292 | | -0.677 | |  | | -0.012 | |  | | 71 |
|  |  | | | 15-29 | | 7.641 | | -0.296 | | -0.670 | |  | |  | |  | | 34 |
|  |  | | | 30-44 | | -0.369 | | 1.648 | | -0.324 | | -0.112 | | 0.004 | |  | | 46 |
|  |  | | | 45-59 | | 0.638 | | 1.607 | |  | | -0.104 | | 0.000 | |  | | 24 |
|  |  | | | 60-69 | | 4.913 | | 0.851 | | 0.077 | | -0.057 | | -0.003 | |  | | 16 |
|  |  | | | 70+ | | 9.983 | | -0.101 | |  | |  | | -0.001 | |  | | 11 |
|  | Female | | | 0-4 | | 9.207 | | -0.299 | | -0.502 | |  | | -0.010 | |  | | 55 |
|  |  | | | 5-14 | | 7.799 | | -0.308 | | -0.734 | |  | | -0.011 | |  | | 69 |
|  |  | | | 15-29 | | 8.613 | | -0.378 | | -0.719 | |  | | -0.006 | |  | | 71 |
|  |  | | | 30-44 | | 4.478 | | 0.678 | | -0.479 | | -0.056 | | -0.006 | |  | | 62 |
|  |  | | | 45-59 | | 4.857 | | 0.751 | | -0.209 | | -0.057 | | -0.006 | |  | | 49 |
|  |  | | | 60-69 | | 6.625 | | 0.586 | | -0.139 | | -0.048 | | -0.007 | |  | | 48 |
|  |  | | | 70+ | | 10.587 | | -0.161 | | -0.121 | | 0.000 | | -0.002 | |  | | 30 |
| *Road traffic accidents* | | | | |  | |  | |  | |  | |  | |  | |  | |
|  | | Male | 0-4 | | -21.339 | | 5.341 | | 0.294 | | -0.306 | | -0.006 | |  | | 15 | |
|  | |  | 5-14 | | -17.511 | | 4.635 | | -0.156 | | -0.262 | | -0.007 | |  | | 18 | |
|  | |  | 15-29 | | -17.821 | | 4.580 | | 0.000 | | -0.247 | | 0.002 | |  | | 19 | |
|  | |  | 30-44 | | -25.046 | | 6.579 | | -0.297 | | -0.384 | | 0.012 | |  | | 26 | |
|  | |  | 45-59 | | -28.264 | | 7.361 | | -0.285 | | -0.428 | | 0.009 | |  | | 29 | |
|  | |  | 60-69 | | -24.713 | | 6.630 | | -0.383 | | -0.381 | | 0.004 | |  | | 24 | |
|  | |  | 70+ | | -17.562 | | 4.940 | | -0.348 | | -0.277 | | 0.003 | |  | | 13 | |
|  | | Female | 0-4 | | -20.599 | | 5.054 | | 0.278 | | -0.286 | | -0.006 | |  | | 14 | |
|  | |  | 5-14 | | -13.515 | | 3.493 | | -0.082 | | -0.196 | | -0.003 | |  | | 8 | |
|  | |  | 15-29 | | -11.870 | | 2.708 | | 0.078 | | -0.139 | | 0.008 | |  | | 18 | |
|  | |  | 30-44 | | -15.493 | | 3.808 | | -0.134 | | -0.217 | | 0.012 | |  | | 12 | |
|  | |  | 45-59 | | -15.914 | | 4.099 | | -0.167 | | -0.235 | | 0.007 | |  | | 11 | |
|  | |  | 60-69 | | -15.160 | | 4.060 | | -0.247 | | -0.229 | | 0.003 | |  | | 9 | |
|  | |  | 70+ | | -15.311 | | 4.196 | | -0.149 | | -0.236 | |  | |  | | 7 | |

Table S3: (continued):Parsimonious regression equations for nine major cause-clusters based on the full country panel dataset, 1950-2002.

| **Cause-cluster** | **Sex** | **Age group** | **Regression coefficients** | | | | | | |
| --- | --- | --- | --- | --- | --- | --- | --- | --- | --- |
|  |  |  | **Constant** | **lnY** | **lnHC** | **(lnY)^2^** | **Year** | **lnSI** | **R^2^ (%)** |
| *Other unintentional injuries* | | |  |  |  |  |  |  |  |
|  | Male | 0-4 | -8.376 | 3.283 | 0.373 | -0.221 | -0.008 |  | 36 |
|  |  | 5-14 | -3.043 | 2.160 | -0.189 | -0.156 | -0.012 |  | 58 |
|  |  | 15-29 | -5.284 | 2.640 | -0.270 | -0.177 | -0.003 |  | 42 |
|  |  | 30-44 | -8.333 | 3.238 | -0.109 | -0.215 | 0.006 |  | 36 |
|  |  | 45-59 | -8.487 | 3.247 | 0.000 | -0.214 | 0.006 |  | 31 |
|  |  | 60-69 | -5.179 | 2.530 | -0.220 | -0.165 | 0.004 |  | 29 |
|  |  | 70+ | 1.000 |  |  |  |  |  | 5 |
|  | Female | 0-4 | -6.429 | 2.817 | 0.330 | -0.198 | -0.006 |  | 38 |
|  |  | 5-14 | 5.552 | 0.000 | -0.452 | -0.034 |  |  | 57 |
|  |  | 15-29 | 4.741 | 0.000 | -0.520 | -0.031 | 0.005 |  | 50 |
|  |  | 30-44 | -1.725 | 1.341 | -0.237 | -0.108 | 0.010 |  | 40 |
|  |  | 45-59 | -2.851 | 1.600 | -0.110 | -0.118 | 0.008 |  | 31 |
|  |  | 60-69 | 1.000 |  |  |  |  |  | 29 |
|  |  | 70+ | 1.000 |  |  |  |  |  | 16 |
| *Intentional injuries* | | |  |  |  |  |  |  |  |
|  | Male | 0-4 | 1.000 |  |  |  |  |  | 33 |
|  |  | 5-14 | -3.841 | 1.255 | -0.403 | -0.095 | 0.020 |  | 33 |
|  |  | 15-29 | 9.706 | -1.369 | -0.639 | 0.065 | 0.022 |  | 22 |
|  |  | 30-44 | 5.274 | -0.267 | -0.458 |  | 0.019 |  | 19 |
|  |  | 45-59 | 4.408 | 0.000 | -0.196 | -0.013 | 0.009 |  | 10 |
|  |  | 60-69 | 1.000 |  |  |  |  |  | 9 |
|  |  | 70+ | 1.000 |  |  |  |  |  | 5 |
|  | Female | 0-4 | 1.000 |  |  |  |  |  | 5 |
|  |  | 5-14 | 2.887 | -0.396 | -0.529 | 0.000 | 0.021 |  | 40 |
|  |  | 15-29 | 13.165 | -2.313 | -0.172 | 0.118 | 0.005 |  | 13 |
|  |  | 30-44 | 10.345 | -1.856 | 0.229 | 0.101 |  |  | 3 |
|  |  | 45-59 | 1.000 |  |  |  |  |  | 8 |
|  |  | 60-69 | 1.000 |  |  |  |  |  | 6 |
|  |  | 70+ | 1.000 |  |  |  |  |  | 7 |
