# Supplementary material for: Projections of Global Mortality and Burden of Disease from 2002 to 2030
Source: PLoS Med. 2006 Nov 28;3(11):e442. doi: 10.1371/journal.pmed.0030442 (PMC1664601; doi:10.1371/journal.pmed.0030442)
Supplement: Table S4 — (335 KB DOC) [file pmed.0030442.st004.doc]

Table S4: Parsimonious regression equations for nine major cause-clusters based on the low income country panel dataset, 1950-2002.

| **Cause-cluster** | **Sex** | **Age group** | **Regression coefficients** | | | | | | |
| --- | --- | --- | --- | --- | --- | --- | --- | --- | --- |
|  |  |  | **Constant** | **lnY** | **lnHC** | **(lnY)^2^** | **Year** | **lnSI** | **R^2^ (%)** |
| *Group I* |  |  |  |  |  |  |  |  |  |
|  | Male | 0-4 | 14.463 | -0.543 | -1.113 |  | -0.019 |  | 65 |
|  |  | 5-14 | 14.610 | -0.849 | -1.973 |  | -0.016 |  | 65 |
|  |  | 15-29 | 12.697 | -0.749 | -1.472 |  | -0.012 |  | 58 |
|  |  | 30-44 | 12.263 | -0.691 | -1.149 |  | -0.010 |  | 49 |
|  |  | 45-59 | 12.197 | -0.564 | -0.991 |  | -0.014 |  | 50 |
|  |  | 60-69 | 12.670 | -0.449 | -1.095 |  | -0.020 |  | 54 |
|  |  | 70+ | 12.315 | -0.209 | -1.290 |  | -0.022 |  | 44 |
|  | Female | 0-4 | 14.241 | -0.557 | -1.148 |  | -0.017 |  | 66 |
|  |  | 5-14 | 14.000 | -0.817 | -1.970 |  | -0.015 |  | 66 |
|  |  | 15-29 | 13.318 | -0.713 | -1.662 |  | -0.019 |  | 63 |
|  |  | 30-44 | 13.731 | -0.681 | -1.680 |  | -0.022 |  | 64 |
|  |  | 45-59 | 12.598 | -0.576 | -1.531 |  | -0.017 |  | 63 |
|  |  | 60-69 | 12.771 | -0.438 | -1.552 |  | -0.022 |  | 61 |
|  |  | 70+ | 11.936 | -0.117 | -1.624 |  | -0.024 |  | 48 |
| *Malignant neoplasms* | | |  |  |  |  |  |  |  |
|  | Male | 0-4 | -4.886 | 1.589 | 0.392 | -0.092 | -0.007 |  | 14 |
|  |  | 5-14 | -4.608 | 1.612 |  | -0.094 | -0.006 |  | 7 |
|  |  | 15-29 | 3.296 | -0.061 |  |  | -0.006 |  | 6 |
|  |  | 30-44 | 5.175 | -0.128 | -0.081 |  | -0.007 | 0.109 | 22 |
|  |  | 45-59 | 5.819 | -0.096 | -0.102 |  | -0.006 | 0.201 | 35 |
|  |  | 60-69 | 6.055 |  | -0.067 |  | -0.007 | 0.178 | 34 |
|  |  | 70+ | 2.000 | 1.081 | -0.165 | -0.055 | -0.009 | 0.146 | 31 |
|  | Female | 0-4 | -5.658 | 1.724 | 0.322 | -0.097 | -0.007 |  | 15 |
|  |  | 5-14 | -4.318 | 1.452 | -0.059 | -0.083 | -0.004 |  | 6 |
|  |  | 15-29 | -3.826 | 1.776 | -0.181 | -0.116 | -0.005 |  | 18 |
|  |  | 30-44 | -2.608 | 1.871 | -0.145 | -0.119 | -0.007 |  | 17 |
|  |  | 45-59 | -2.636 | 2.132 | -0.047 | -0.131 | -0.010 | 0.021 | 17 |
|  |  | 60-69 | -3.453 | 2.459 |  | -0.147 | -0.011 | 0.019 | 19 |
|  |  | 70+ | -1.005 | 1.999 | -0.190 | -0.112 | -0.014 | 0.076 | 30 |
| *Cardiovascular diseases* | |  |  |  |  |  |  |  |  |
|  | Male | 0-4 | -15.604 | 4.774 | -0.835 | -0.306 | 0.013 |  | 22 |
|  |  | 5-14 | 6.284 |  | -1.070 | -0.032 | -0.010 |  | 54 |
|  |  | 15-29 | -2.425 | 1.969 | -0.592 | -0.147 |  |  | 41 |
|  |  | 30-44 | 6.385 | -0.260 | -0.221 |  | 0.006 | 0.099 | 17 |
|  |  | 45-59 | 7.641 | -0.248 | -0.069 |  | 0.000 | 0.164 | 20 |
|  |  | 60-69 | 8.906 | -0.256 |  |  | -0.003 | 0.152 | 19 |
|  |  | 70+ | 9.772 | -0.207 | 0.192 |  | -0.002 | 0.073 | 12 |
|  | Female | 0-4 | -14.572 | 4.442 | -0.702 | -0.287 | 0.013 |  | 21 |
|  |  | 5-14 | 8.531 | -0.538 | -1.044 |  | -0.012 |  | 55 |
|  |  | 15-29 | -2.952 | 2.454 | -0.900 | -0.184 | -0.009 |  | 61 |
|  |  | 30-44 | -3.924 | 2.617 | -0.500 | -0.185 | -0.004 |  | 48 |
|  |  | 45-59 | 0.084 | 1.772 | -0.079 | -0.129 | -0.004 |  | 26 |
|  |  | 60-69 | 8.495 |  | 0.077 | -0.019 | -0.006 |  | 20 |
|  |  | 70+ | 9.261 |  | 0.190 | -0.013 |  | -0.071 | 16 |
| *Digestive diseases* | | |  |  |  |  |  |  |  |
|  | Male | 0-4 | -10.378 | 4.523 | -0.888 | -0.307 | -0.020 |  | 41 |
|  |  | 5-14 | -8.357 | 3.933 | -1.295 | -0.282 | -0.018 |  | 63 |
|  |  | 15-29 | -4.100 | 2.537 | -1.208 | -0.186 |  |  | 55 |
|  |  | 30-44 | -0.693 | 1.434 | -0.956 | -0.105 | 0.016 |  | 32 |
|  |  | 45-59 | 6.685 | -0.201 | -0.786 |  | 0.013 |  | 29 |
|  |  | 60-69 | 1.403 | 1.376 | -0.712 | -0.097 | 0.006 |  | 32 |
|  |  | 70+ | 8.806 | -0.162 | -0.817 |  |  |  | 37 |

Table S4 (continued): Parsimonious regression equations for nine major cause-clusters based on the low income country panel dataset, 1950-2002.

| **Cause-cluster** | **Sex** | **Age group** | **Regression coefficients** | | | | | | |
| --- | --- | --- | --- | --- | --- | --- | --- | --- | --- |
|  |  |  | **Constant** | **lnY** | **lnHC** | **(lnY)^2^** | **Year** | **lnSI** | **R^2^ (%)** |
| *Digestive diseases* | | |  |  |  |  |  |  |  |
|  | Female | 0-4 | -8.986 | 4.002 | -0.929 | -0.274 | -0.015 |  | 38 |
|  |  | 5-14 | -8.933 | 3.929 | -1.216 | -0.280 | -0.017 |  | 60 |
|  |  | 15-29 | -8.479 | 3.552 | -1.205 | -0.246 | -0.004 |  | 56 |
|  |  | 30-44 | -1.514 | 1.796 | -1.101 | -0.132 | 0.003 |  | 45 |
|  |  | 45-59 | -4.351 | 2.604 | -0.867 | -0.178 | 0.005 |  | 37 |
|  |  | 60-69 | -3.199 | 2.575 | -0.797 | -0.175 |  |  | 40 |
|  |  | 70+ | 8.431 | -0.154 | -0.871 |  |  |  | 35 |
| *Respiratory diseases* | | |  |  |  |  |  |  |  |
|  | Male | 0-4 | 8.275 |  | -2.010 | -0.027 |  |  | 46 |
|  |  | 5-14 | -10.871 | 4.119 | -1.246 | -0.296 |  |  | 53 |
|  |  | 15-29 | 4.311 | -0.351 | -1.110 |  | 0.019 |  | 46 |
|  |  | 30-44 | 4.905 | -0.312 | -0.812 |  | 0.014 | 0.031 | 33 |
|  |  | 45-59 | 5.695 | -0.269 | -0.571 |  | 0.007 | 0.199 | 16 |
|  |  | 60-69 | 14.297 | -2.238 | -0.431 | 0.121 | 0.009 | 0.207 | 16 |
|  |  | 70+ | 16.495 | -2.555 | -0.398 | 0.142 | 0.015 | 0.134 | 20 |
|  | Female | 0-4 | 7.940 |  | -1.724 | -0.033 |  |  | 43 |
|  |  | 5-14 | -12.653 | 4.656 | -1.213 | -0.337 |  |  | 58 |
|  |  | 15-29 | 5.651 | -0.508 | -1.037 |  | 0.015 |  | 46 |
|  |  | 30-44 | 12.313 | -2.193 | -0.971 | 0.110 | 0.019 | -0.029 | 45 |
|  |  | 45-59 | 11.488 | -1.745 | -0.841 | 0.085 | 0.017 | 0.037 | 40 |
|  |  | 60-69 | 7.227 | -0.357 | -0.879 |  | 0.016 | 0.073 | 41 |
|  |  | 70+ | 19.552 | -3.093 | -0.769 | 0.169 | 0.019 |  | 34 |
| *Other Group II* | | |  |  |  |  |  |  |  |
|  | Male | 0-4 | 8.903 | -0.239 | -0.658 |  | -0.007 |  | 43 |
|  |  | 5-14 | 7.933 | -0.309 | -0.748 |  | -0.010 |  | 61 |
|  |  | 15-29 | 7.454 | -0.273 | -0.727 |  | 0.001 |  | 51 |
|  |  | 30-44 | 6.777 | -0.168 | -0.397 |  | 0.008 |  | 23 |
|  |  | 45-59 | 7.398 | -0.101 |  |  | 0.003 |  | 4 |
|  |  | 60-69 | 8.141 | 0.023 | 0.028 | -0.007 | 0.000 |  | 2 |
|  |  | 70+ | 9.798 | -0.084 | -0.034 |  |  |  | 4 |
|  | Female | 0-4 | 8.517 | -0.229 | -0.617 |  | -0.007 |  | 41 |
|  |  | 5-14 | 7.781 | -0.307 | -0.835 |  | -0.009 |  | 60 |
|  |  | 15-29 | 8.228 | -0.331 | -0.840 |  | -0.004 |  | 60 |
|  |  | 30-44 | 1.319 | 1.428 | -0.582 | -0.100 | -0.003 |  | 49 |
|  |  | 45-59 | 1.551 | 1.536 | -0.299 | -0.104 | -0.003 |  | 31 |
|  |  | 60-69 | 5.017 | 0.958 | -0.218 | -0.069 | -0.004 |  | 29 |
|  |  | 70+ | 10.506 | -0.144 | -0.245 |  |  |  | 17 |
| *Road traffic accidents* | | |  |  |  |  |  |  |  |
|  | Male | 0-4 | -1.600 | 0.353 | 0.124 |  | 0.004 |  | 13 |
|  |  | 5-14 | -13.564 | 3.608 | -0.276 | -0.200 |  |  | 13 |
|  |  | 15-29 | -10.318 | 2.673 | -0.170 | -0.130 | 0.009 |  | 22 |
|  |  | 30-44 | -17.181 | 4.550 | -0.423 | -0.259 | 0.019 |  | 25 |
|  |  | 45-59 | -15.010 | 3.979 | -0.418 | -0.220 | 0.018 |  | 26 |
|  |  | 60-69 | -7.175 | 2.174 | -0.540 | -0.108 | 0.014 |  | 22 |
|  |  | 70+ | 0.464 | 0.403 | -0.540 |  | 0.013 |  | 16 |
|  | Female | 0-4 | -2.122 | 0.376 | 0.117 |  | 0.005 |  | 15 |
|  |  | 5-14 | -7.536 | 1.945 | -0.243 | -0.103 | 0.005 |  | 8 |
|  |  | 15-29 | -2.918 | 0.419 | -0.123 |  | 0.018 |  | 23 |
|  |  | 30-44 | -1.470 | 0.247 | -0.366 |  | 0.023 |  | 27 |
|  |  | 45-59 | -0.624 | 0.236 | -0.409 |  | 0.019 |  | 21 |
|  |  | 60-69 | -0.059 | 0.276 | -0.484 |  | 0.014 |  | 15 |
|  |  | 70+ | 0.511 | 0.264 | -0.346 |  | 0.011 |  | 9 |

Table S4 (continued): Parsimonious regression equations for nine major cause-clusters based on the low income country panel dataset, 1950-2002.

| **Cause-cluster** | **Sex** | **Age group** | **Regression coefficients** | | | | | | |
| --- | --- | --- | --- | --- | --- | --- | --- | --- | --- |
|  |  |  | **Constant** | **lnY** | **lnHC** | **(lnY)^2^** | **Year** | **lnSI** | **R^2^ (%)** |
| *Other unintentional injuries* | | |  |  |  |  |  |  |  |
|  | Male | 0-4 | 5.553 | -0.293 | 0.257 |  |  |  | 7 |
|  |  | 5-14 | 6.628 | -0.335 | -0.332 |  | -0.006 |  | 28 |
|  |  | 15-29 | -1.357 | 1.161 | -0.341 | -0.111 | 0.000 |  | 15 |
|  |  | 30-44 | -7.251 | 2.882 | -0.187 | -0.191 | 0.011 |  | 14 |
|  |  | 45-59 | -8.878 | 3.250 | 0.000 | -0.213 | 0.010 |  | 12 |
|  |  | 60-69 | -4.818 | 2.352 | -0.251 | -0.152 | 0.008 |  | 10 |
|  |  | 70+ | 1.000 |  |  |  |  |  |  |
|  | Female | 0-4 | 6.123 | -0.385 | 0.229 |  |  |  | 11 |
|  |  | 5-14 | 6.998 | -0.475 | -0.595 |  |  |  | 41 |
|  |  | 15-29 | 4.424 | 0.000 | -0.644 | -0.027 | 0.009 |  | 35 |
|  |  | 30-44 | -4.979 | 2.070 | -0.364 | -0.149 | 0.014 |  | 25 |
|  |  | 45-59 | -5.366 | 2.130 | -0.207 | -0.147 | 0.013 |  | 18 |
|  |  | 60-69 | 1.000 |  |  |  |  |  |  |
|  |  | 70+ | 1.000 |  |  |  |  |  |  |
| *Intentional injuries* | | |  |  |  |  |  |  |  |
|  | Male | 0-4 | 1.000 |  |  |  |  |  |  |
|  |  | 5-14 | -15.954 | 4.258 | -0.584 | -0.278 | 0.022 |  | 33 |
|  |  | 15-29 | 4.003 | 0.000 | -0.871 | -0.017 | 0.027 |  | 25 |
|  |  | 30-44 | -7.348 | 2.770 | -0.629 | -0.183 | 0.025 |  | 22 |
|  |  | 45-59 | -8.132 | 2.976 | -0.305 | -0.191 | 0.014 |  | 11 |
|  |  | 60-69 | 1.000 |  |  |  |  |  |  |
|  |  | 70+ | 1.000 |  |  |  |  |  |  |
|  | Female | 0-4 | 1.000 |  |  |  |  |  |  |
|  |  | 5-14 | 1.420 |  | -0.792 | -0.023 | 0.023 |  | 40 |
|  |  | 15-29 | -5.731 | 2.336 | -0.311 | -0.166 | 0.008 |  | 17 |
|  |  | 30-44 | 2.551 |  |  | -0.012 | 0.007 |  | 5 |
|  |  | 45-59 | 1.000 |  |  |  |  |  |  |
|  |  | 60-69 | 1.000 |  |  |  |  |  |  |
|  |  | 70+ | 1.000 |  |  |  |  |  |  |
