# Supplementary material for: Projections of Global Mortality and Burden of Disease from 2002 to 2030
Source: PLoS Med. 2006 Nov 28;3(11):e442. doi: 10.1371/journal.pmed.0030442 (PMC1664601; doi:10.1371/journal.pmed.0030442)
Supplement: Table S5 — (765 KB DOC) [file pmed.0030442.st005.doc]

Table S5: Results of regressions of age-sex-specific mortality for detailed causes on the respective cause cluster based on the full country panel dataset, 1950-2002. Results are shown only when the r2≥ 0.25 and the p value for beta is 0.001 or less.

| **Cause-cluster** |  | **Sex** | **Age group** | **R^2^ (%)** | **Constant** | **Beta** |
| --- | --- | --- | --- | --- | --- | --- |
| *Group I* | | |  |  |  |  |
|  | Infectious and parasitic diseases | Male | 0-4 | 88 | -4.787 | 1.493 |
|  |  |  | 5-14 | 92 | -0.728 | 0.997 |
|  |  |  | 15-29 | 91 | -0.872 | 1.126 |
|  |  |  | 30-44 | 93 | -0.846 | 1.117 |
|  |  |  | 45-59 | 90 | -0.991 | 1.098 |
|  |  |  | 60-69 | 82 | -1.242 | 1.088 |
|  |  |  | 70+ | 61 | -0.752 | 0.887 |
|  | Infectious and parasitic diseases | Female | 0-4 | 89 | -4.500 | 1.473 |
|  |  |  | 5-14 | 92 | -0.787 | 1.001 |
|  |  |  | 15-29 | 90 | -0.894 | 0.996 |
|  |  |  | 30-44 | 92 | -0.853 | 1.005 |
|  |  |  | 45-59 | 92 | -0.874 | 1.063 |
|  |  |  | 60-69 | 86 | -0.968 | 1.020 |
|  |  |  | 70+ | 68 | -1.028 | 0.899 |
|  | Respiratory infections | Male | 0-4 | 83 | -4.625 | 1.476 |
|  |  |  | 5-14 | 86 | -0.808 | 0.936 |
|  |  |  | 15-29 | 69 | -0.644 | 0.763 |
|  |  |  | 30-44 | 62 | -0.485 | 0.741 |
|  |  |  | 45-59 | 77 | -0.354 | 0.826 |
|  |  |  | 60-69 | 77 | -0.018 | 0.834 |
|  |  |  | 70+ | 90 | -0.292 | 0.972 |
|  | Respiratory infections | Female | 0-4 | 85 | -4.489 | 1.484 |
|  |  |  | 5-14 | 86 | -0.744 | 0.927 |
|  |  |  | 15-29 | 77 | -0.909 | 0.741 |
|  |  |  | 30-44 | 76 | -0.635 | 0.699 |
|  |  |  | 45-59 | 83 | -0.455 | 0.820 |
|  |  |  | 60-69 | 85 | -0.351 | 0.893 |
|  |  |  | 70+ | 94 | -0.237 | 0.970 |
|  | Maternal conditions | Female | 15-29 | 80 | -1.593 | 1.097 |
|  |  |  | 30-44 | 80 | -2.130 | 1.194 |
|  |  |  | 45-59 | 63 | -4.783 | 1.122 |
|  | Perinatal conditions | Male | 0-4 | 73 | 1.619 | 0.609 |
|  | Perinatal conditions | Female | 0-4 | 66 | 1.745 | 0.556 |
|  | Nutritional deficiencies | Male | 0-4 | 59 | -7.705 | 1.567 |
|  |  |  | 5-14 | 76 | -1.961 | 0.858 |
|  |  |  | 15-29 | 49 | -2.045 | 0.775 |
|  |  |  | 30-44 | 41 | -2.768 | 0.852 |
|  |  |  | 45-59 | 55 | -3.396 | 1.061 |
|  |  |  | 60-69 | 68 | -4.276 | 1.288 |
|  |  |  | 70+ | 65 | -5.227 | 1.360 |
|  | Nutritional deficiencies | Female | 0-4 | 59 | -7.151 | 1.517 |
|  |  |  | 5-14 | 77 | -1.948 | 0.871 |
|  |  |  | 15-29 | 73 | -2.440 | 0.922 |
|  |  |  | 30-44 | 80 | -2.941 | 1.064 |
|  |  |  | 45-59 | 83 | -2.976 | 1.143 |
|  |  |  | 60-69 | 80 | -3.235 | 1.178 |
|  |  |  | 70+ | 67 | -3.733 | 1.156 |

Table S5 (continued): Results of regressions of age-sex-specific mortality for detailed causes on the respective cause cluster based on the full country panel dataset, 1950-2002. Results are shown only when the r2≥ 0.25 and the p value for beta is 0.001 or less.

| **Cause-cluster** |  | **Sex** | **Age group** | **R^2^ (%)** | **Constant** | **Beta** |
| --- | --- | --- | --- | --- | --- | --- |
| *Malignant neoplasms* | | |  |  |  |  |
|  | Mouth and oropharynx cancers | Male | 30-44 | 35 | -3.887 | 1.270 |
|  |  |  | 45-59 | 40 | -3.556 | 1.102 |
|  | Oesophagus cancer | Male | 30-44 | 33 | -3.625 | 1.032 |
|  |  |  | 45-59 | 39 | -3.443 | 1.051 |
|  | Stomach cancer | Female | 15-29 | 35 | -3.672 | 1.416 |
|  | Colon and rectum cancers | Male | 15-29 | 26 | -2.820 | 0.893 |
|  |  |  | 30-44 | 41 | -1.563 | 0.723 |
|  |  |  | 45-59 | 51 | -2.362 | 0.949 |
|  |  |  | 60-69 | 60 | -4.523 | 1.306 |
|  |  |  | 70+ | 65 | -6.086 | 1.511 |
|  | Colon and rectum cancers | Female | 15-29 | 35 | -3.244 | 1.066 |
|  |  |  | 45-59 | 34 | -3.256 | 1.141 |
|  |  |  | 60-69 | 43 | -4.061 | 1.299 |
|  |  |  | 70+ | 55 | -4.089 | 1.307 |
|  | Liver cancer | Female | 15-29 | 37 | -4.580 | 1.321 |
|  | Pancreas cancer | Male | 30-44 | 39 | -2.843 | 0.900 |
|  |  |  | 45-59 | 52 | -2.851 | 0.957 |
|  |  |  | 60-69 | 52 | -3.790 | 1.106 |
|  |  |  | 70+ | 57 | -4.129 | 1.128 |
|  | Pancreas cancer | Female | 45-59 | 49 | -5.473 | 1.401 |
|  |  |  | 60-69 | 56 | -4.601 | 1.270 |
|  |  |  | 70+ | 63 | -4.108 | 1.186 |
|  | Trachea, bronchus and lung cancers | Male | 30-44 | 52 | -2.512 | 1.210 |
|  |  |  | 45-59 | 78 | -3.493 | 1.414 |
|  |  |  | 60-69 | 77 | -4.490 | 1.500 |
|  |  |  | 70+ | 62 | -3.858 | 1.316 |
|  | Trachea, bronchus and lung cancers | Female | 15-29 | 35 | -3.701 | 1.172 |
|  |  |  | 60-69 | 32 | -3.292 | 1.169 |
|  |  |  | 70+ | 33 | -2.036 | 0.936 |
|  | Melanoma and other skin cancers | Male | 60-69 | 28 | -5.409 | 1.112 |
|  |  | Female | 45-59 | 32 | -8.409 | 1.772 |
|  |  |  | 60-69 | 39 | -7.872 | 1.538 |
|  | Breast cancer | Female | 15-29 | 27 | -2.236 | 0.756 |
|  |  |  | 45-59 | 31 | -1.467 | 1.000 |
|  |  |  | 60-69 | 35 | -2.791 | 1.151 |
|  |  |  | 70+ | 44 | -3.168 | 1.143 |
|  | Cervix uteri cancer | Female | 30-44 | 34 | -4.968 | 1.726 |
|  | Corpus uteri cancer | Female | 15-29 | 28 | -4.455 | 1.515 |
|  | Ovary cancer | Female | 30-44 | 28 | -3.164 | 1.072 |
|  |  |  | 45-59 | 44 | -4.772 | 1.420 |
|  |  |  | 60-69 | 47 | -5.865 | 1.510 |
|  |  |  | 70+ | 49 | -6.407 | 1.459 |
|  | Prostate cancer | Male | 15-29 | 27 | -4.864 | 0.977 |
|  |  |  | 70+ | 39 | -4.678 | 1.370 |
|  | Bladder cancer | Male | 45-59 | 43 | -3.822 | 0.977 |
|  |  |  | 60-69 | 44 | -4.062 | 1.084 |
|  |  |  | 70+ | 43 | -3.785 | 1.085 |
|  | Bladder cancer | Female | 60-69 | 26 | -3.368 | 0.831 |
|  |  |  | 70+ | 33 | -2.406 | 0.793 |
|  | Lymphomas and multiple myeloma | Male | 0-4 | 51 | -2.537 | 1.226 |
|  |  |  | 5-14 | 46 | -2.112 | 1.146 |
|  |  |  | 30-44 | 29 | -1.101 | 0.687 |
|  |  |  | 70+ | 56 | -6.194 | 1.352 |

Table S5 (continued): Results of regressions of age-sex-specific mortality for detailed causes on the respective cause cluster based on the full country panel dataset, 1950-2002. Results are shown only when the r2≥ 0.25 and the p value for beta is 0.001 or less.

| **Cause-cluster** |  | **Sex** | **Age group** | **R^2^ (%)** | **Constant** | **Beta** |
| --- | --- | --- | --- | --- | --- | --- |
| *Malignant neoplasms (continued)* | | |  |  |  |  |
|  | Lymphomas and multiple myeloma | Female | 0-4 | 37 | -2.297 | 1.020 |
|  |  |  | 5-14 | 32 | -2.258 | 0.946 |
|  |  |  | 15-29 | 29 | -1.745 | 0.834 |
|  |  |  | 30-44 | 27 | -2.609 | 0.845 |
|  |  |  | 60-69 | 32 | -3.712 | 1.066 |
|  |  |  | 70+ | 59 | -7.336 | 1.585 |
|  | Leukaemia | Male | 0-4 | 65 | -0.855 | 1.000 |
|  |  |  | 5-14 | 60 | -0.718 | 0.957 |
|  |  |  | 15-29 | 41 | -0.896 | 0.779 |
|  |  |  | 60-69 | 31 | -1.967 | 0.726 |
|  |  |  | 70+ | 45 | -5.786 | 1.273 |
|  | Leukaemia | Female | 0-4 | 67 | -0.869 | 1.020 |
|  |  |  | 5-14 | 62 | -0.754 | 0.986 |
|  |  |  | 15-29 | 52 | -1.427 | 0.948 |
|  |  |  | 30-44 | 32 | -2.001 | 0.748 |
|  |  |  | 70+ | 28 | -4.085 | 1.045 |
|  | Other malignant neoplasms | Male | 0-4 | 57 | -0.325 | 0.777 |
|  |  |  | 5-14 | 43 | -0.463 | 0.718 |
|  |  |  | 15-29 | 61 | -0.959 | 1.023 |
|  |  |  | 30-44 | 59 | -1.238 | 1.030 |
|  |  |  | 45-59 | 50 | -0.487 | 0.839 |
|  |  |  | 60-69 | 33 | 0.473 | 0.699 |
|  |  |  | 70+ |  |  |  |
|  | Other malignant neoplasms | Female | 0-4 | 61 | -0.436 | 0.830 |
|  |  |  | 5-14 | 44 | -0.427 | 0.742 |
|  |  |  | 15-29 | 64 | -1.123 | 1.021 |
|  |  |  | 30-44 | 50 | -2.409 | 1.218 |
|  |  |  | 45-59 | 44 | -2.968 | 1.283 |
|  |  |  | 60-69 | 40 | -2.444 | 1.173 |
|  |  |  | 70+ | 43 | -1.627 | 1.034 |
| *Cardiovascular diseases* | |  |  |  |  |  |
|  | Rheumatic heart disease | Male | 0-4 | 35 | -3.024 | 0.782 |
|  |  |  | 5-14 | 42 | -1.671 | 0.841 |
|  |  |  | 15-29 | 33 | -2.940 | 1.140 |
|  |  |  | 30-44 | 45 | -4.811 | 1.305 |
|  |  |  | 45-59 | 48 | -6.540 | 1.375 |
|  |  |  | 60-69 | 37 | -5.425 | 1.054 |
|  | Rheumatic heart disease | Female | 0-4 | 28 | -2.758 | 0.714 |
|  |  |  | 5-14 | 42 | -1.581 | 0.843 |
|  |  |  | 15-29 | 44 | -2.365 | 1.105 |
|  |  |  | 30-44 | 42 | -3.176 | 1.173 |
|  |  |  | 45-59 | 44 | -3.654 | 1.082 |
|  |  |  | 60-69 | 28 | -2.513 | 0.754 |
|  | Hypertensive heart disease | Male | 0-4 | 41 | -3.566 | 0.903 |
|  |  |  | 5-14 | 47 | -3.050 | 0.707 |
|  |  |  | 15-29 | 52 | -3.703 | 1.102 |
|  |  |  | 30-44 | 44 | -4.046 | 1.178 |
|  |  |  | 45-59 | 32 | -4.120 | 1.136 |
|  | Hypertensive heart disease | Female | 0-4 | 48 | -3.800 | 1.028 |
|  |  |  | 5-14 | 41 | -2.750 | 0.663 |
|  |  |  | 15-29 | 71 | -3.738 | 1.244 |
|  |  |  | 30-44 | 69 | -4.622 | 1.500 |
|  |  |  | 45-59 | 59 | -5.135 | 1.445 |
|  |  |  | 60-69 | 37 | -4.845 | 1.285 |

Table S5 (continued): Results of regressions of age-sex-specific mortality for detailed causes on the respective cause cluster based on the full country panel dataset, 1950-2002. Results are shown only when the r2≥ 0.25 and the p value for beta is 0.001 or less.

| **Cause-cluster** |  | **Sex** | **Age group** | **R^2^ (%)** | **Constant** | **Beta** |
| --- | --- | --- | --- | --- | --- | --- |
| *Cardiovascular disease (continued)* | | |  |  |  |  |
|  | Ischaemic heart disease | Male | 0-4 | 56 | -3.280 | 1.062 |
|  |  |  | 5-14 | 47 | -2.579 | 0.907 |
|  |  |  | 15-29 | 54 | -1.455 | 0.959 |
|  |  |  | 30-44 | 61 | -0.850 | 1.004 |
|  |  |  | 45-59 | 62 | -1.027 | 1.053 |
|  |  |  | 60-69 | 55 | -1.416 | 1.088 |
|  |  |  | 70+ | 37 | -1.773 | 1.089 |
|  | Ischaemic heart disease | Female | 0-4 | 60 | -3.565 | 1.139 |
|  |  |  | 5-14 | 47 | -2.657 | 0.886 |
|  |  |  | 15-29 | 64 | -2.212 | 1.057 |
|  |  |  | 30-44 | 60 | -1.291 | 0.936 |
|  |  |  | 45-59 | 59 | -0.814 | 0.934 |
|  |  |  | 60-69 | 54 | -0.862 | 0.976 |
|  |  |  | 70+ | 30 | -1.541 | 1.043 |
|  | Cerebrovascular disease | Male | 0-4 | 63 | -1.357 | 0.802 |
|  |  |  | 5-14 | 76 | -1.260 | 0.906 |
|  |  |  | 15-29 | 71 | -1.238 | 0.883 |
|  |  |  | 30-44 | 73 | -1.607 | 0.986 |
|  |  |  | 45-59 | 65 | -2.313 | 1.119 |
|  |  |  | 60-69 | 63 | -2.563 | 1.152 |
|  |  |  | 70+ | 59 | -1.535 | 1.020 |
|  | Cerebrovascular disease | Female | 0-4 | 63 | -1.501 | 0.819 |
|  |  |  | 5-14 | 76 | -1.202 | 0.821 |
|  |  |  | 15-29 | 71 | -0.785 | 0.726 |
|  |  |  | 30-44 | 79 | -0.664 | 0.837 |
|  |  |  | 45-59 | 82 | -1.530 | 1.068 |
|  |  |  | 60-69 | 77 | -2.016 | 1.118 |
|  |  |  | 70+ | 59 | -1.068 | 0.978 |
|  | Other cardiac diseases | Male | 0-4 | 96 | -0.199 | 0.986 |
|  |  |  | 5-14 | 91 | -0.478 | 0.966 |
|  |  |  | 15-29 | 70 | -0.512 | 0.858 |
|  |  |  | 30-44 | 49 | -0.545 | 0.781 |
|  | Other cardiac diseases | Female | 0-4 | 96 | -0.171 | 0.983 |
|  |  |  | 5-14 | 91 | -0.510 | 0.978 |
|  |  |  | 15-29 | 80 | -0.649 | 0.916 |
|  |  |  | 30-44 | 68 | -1.109 | 0.952 |
|  |  |  | 45-59 | 31 | 0.090 | 0.655 |
| *Digestive diseases* | |  |  |  |  |  |
|  | Peptic ulcer | Male | 0-4 | 68 | -3.407 | 0.847 |
|  |  |  | 5-14 | 56 | -3.029 | 0.890 |
|  |  |  | 15-29 | 70 | -2.377 | 1.040 |
|  |  |  | 30-44 | 45 | -2.400 | 0.943 |
|  |  |  | 45-59 | 41 | -1.703 | 0.820 |
|  |  |  | 60-69 | 37 | -0.910 | 0.744 |
|  |  |  | 70+ | 36 | -0.309 | 0.727 |
|  | Peptic ulcer | Female | 0-4 | 40 | -2.715 | 0.532 |
|  |  |  | 15-29 | 71 | -2.799 | 1.054 |
|  |  |  | 30-44 | 56 | -2.843 | 0.975 |
|  |  |  | 45-59 | 47 | -2.403 | 0.871 |
|  |  |  | 60-69 | 43 | -1.738 | 0.821 |
|  |  |  | 70+ | 44 | -1.691 | 0.921 |

Table S5 (continued): Results of regressions of age-sex-specific mortality for detailed causes on the respective cause cluster based on the full country panel dataset, 1950-2002. Results are shown only when the r2≥ 0.25 and the p value for beta is 0.001 or less.

| **Cause-cluster** |  | **Sex** | **Age group** | **R^2^ (%)** | **Constant** | **Beta** |
| --- | --- | --- | --- | --- | --- | --- |
| *Digestive diseases (continued)* | | |  |  |  |  |
|  | Cirrhosis of the liver | Male | 5-14 | 36 | -1.584 | 0.634 |
|  |  |  | 15-29 | 50 | -1.260 | 0.875 |
|  |  |  | 30-44 | 79 | -1.062 | 1.005 |
|  |  |  | 45-59 | 89 | -0.730 | 1.044 |
|  |  |  | 60-69 | 90 | -0.895 | 1.090 |
|  |  |  | 70+ | 83 | -1.732 | 1.208 |
|  | Cirrhosis of the liver | Female | 5-14 | 52 | -2.703 | 1.247 |
|  |  |  | 15-29 | 35 | -1.422 | 0.573 |
|  |  |  | 30-44 | 48 | -1.195 | 0.802 |
|  |  |  | 45-59 | 67 | -1.067 | 0.920 |
|  |  |  | 60-69 | 77 | -0.651 | 0.984 |
|  |  |  | 70+ | 82 | -0.928 | 1.078 |
|  | Appendicitis | Male | 5-14 | 74 | -1.406 | 1.126 |
|  |  |  | 15-29 | 28 | -1.107 | 0.901 |
|  |  |  | 30-44 | 72 | -1.638 | 0.824 |
|  |  |  | 60-69 | 57 | -2.739 | 0.937 |
|  |  |  | 70+ | 25 | -3.435 | 0.754 |
|  | Appendicitis | Female | 5-14 | 28 | -4.298 | 0.927 |
|  |  |  | 15-29 | 31 | -4.144 | 0.945 |
|  |  |  | 30-44 | 66 | -1.629 | 0.781 |
|  |  |  | 45-59 | 73 | -2.736 | 1.122 |
|  |  |  | 60-69 | 45 | -3.935 | 1.081 |
|  |  |  | 70+ | 37 | -4.757 | 1.100 |
|  | Other digestive diseases | Male | 0-4 | 43 | -5.754 | 1.292 |
|  |  |  | 5-14 | 36 | -4.817 | 1.035 |
|  |  |  | 15-29 | 99 | -0.122 | 1.004 |
|  |  |  | 30-44 | 89 | -0.490 | 0.969 |
|  |  |  | 45-59 | 85 | -0.519 | 0.883 |
|  |  |  | 60-69 | 76 | -0.740 | 0.859 |
|  |  |  | 70+ | 62 | -0.442 | 0.788 |
|  | Other digestive diseases | Female | 0-4 | 45 | 0.397 | 0.692 |
|  |  |  | 5-14 | 50 | -0.303 | 0.922 |
|  |  |  | 15-29 | 99 | -0.142 | 1.009 |
|  |  |  | 30-44 | 90 | -0.490 | 1.004 |
|  |  |  | 45-59 | 85 | -0.509 | 0.912 |
|  |  |  | 60-69 | 69 | -0.629 | 0.857 |
|  |  |  | 70+ | 57 | -0.187 | 0.744 |
| *Respiratory diseases* | |  |  |  |  |  |
|  | Other respiratory diseases | Male | 0-4 | 84 | 0.007 | 0.772 |
|  |  |  | 5-14 | 81 | -0.642 | 0.993 |
|  |  |  | 15-29 | 82 | -0.779 | 1.226 |
|  |  |  | 30-44 | 85 | -0.962 | 1.172 |
|  |  |  | 45-59 | 62 | -1.405 | 1.106 |
|  |  |  | 60-69 | 27 | -0.647 | 0.871 |
|  | Other respiratory diseases | Female | 0-4 | 84 | -0.001 | 0.753 |
|  |  |  | 5-14 | 83 | -0.595 | 0.943 |
|  |  |  | 15-29 | 84 | -0.776 | 1.159 |
|  |  |  | 30-44 | 83 | -0.965 | 1.130 |
|  |  |  | 45-59 | 66 | -1.671 | 1.204 |
|  |  |  | 60-69 | 51 | -1.995 | 1.178 |
|  |  |  | 70+ | 51 | -2.830 | 1.281 |

Table S5 (continued): Results of regressions of age-sex-specific mortality for detailed causes on the respective cause cluster based on the full country panel dataset, 1950-2002. Results are shown only when the r2≥ 0.25 and the p value for beta is 0.001 or less.

| **Cause-cluster** |  | **Sex** | **Age group** | **R^2^ (%)** | **Constant** | **Beta** |
| --- | --- | --- | --- | --- | --- | --- |
| *Other Group II* | |  |  |  |  |  |
|  | Endocrine disorders | Male | 0-4 | 39 | -3.682 | 1.131 |
|  | Endocrine disorders | Female | 0-4 | 44 | -4.228 | 1.249 |
|  |  |  | 15-29 | 31 | -3.298 | 0.984 |
|  |  |  | 30-44 | 37 | -6.435 | 1.484 |
|  | Neuro-psychiatric conditions | Male | 0-4 | 40 | -1.542 | 0.789 |
|  |  |  | 5-14 | 56 | -1.323 | 0.883 |
|  |  |  | 15-29 | 47 | -1.102 | 0.803 |
|  |  |  | 30-44 | 44 | -2.821 | 1.039 |
|  | Neuro-psychiatric conditions | Female | 0-4 | 41 | -1.576 | 0.787 |
|  |  |  | 5-14 | 53 | -1.111 | 0.791 |
|  |  |  | 15-29 | 65 | -1.480 | 0.823 |
|  |  |  | 30-44 | 53 | -2.952 | 0.997 |
|  | Genito-urinary diseases | Male | 0-4 | 45 | -5.760 | 1.363 |
|  |  |  | 5-14 | 60 | -5.337 | 1.717 |
|  |  |  | 15-29 | 57 | -5.946 | 1.740 |
|  |  |  | 30-44 | 57 | -6.471 | 1.549 |
|  |  |  | 45-59 | 35 | -6.084 | 1.300 |
|  | Genito-urinary diseases | Female | 0-4 | 45 | -5.771 | 1.367 |
|  |  |  | 5-14 | 63 | -4.676 | 1.585 |
|  |  |  | 15-29 | 75 | -5.737 | 1.828 |
|  |  |  | 30-44 | 68 | -8.100 | 2.005 |
|  |  |  | 45-59 | 51 | -7.524 | 1.611 |
|  |  |  | 60-69 | 30 | -4.452 | 1.074 |
|  | Musculo-skeletal diseases | Male | 0-4 | 39 | -5.960 | 0.947 |
|  |  |  | 5-14 | 44 | -4.558 | 0.865 |
|  | Musculo-skeletal diseases | Female | 5-14 | 33 | -3.239 | 0.621 |
|  | Congenital anomalies | Male | 0-4 | 33 | 1.581 | 0.542 |
|  | Congenital anomalies | Female | 0-4 | 29 | 1.673 | 0.509 |
| *Other unintentional injuries* | |  |  |  |  |  |
|  | Poisonings | Male | 0-4 | 45 | -3.538 | 1.191 |
|  |  |  | 30-44 | 33 | -3.345 | 1.148 |
|  |  |  | 45-59 | 44 | -4.928 | 1.483 |
|  |  |  | 60-69 | 37 | -4.755 | 1.417 |
|  | Poisonings | Female | 0-4 | 46 | -3.233 | 1.170 |
|  |  |  | 30-44 | 37 | -2.908 | 1.234 |
|  |  |  | 45-59 | 47 | -3.988 | 1.533 |
|  |  |  | 60-69 | 30 | -4.049 | 1.423 |
|  | Falls | Male | 0-4 | 50 | -1.968 | 0.743 |
|  |  |  | 5-14 | 47 | -2.714 | 0.929 |
|  |  |  | 30-44 | 32 | -2.356 | 0.817 |
|  |  |  | 45-59 | 40 | -1.294 | 0.692 |
|  |  |  | 60-69 | 37 | -0.614 | 0.637 |
|  |  |  | 70+ | 28 | -0.007 | 0.593 |
|  | Falls | Female | 0-4 | 41 | -1.758 | 1.100 |
|  |  |  | 5-14 | 55 | -2.202 | 0.780 |
|  |  |  | 30-44 | 35 | -2.796 | 0.871 |
|  |  |  | 45-59 | 36 | -2.079 | 0.744 |
|  |  |  | 70+ | 26 | -0.884 | 0.539 |
|  | Fires | Male | 0-4 | 51 | -2.195 | 0.952 |
|  |  |  | 5-14 | 33 | -2.589 | 0.770 |
|  |  |  | 30-44 | 51 | -3.147 | 0.879 |
|  |  |  | 45-59 | 56 | -3.477 | 0.983 |
|  |  |  | 60-69 | 48 | -3.310 | 0.997 |

Table S5 (continued): Results of regressions of age-sex-specific mortality for detailed causes on the respective cause cluster based on the full country panel dataset, 1950-2002. Results are shown only when the r2≥ 0.25 and the p value for beta is 0.001 or less.

| **Cause-cluster** |  | **Sex** | **Age group** | **R^2^ (%)** | **Constant** | **Beta** |
| --- | --- | --- | --- | --- | --- | --- |
| *Other unintentional injuries (continued)* | |  |  |  |  |  |
|  | Fires | Female | 0-4 | 55 | -2.094 | 0.961 |
|  |  |  | 5-14 | 29 | -2.113 | 0.771 |
|  |  |  | 30-44 | 30 | -2.554 | 0.896 |
|  |  |  | 45-59 | 44 | -2.729 | 0.913 |
|  |  |  | 60-69 | 37 | -3.129 | 1.056 |
|  | Drownings | Male | 0-4 | 66 | -1.677 | 1.005 |
|  |  |  | 5-14 | 67 | -2.383 | 1.280 |
|  |  |  | 15-29 | 40 | -3.347 | 1.231 |
|  |  |  | 30-44 | 70 | -3.757 | 1.275 |
|  |  |  | 45-59 | 68 | -3.433 | 1.164 |
|  |  |  | 60-69 | 66 | -3.902 | 1.235 |
|  | Drownings | Female | 0-4 | 64 | -1.870 | 1.014 |
|  |  |  | 5-14 | 65 | -2.849 | 1.459 |
|  |  |  | 15-29 | 33 | -3.897 | 1.445 |
|  |  |  | 30-44 | 68 | -4.075 | 1.415 |
|  |  |  | 45-59 | 67 | -3.683 | 1.217 |
|  |  |  | 60-69 | 36 | -3.368 | 1.079 |
|  | Other unintentional injuries | Male | 0-4 | 83 | -1.029 | 1.034 |
|  |  |  | 5-14 | 73 | -1.726 | 1.116 |
|  |  |  | 15-29 | 52 | -2.240 | 1.181 |
|  |  |  | 30-44 | 76 | -1.660 | 1.091 |
|  |  |  | 45-59 | 77 | -1.474 | 1.060 |
|  |  |  | 60-69 | 72 | -1.818 | 1.130 |
|  |  |  | 70+ | 42 | -1.328 | 0.979 |
|  | Other unintentional injuries | Female | 0-4 | 85 | -1.008 | 1.043 |
|  |  |  | 5-14 | 68 | -2.009 | 1.220 |
|  |  |  | 15-29 | 39 | -2.059 | 1.144 |
|  |  |  | 30-44 | 71 | -2.111 | 1.256 |
|  |  |  | 45-59 | 72 | -1.797 | 1.154 |
|  |  |  | 60-69 | 61 | -2.100 | 1.216 |
| *Intentional injuries* | |  |  |  |  |  |
|  | Self-inflicted injuries | Male | 5-14 | 38 | -0.413 | 0.692 |
|  |  |  | 15-29 | 27 | 1.103 | 0.534 |
|  |  |  | 30-44 | 36 | 0.765 | 0.665 |
|  |  |  | 45-59 | 37 | 0.777 | 0.691 |
|  |  |  | 60-69 | 56 | 0.313 | 0.853 |
|  |  |  | 70+ | 51 | -0.850 | 0.838 |
|  | Self-inflicted injuries | Female | 5-14 | 65 | -0.106 | 0.842 |
|  |  |  | 15-29 | 51 | -0.063 | 0.817 |
|  |  |  | 30-44 | 58 | -0.205 | 0.936 |
|  |  |  | 45-59 | 73 | -0.418 | 1.061 |
|  |  |  | 60-69 | 79 | -0.504 | 1.082 |
|  |  |  | 70+ | 90 | 0.043 | 0.886 |
|  | Homicide | Male | 0-4 | 57 | -0.721 | 0.947 |
|  |  |  | 5-14 | 63 | -2.629 | 1.385 |
|  |  |  | 15-29 | 57 | -2.605 | 1.325 |
|  |  |  | 30-44 | 44 | -2.439 | 1.217 |
|  |  |  | 45-59 | 37 | -2.881 | 1.296 |
|  |  |  | 60-69 | 81 | 0.082 | 0.785 |
|  | Homicide | Female | 0-4 | 70 | -0.456 | 0.813 |
|  |  |  | 5-14 | 51 | -1.290 | 1.006 |
|  |  |  | 15-29 | 41 | -1.237 | 0.989 |
|  |  |  | 30-44 | 27 | -8.052 | 2.039 |
|  | War | Male | 45-59 | 46 | -11.311 | 3.022 |
|  |  |  | 60-69 | 30 | -11.546 | 2.664 |
|  |  |  | 70+ | 38 | -0.413 | 0.692 |
|  |  |  |  |  |  |  |
