# Supplementary material for: Projections of Global Mortality and Burden of Disease from 2002 to 2030
Source: PLoS Med. 2006 Nov 28;3(11):e442. doi: 10.1371/journal.pmed.0030442 (PMC1664601; doi:10.1371/journal.pmed.0030442)
Supplement: Table S6 — (1.2 MB DOC) [file pmed.0030442.st006.doc]

Table S6:Results of log-linear Poisson regressions for deaths due to selected causes, by age and sex, for countries with complete death registration data and population over 5 million. Results are shown only when the p value for beta is 0.001 or less.

| **WHO Region** |  |  | **Beta** | | | | |
| --- | --- | --- | --- | --- | --- | --- | --- |
| **Country** | **Sex** | **Age** | **TB** | **IHD** | **Stroke** | **Suicide** | **Homi-cide** |
| *AMRO Region* |  |  |  |  |  |  |  |
| Argentina | M | 0-4 | 0.92 |  |  |  | 1 |
|  |  | 5-14 | 0.93 |  |  |  | 1 |
|  |  | 15-29 | 0.94 | 0.97 | 0.97 | 1.06 | 1.05 |
|  |  | 30-44 | 0.94 | 0.97 | 0.97 | 1.02 | 1.01 |
|  |  | 45-59 | 0.94 | 1.02 | 0.98 | 1.01 | 1 |
|  |  | 60-69 | 0.96 | 1.03 | 0.98 | 1 | 1 |
|  |  | 70-79 | 0.96 | 1.04 | 0.98 | 1 | 1 |
|  |  | 80+ | 0.96 | 1.04 | 0.98 | 1 | 1 |
| Argentina | F | 0-4 | 0.94 |  |  |  | 1 |
|  |  | 5-14 | 0.94 |  |  |  | 1 |
|  |  | 15-29 | 0.94 | 0.94 | 0.97 | 1 | 1 |
|  |  | 30-44 | 0.93 | 0.97 | 0.96 | 0.98 | 1 |
|  |  | 45-59 | 0.95 | 1.03 | 0.97 | 1 | 1 |
|  |  | 60-69 | 0.96 | 1.04 | 0.97 | 0.96 | 1 |
|  |  | 70-79 | 0.96 | 1.02 | 0.97 | 0.96 | 1 |
|  |  | 80+ | 0.96 | 1.02 | 0.97 | 0.96 | 1 |
| Brazil | M | 0-4 | 0.89 |  |  |  | 1.04 |
|  |  | 5-14 | 0.93 |  |  |  | 1.04 |
|  |  | 15-29 | 0.96 | 0.97 | 0.96 | 1.01 | 1.04 |
|  |  | 30-44 | 0.98 | 0.97 | 0.95 | 1.01 | 1.01 |
|  |  | 45-59 | 0.98 | 0.99 | 0.96 | 1.01 | 1.01 |
|  |  | 60-69 | 1 | 1.01 | 0.97 | 1.02 | 1.01 |
|  |  | 70-79 | 1 | 1 | 0.98 | 1 | 1.01 |
|  |  | 80+ | 1 | 1 | 0.98 | 1 | 1.01 |
| Brazil | F | 0-4 | 0.9 |  |  |  | 1.03 |
|  |  | 5-14 | 0.94 |  |  |  | 1.05 |
|  |  | 15-29 | 0.96 | 0.97 | 0.96 | 0.99 | 1.03 |
|  |  | 30-44 | 0.98 | 0.98 | 0.96 | 1 | 1.02 |
|  |  | 45-59 | 0.98 | 1 | 0.97 | 1 | 1.02 |
|  |  | 60-69 | 1 | 1.01 | 0.97 | 0.97 | 1 |
|  |  | 70-79 | 1 | 0.99 | 0.97 | 1 | 1 |
|  |  | 80+ | 1 | 0.99 | 0.97 | 1 | 1 |
| Canada | M | 0-4 |  |  |  |  | 1 |
|  |  | 5-14 |  |  |  |  | 1 |
|  |  | 15-29 |  | 0.95 | 0.94 | 0.98 | 1 |
|  |  | 30-44 | 1 | 0.96 | 0.96 | 1 | 0.98 |
|  |  | 45-59 | 0.91 | 0.95 | 0.96 | 1 | 0.95 |
|  |  | 60-69 | 0.95 | 0.95 | 0.97 | 0.98 | 1 |
|  |  | 70-79 | 0.96 | 0.97 | 0.98 | 0.98 | 1 |
|  |  | 80+ | 0.96 | 0.97 | 0.98 | 0.98 | 1 |
| Canada | F | 0-4 |  |  |  |  | 1 |
|  |  | 5-14 |  |  |  |  | 1 |
|  |  | 15-29 |  |  | 0.94 | 1 | 0.94 |
|  |  | 30-44 | 1 |  | 0.96 | 1 | 0.96 |
|  |  | 45-59 | 1 | 0.95 | 0.96 | 1 | 0.94 |
|  |  | 60-69 | 1 | 0.95 | 0.97 | 0.97 | 1 |
|  |  | 70-79 | 0.97 | 0.97 | 0.99 | 0.97 | 1 |
|  |  | 80+ | 0.97 | 0.97 | 0.99 | 0.97 | 1 |
| **WHO Region** |  |  | **Beta** | | | | |
| **Country** | **Sex** | **Age** | **TB** | **IHD** | **Stroke** | **Suicide** | **Homi-cide** |
| Chile | M | 0-4 |  |  |  |  | 1 |
|  |  | 5-14 |  |  |  |  | 1 |
|  |  | 15-29 | 0.9 |  |  | 0.99 | 1 |
|  |  | 30-44 | 0.93 |  | 0.98 | 1 | 0.98 |
|  |  | 45-59 | 0.94 | 0.99 | 0.96 | 1 | 0.95 |
|  |  | 60-69 | 0.95 | 0.98 | 0.96 | 0.97 | 0.94 |
|  |  | 70-79 | 1 | 0.98 | 0.98 | 1 | 0.93 |
|  |  | 80+ | 1 | 0.98 | 0.98 | 1 | 0.93 |
| Chile | F | 0-4 |  |  |  |  | 1 |
|  |  | 5-14 |  |  |  |  | 1 |
|  |  | 15-29 | 0.89 |  |  | 1 | 1 |
|  |  | 30-44 | 0.9 |  | 0.98 | 1 | 1 |
|  |  | 45-59 | 0.94 | 0.98 | 0.96 | 1 | 1 |
|  |  | 60-69 | 1 | 0.98 | 0.95 | 1 | 1 |
|  |  | 70-79 | 1.03 | 0.96 | 0.98 | 1 | 1 |
|  |  | 80+ | 1.03 | 0.96 | 0.98 | 1 | 1 |
| Colombia | M | 0-4 | 0.92 |  |  |  | 1.07 |
|  |  | 5-14 | 0.88 |  |  |  | 1 |
|  |  | 15-29 | 0.94 | 0.96 | 0.96 | 1.05 | 1 |
|  |  | 30-44 | 0.95 | 0.97 | 0.97 | 1.05 | 0.98 |
|  |  | 45-59 | 0.95 | 0.97 | 0.96 | 1.04 | 0.98 |
|  |  | 60-69 | 0.96 | 1 | 0.98 | 1.06 | 1 |
|  |  | 70-79 | 0.96 | 1 | 0.98 | 1.07 | 1 |
|  |  | 80+ | 0.96 | 1 | 0.98 | 1.07 | 1 |
| Colombia | F | 0-4 | 0.9 |  |  |  | 1.07 |
|  |  | 5-14 | 0.92 |  |  |  | 1 |
|  |  | 15-29 | 0.94 | 0.96 | 0.96 | 1.07 | 1 |
|  |  | 30-44 | 0.92 | 0.96 | 0.96 | 1.06 | 0.99 |
|  |  | 45-59 | 0.92 | 0.96 | 0.95 | 1.06 | 1 |
|  |  | 60-69 | 0.96 | 1 | 0.99 | 1 | 1 |
|  |  | 70-79 | 0.95 | 0.99 | 0.98 | 1 | 1 |
|  |  | 80+ | 0.95 | 0.99 | 0.98 | 1 | 1 |
| Cuba | M | 0-4 |  |  |  |  | 1 |
|  |  | 5-14 |  |  |  |  | 1 |
|  |  | 15-29 |  |  |  | 1 | 0.94 |
|  |  | 30-44 |  | 0.96 | 0.96 | 0.98 | 0.97 |
|  |  | 45-59 |  | 0.98 | 0.98 | 0.98 | 0.95 |
|  |  | 60-69 |  | 0.98 | 0.99 | 0.96 | 0.93 |
|  |  | 70-79 | 0.94 | 0.98 |  | 0.96 | 1 |
|  |  | 80+ | 0.94 | 0.98 |  | 0.96 | 1 |
| Cuba | F | 0-4 |  |  |  |  | 1 |
|  |  | 5-14 |  |  |  |  | 1 |
|  |  | 15-29 |  |  |  | 0.88 | 0.96 |
|  |  | 30-44 |  | 0.94 | 0.96 | 0.91 | 1 |
|  |  | 45-59 |  | 0.97 | 0.97 | 0.92 | 1 |
|  |  | 60-69 |  | 0.98 | 0.99 | 0.93 | 1 |
|  |  | 70-79 |  | 0.98 |  | 0.92 | 1 |
|  |  | 80+ |  | 0.98 |  | 0.92 | 1 |

Table S6 (continued)

| **WHO Region** |  |  | **Beta** | | | | |
| --- | --- | --- | --- | --- | --- | --- | --- |
| **Country** | **Sex** | **Age** | **TB** | **IHD** | **Stroke** | **Suicide** | **Homi-cide** |
| Ecuador | M | 0-4 | 0.92 |  |  |  | 1 |
|  |  | 5-14 | 0.94 |  |  |  | 1 |
|  |  | 15-29 | 0.98 | 1.03 |  |  | 1.05 |
|  |  | 30-44 | 0.97 |  | 0.97 |  | 1.04 |
|  |  | 45-59 | 0.95 | 1.03 | 0.96 |  | 1.03 |
|  |  | 60-69 | 0.95 | 1.05 | 0.95 |  | 1 |
|  |  | 70-79 | 0.97 | 1.04 | 0.96 |  | 1 |
|  |  | 80+ | 0.97 | 1.04 | 0.96 |  | 1 |
| Ecuador | F | 0-4 | 0.92 |  |  |  | 1 |
|  |  | 5-14 | 0.92 |  |  |  | 1 |
|  |  | 15-29 | 0.95 |  |  |  | 1.04 |
|  |  | 30-44 | 0.94 |  | 0.96 |  | 1 |
|  |  | 45-59 | 0.94 | 1 | 0.96 |  | 1 |
|  |  | 60-69 | 0.97 | 1.04 | 0.96 |  | 1 |
|  |  | 70-79 | 0.94 | 1.02 | 0.95 |  | 1 |
|  |  | 80+ | 0.94 | 1.02 | 0.95 |  | 1 |
| El Salvador | M | 0-4 |  |  |  |  |  |
|  |  | 5-14 |  |  |  |  |  |
|  |  | 15-29 |  |  | 0.92 | 0.95 |  |
|  |  | 30-44 | 1 |  | 0.92 | 0.95 | 0.98 |
|  |  | 45-59 | 1 | 1.06 | 0.93 | 1 |  |
|  |  | 60-69 | 0.87 | 1.06 | 0.93 | 1 |  |
|  |  | 70-79 | 0.94 | 1.08 | 0.91 | 0.9 |  |
|  |  | 80+ | 0.94 | 1.08 | 0.91 | 0.9 |  |
| El Salvador | F | 0-4 |  |  |  |  |  |
|  |  | 5-14 |  |  |  |  |  |
|  |  | 15-29 |  |  | 0.85 | 0.93 |  |
|  |  | 30-44 | 1 |  | 0.92 | 0.93 | 1 |
|  |  | 45-59 | 0.88 | 1.05 | 0.9 | 1 |  |
|  |  | 60-69 | 0.91 | 1.08 | 0.9 | 1 |  |
|  |  | 70-79 | 0.85 | 1.05 | 0.88 | 1 |  |
|  |  | 80+ | 0.85 | 1.05 | 0.88 | 1 |  |
| Guatemala | M | 0-4 | 0.91 |  |  |  |  |
|  |  | 5-14 | 0.89 |  |  |  |  |
|  |  | 15-29 | 0.93 |  | 1.09 |  | 1 |
|  |  | 30-44 | 0.92 |  | 1.06 |  | 1 |
|  |  | 45-59 | 0.9 |  | 1.04 |  | 1.01 |
|  |  | 60-69 | 0.89 |  | 1.03 |  |  |
|  |  | 70-79 | 0.92 |  | 1.04 |  |  |
|  |  | 80+ | 0.92 |  | 1.04 |  |  |
| Guatemala | F | 0-4 | 0.91 |  |  |  |  |
|  |  | 5-14 | 0.9 |  |  |  |  |
|  |  | 15-29 | 0.9 |  | 1.05 |  | 0.97 |
|  |  | 30-44 | 0.9 |  | 1.03 |  | 0.97 |
|  |  | 45-59 | 0.9 |  | 1.03 |  | 1 |
|  |  | 60-69 | 0.9 |  | 1.03 |  |  |
|  |  | 70-79 | 0.88 |  | 1.03 |  |  |
|  |  | 80+ | 0.88 |  | 1.03 |  |  |

| **WHO Region** |  |  | **Beta** | | | | |
| --- | --- | --- | --- | --- | --- | --- | --- |
| **Country** | **Sex** | **Age** | **TB** | **IHD** | **Stroke** | **Suicide** | **Homi-cide** |
| Mexico | M | 0-4 | 0.86 |  |  |  | 0.96 |
|  |  | 5-14 | 0.88 |  | 0.95 |  | 0.97 |
|  |  | 15-29 | 0.94 |  | 0.99 | 1.05 | 0.95 |
|  |  | 30-44 | 0.93 | 1 | 0.99 | 1.03 | 0.95 |
|  |  | 45-59 | 0.92 | 1 | 0.99 | 1.02 | 0.94 |
|  |  | 60-69 | 0.93 | 1.01 | 0.99 | 1 | 0.94 |
|  |  | 70-79 | 0.93 | 1.02 | 0.99 | 1 | 0.95 |
|  |  | 80+ | 0.93 | 1.02 | 0.99 | 1 | 0.95 |
| Mexico | F | 0-4 | 0.83 |  |  |  | 0.97 |
|  |  | 5-14 | 0.89 |  | 0.95 |  | 0.97 |
|  |  | 15-29 | 0.92 | 0.98 | 0.95 | 1.05 | 0.98 |
|  |  | 30-44 | 0.91 | 0.99 | 0.96 | 1.03 | 0.97 |
|  |  | 45-59 | 0.92 | 1.01 | 0.98 | 1.03 | 0.96 |
|  |  | 60-69 | 0.93 | 1.02 | 0.99 | 1 | 0.96 |
|  |  | 70-79 | 0.93 | 1.02 | 0.99 | 1 | 0.96 |
|  |  | 80+ | 0.93 | 1.02 | 0.99 | 1 | 0.96 |
| USA | M | 0-4 |  |  |  |  | 1 |
|  |  | 5-14 |  |  |  |  | 0.97 |
|  |  | 15-29 | 0.9 |  | 0.98 | 0.98 | 0.98 |
|  |  | 30-44 | 0.89 | 0.99 | 0.98 | 1 | 0.95 |
|  |  | 45-59 | 0.91 | 0.98 | 0.98 | 0.99 | 0.95 |
|  |  | 60-69 | 0.92 | 0.98 | 0.98 | 0.97 | 0.95 |
|  |  | 70-79 | 0.93 | 0.99 | 0.99 | 0.97 | 0.95 |
|  |  | 80+ | 0.93 | 0.99 | 0.99 | 0.97 | 0.95 |
| USA | F | 0-4 |  |  |  |  | 1 |
|  |  | 5-14 |  |  |  |  | 0.97 |
|  |  | 15-29 | 0.91 |  | 0.97 | 0.98 | 0.96 |
|  |  | 30-44 | 0.9 | 1.01 | 0.99 | 0.99 | 0.97 |
|  |  | 45-59 | 0.92 | 0.98 | 0.98 | 0.99 | 0.97 |
|  |  | 60-69 | 0.92 | 0.98 | 0.99 | 0.96 | 0.96 |
|  |  | 70-79 | 0.95 | 1 | 0.99 | 0.96 | 0.95 |
|  |  | 80+ | 0.95 | 1 | 0.99 | 0.96 | 0.95 |
| Venezuela | M | 0-4 |  |  |  |  | 1 |
|  |  | 5-14 |  |  |  |  | 1.04 |
|  |  | 15-29 |  |  | 0.97 | 1.03 | 1.09 |
|  |  | 30-44 | 0.97 | 1.01 | 0.99 | 1.03 | 1.08 |
|  |  | 45-59 | 0.96 | 0.99 | 0.98 | 1 | 1.06 |
|  |  | 60-69 | 0.97 | 1 | 0.99 | 1 | 1.04 |
|  |  | 70-79 | 0.96 | 1 | 0.98 | 1 | 1.08 |
|  |  | 80+ | 0.96 | 1 | 0.98 | 1 | 1.08 |
| Venezuela | F | 0-4 |  |  |  |  | 1 |
|  |  | 5-14 |  |  |  |  | 1.06 |
|  |  | 15-29 |  |  | 0.97 | 1 | 1.07 |
|  |  | 30-44 | 0.96 | 1 | 0.99 | 1 | 1.04 |
|  |  | 45-59 | 0.93 | 1 | 0.97 | 1 | 1.11 |
|  |  | 60-69 | 0.96 | 1 | 0.98 | 1 | 1 |
|  |  | 70-79 | 0.96 | 1 | 0.98 | 1 | 1 |
|  |  | 80+ | 0.96 | 1 | 0.98 | 1 | 1 |

Table S6 (continued)

| **WHO Region** |  |  | **Beta** | | | | |
| --- | --- | --- | --- | --- | --- | --- | --- |
| **Country** | **Sex** | **Age** | **TB** | **IHD** | **Stroke** | **Suicide** | **Homi-cide** |
| *EURO Region* | |  |  |  |  |  |  |
| Austria | M | 0-4 |  |  |  |  |  |
|  |  | 5-14 |  |  |  |  |  |
|  |  | 15-29 |  |  |  | 0.98 |  |
|  |  | 30-44 | 0.92 | 0.97 | 0.96 | 0.97 | 0.93 |
|  |  | 45-59 | 0.9 | 0.97 | 0.96 | 0.98 |  |
|  |  | 60-69 | 0.88 | 0.96 | 0.95 | 0.98 |  |
|  |  | 70-79 | 0.9 | 0.99 | 0.95 | 0.98 |  |
|  |  | 80+ | 0.9 | 0.99 | 0.95 | 0.98 |  |
| Austria | F | 0-4 |  |  |  |  |  |
|  |  | 5-14 |  |  |  |  |  |
|  |  | 15-29 |  |  |  | 0.96 |  |
|  |  | 30-44 | 1 | 1 | 1 | 0.97 | 1 |
|  |  | 45-59 | 1 | 0.98 | 0.98 | 0.97 |  |
|  |  | 60-69 | 1 | 0.96 | 0.95 | 0.96 |  |
|  |  | 70-79 | 0.92 | 1 | 0.96 | 0.97 |  |
|  |  | 80+ | 0.92 | 1 | 0.96 | 0.97 |  |
| Belarus | M | 0-4 |  |  |  |  |  |
|  |  | 5-14 |  |  |  |  |  |
|  |  | 15-29 | 1.14 |  |  | 1.06 | 1.01 |
|  |  | 30-44 | 1.1 | 1.02 | 1.03 | 1.04 | 1.05 |
|  |  | 45-59 | 1.07 | 1.02 | 1.02 | 1.04 | 1.07 |
|  |  | 60-69 | 1.04 | 1.04 | 1.03 | 1.05 | 1.07 |
|  |  | 70-79 | 0.97 | 1 | 1 | 1.05 | 1.06 |
|  |  | 80+ | 0.97 | 1 | 1 | 1.05 | 1.06 |
| Belarus | F | 0-4 |  |  |  |  |  |
|  |  | 5-14 |  |  |  |  |  |
|  |  | 15-29 |  |  |  | 1.03 | 1.07 |
|  |  | 30-44 | 1.1 | 1.04 | 1.03 | 1.02 | 1.03 |
|  |  | 45-59 | 1 | 1.02 | 1.02 | 1 | 1.06 |
|  |  | 60-69 | 1 | 1.03 | 1.02 | 1 | 1.06 |
|  |  | 70-79 | 0.96 | 1 | 1 | 1 | 1.06 |
|  |  | 80+ | 0.96 | 1 | 1 | 1 | 1.06 |
| Bulgaria | M | 0-4 |  |  |  |  |  |
|  |  | 5-14 |  |  |  |  |  |
|  |  | 15-29 |  |  |  | 1 | 0.97 |
|  |  | 30-44 | 1 | 0.99 | 0.98 | 1 | 1 |
|  |  | 45-59 | 1 | 1 | 0.99 | 1.01 | 1 |
|  |  | 60-69 | 1 | 0.99 | 0.99 | 1 | 1 |
|  |  | 70-79 | 1 | 0.98 | 0.98 | 0.99 | 1 |
|  |  | 80+ | 1 | 0.98 | 0.98 | 0.99 | 1 |
| Bulgaria | F | 0-4 |  |  |  |  |  |
|  |  | 5-14 |  |  |  |  |  |
|  |  | 15-29 |  |  |  | 1 | 1 |
|  |  | 30-44 | 1 | 1 | 0.98 | 1 | 1 |
|  |  | 45-59 | 1 | 1 | 0.98 | 1 | 1 |
|  |  | 60-69 | 1 | 0.99 | 0.98 | 0.97 | 1 |
|  |  | 70-79 | 1 | 0.97 | 0.98 | 0.98 | 0.95 |
|  |  | 80+ | 1 | 0.97 | 0.98 | 0.98 | 0.95 |

| **WHO Region** |  |  | **Beta** | | | | |
| --- | --- | --- | --- | --- | --- | --- | --- |
| **Country** | **Sex** | **Age** | **TB** | **IHD** | **Stroke** | **Suicide** | **Homi-cide** |
| Czech Republic | M | 0-4 |  |  |  |  |  |
|  |  | 5-14 |  |  |  |  |  |
|  |  | 15-29 |  | 0.94 |  | 1 | 1 |
|  |  | 30-44 | 1 | 0.93 | 0.93 | 0.99 | 1 |
|  |  | 45-59 | 1 | 0.96 | 0.95 | 1 | 1 |
|  |  | 60-69 | 0.93 | 0.96 | 0.95 | 0.96 | 1 |
|  |  | 70-79 | 0.93 | 0.97 | 0.96 | 0.96 | 0.92 |
|  |  | 80+ | 0.93 | 0.97 | 0.96 | 0.96 | 0.92 |
| Czech Republic | F | 0-4 |  |  |  |  |  |
|  |  | 5-14 |  |  |  |  |  |
|  |  | 15-29 |  |  |  | 0.96 | 0.95 |
|  |  | 30-44 | 1 | 0.94 | 0.95 | 0.96 | 1 |
|  |  | 45-59 | 1 | 0.96 | 0.95 | 0.96 | 1 |
|  |  | 60-69 | 0.91 | 0.96 | 0.94 | 0.94 | 1 |
|  |  | 70-79 | 0.93 | 0.97 | 0.97 | 0.93 | 1 |
|  |  | 80+ | 0.93 | 0.97 | 0.97 | 0.93 | 1 |
| Denmark | M | 0-4 |  |  |  |  |  |
|  |  | 5-14 |  |  |  |  |  |
|  |  | 15-29 |  |  |  | 0.98 | 1 |
|  |  | 30-44 |  | 0.94 |  | 0.96 | 1 |
|  |  | 45-59 | 1 | 0.93 | 0.97 | 0.95 | 1 |
|  |  | 60-69 | 1 | 0.94 | 0.98 | 0.95 |  |
|  |  | 70-79 | 1 | 0.96 | 0.99 | 0.97 |  |
|  |  | 80+ | 1 | 0.96 | 0.99 | 0.97 |  |
| Denmark | F | 0-4 |  |  |  |  |  |
|  |  | 5-14 |  |  |  |  |  |
|  |  | 15-29 |  |  |  | 0.94 | 1 |
|  |  | 30-44 |  | 1 |  | 0.91 | 1 |
|  |  | 45-59 | 1 | 0.92 | 0.97 | 0.92 | 1 |
|  |  | 60-69 | 1 | 0.94 | 0.98 | 0.92 |  |
|  |  | 70-79 | 1 | 0.96 | 0.99 | 0.93 |  |
|  |  | 80+ | 1 | 0.96 | 0.99 | 0.93 |  |
| Finland | M | 0-4 |  |  |  |  |  |
|  |  | 5-14 |  |  |  |  |  |
|  |  | 15-29 |  |  |  | 0.97 | 1 |
|  |  | 30-44 |  | 0.94 | 0.96 | 0.98 | 0.97 |
|  |  | 45-59 | 0.91 | 0.94 | 0.96 | 0.97 | 1 |
|  |  | 60-69 | 0.92 | 0.95 | 0.96 | 0.98 |  |
|  |  | 70-79 | 1 | 0.98 | 0.96 | 0.97 |  |
|  |  | 80+ | 1 | 0.98 | 0.96 | 0.97 |  |
| Finland | F | 0-4 |  |  |  |  |  |
|  |  | 5-14 |  |  |  |  |  |
|  |  | 15-29 |  |  |  | 1 | 1 |
|  |  | 30-44 |  | 1 | 1 | 1 | 1 |
|  |  | 45-59 | 1 | 0.95 | 0.97 | 1 | 1 |
|  |  | 60-69 | 1 | 0.93 | 0.95 | 1 |  |
|  |  | 70-79 | 0.97 | 0.99 | 0.97 | 1 |  |
|  |  | 80+ | 0.97 | 0.99 | 0.97 | 1 |  |

Table S6 (continued)

| **WHO Region** |  |  | **Beta** | | | | |
| --- | --- | --- | --- | --- | --- | --- | --- |
| **Country** | **Sex** | **Age** | **TB** | **IHD** | **Stroke** | **Suicide** | **Homi-cide** |
| France | M | 0-4 |  |  |  |  |  |
|  |  | 5-14 |  |  |  |  |  |
|  |  | 15-29 |  |  |  | 0.98 | 0.96 |
|  |  | 30-44 | 0.94 | 0.98 | 0.97 | 0.99 | 0.96 |
|  |  | 45-59 | 0.92 | 0.97 | 0.96 | 0.98 | 0.96 |
|  |  | 60-69 | 0.94 | 0.97 | 0.96 | 0.97 | 1 |
|  |  | 70-79 | 0.97 | 0.98 | 0.95 | 0.97 | 1 |
|  |  | 80+ | 0.97 | 0.98 | 0.95 | 0.97 | 1 |
| France | F | 0-4 |  |  |  |  |  |
|  |  | 5-14 |  |  |  |  |  |
|  |  | 15-29 |  |  |  | 0.97 | 0.96 |
|  |  | 30-44 | 0.91 | 1 | 0.97 | 0.98 | 0.96 |
|  |  | 45-59 | 0.9 | 0.96 | 0.96 | 0.98 | 1 |
|  |  | 60-69 | 0.94 | 0.96 | 0.96 | 0.97 | 1 |
|  |  | 70-79 | 1 | 0.98 | 0.96 | 0.96 | 0.96 |
|  |  | 80+ | 1 | 0.98 | 0.96 | 0.96 | 0.96 |
| Germany | M | 0-4 |  |  |  |  |  |
|  |  | 5-14 |  |  |  |  |  |
|  |  | 15-29 |  |  |  | 0.98 | 0.95 |
|  |  | 30-44 | 0.93 | 0.97 | 0.97 | 0.98 | 0.93 |
|  |  | 45-59 | 0.9 | 0.96 | 0.96 | 0.98 | 0.93 |
|  |  | 60-69 | 0.86 | 0.95 | 0.95 | 0.98 | 0.93 |
|  |  | 70-79 | 0.9 | 0.97 | 0.94 | 0.96 | 0.93 |
|  |  | 80+ | 0.9 | 0.97 | 0.94 | 0.96 | 0.93 |
| Germany | F | 0-4 |  |  |  |  |  |
|  |  | 5-14 |  |  |  |  |  |
|  |  | 15-29 |  |  |  | 0.97 | 0.95 |
|  |  | 30-44 | 0.91 | 1 | 0.96 | 0.97 | 0.95 |
|  |  | 45-59 | 0.91 | 0.96 | 0.96 | 0.96 | 0.97 |
|  |  | 60-69 | 0.9 | 0.95 | 0.94 | 0.96 | 0.98 |
|  |  | 70-79 | 0.93 | 1 | 0.96 | 0.95 | 1 |
|  |  | 80+ | 0.93 | 1 | 0.96 | 0.95 | 1 |
| Greece | M | 0-4 |  |  |  |  |  |
|  |  | 5-14 |  |  |  |  |  |
|  |  | 15-29 |  |  |  | 1 | 1 |
|  |  | 30-44 | 1 | 1 |  | 1 | 1 |
|  |  | 45-59 | 0.93 | 1 | 0.98 | 1 | 1 |
|  |  | 60-69 | 0.9 | 0.98 | 0.98 | 1 |  |
|  |  | 70-79 | 0.91 | 0.98 | 0.98 | 0.96 |  |
|  |  | 80+ | 0.91 | 0.98 | 0.98 | 0.96 |  |
| Greece | F | 0-4 |  |  |  |  |  |
|  |  | 5-14 |  |  |  |  |  |
|  |  | 15-29 |  |  |  | 1 | 1 |
|  |  | 30-44 | 1 | 1 |  | 1 | 1 |
|  |  | 45-59 | 1 | 1 | 0.97 | 0.96 | 1 |
|  |  | 60-69 | 1 | 0.98 | 0.96 | 1 |  |
|  |  | 70-79 | 0.9 | 0.99 | 0.98 | 0.94 |  |
|  |  | 80+ | 0.9 | 0.99 | 0.98 | 0.94 |  |

| **WHO Region** |  |  | **Beta** | | | | |
| --- | --- | --- | --- | --- | --- | --- | --- |
| **Country** | **Sex** | **Age** | **TB** | **IHD** | **Stroke** | **Suicide** | **Homi-cide** |
| Hungary | M | 0-4 |  |  |  |  |  |
|  |  | 5-14 |  |  |  |  |  |
|  |  | 15-29 |  | 0.93 | 0.94 | 0.97 | 0.95 |
|  |  | 30-44 | 0.97 | 0.96 | 0.97 | 0.98 | 1 |
|  |  | 45-59 | 0.95 | 0.98 | 0.97 | 0.98 | 1 |
|  |  | 60-69 | 0.92 | 0.99 | 0.98 | 0.97 | 0.95 |
|  |  | 70-79 | 0.93 | 1.01 | 0.98 | 0.97 | 0.95 |
|  |  | 80+ | 0.93 | 1.01 | 0.98 | 0.97 | 0.95 |
| Hungary | F | 0-4 |  |  |  |  |  |
|  |  | 5-14 |  |  |  |  |  |
|  |  | 15-29 |  |  | 0.95 | 0.94 | 1 |
|  |  | 30-44 | 1 | 0.98 | 0.96 | 0.95 | 1 |
|  |  | 45-59 | 0.94 | 0.98 | 0.97 | 0.96 | 1 |
|  |  | 60-69 | 0.91 | 0.99 | 0.97 | 0.94 | 1 |
|  |  | 70-79 | 0.93 | 1.01 | 0.98 | 0.95 | 1 |
|  |  | 80+ | 0.93 | 1.01 | 0.98 | 0.95 | 1 |
| Israel | M | 0-4 |  |  |  |  |  |
|  |  | 5-14 |  |  |  |  |  |
|  |  | 15-29 |  |  |  | 1.03 |  |
|  |  | 30-44 |  |  |  | 1 |  |
|  |  | 45-59 | 1 | 0.93 | 0.95 | 1 |  |
|  |  | 60-69 | 1 | 0.94 | 0.96 | 1 |  |
|  |  | 70-79 | 1 | 0.97 | 0.96 | 1 |  |
|  |  | 80+ | 1 | 0.97 | 0.96 | 1 |  |
| Israel | F | 0-4 |  |  |  |  |  |
|  |  | 5-14 |  |  |  |  |  |
|  |  | 15-29 |  |  |  | 1 |  |
|  |  | 30-44 |  |  |  | 1 |  |
|  |  | 45-59 | 1 | 0.9 | 0.92 | 0.94 |  |
|  |  | 60-69 | 1 | 0.93 | 0.94 | 1 |  |
|  |  | 70-79 | 1 | 0.97 | 0.95 | 0.95 |  |
|  |  | 80+ | 1 | 0.97 | 0.95 | 0.95 |  |
| Italy | M | 0-4 |  |  |  |  |  |
|  |  | 5-14 |  |  |  |  |  |
|  |  | 15-29 |  | 0.97 |  | 1.01 | 0.95 |
|  |  | 30-44 | 0.93 | 0.95 | 0.96 | 1 | 0.92 |
|  |  | 45-59 | 0.89 | 0.96 | 0.95 | 0.99 | 0.93 |
|  |  | 60-69 | 0.9 | 0.96 | 0.95 | 0.98 | 0.95 |
|  |  | 70-79 | 0.94 | 0.99 | 0.96 | 0.97 | 0.96 |
|  |  | 80+ | 0.94 | 0.99 | 0.96 | 0.97 | 0.96 |
| Italy | F | 0-4 |  |  |  |  |  |
|  |  | 5-14 |  |  |  |  |  |
|  |  | 15-29 |  |  |  | 1 | 1 |
|  |  | 30-44 | 1 | 0.96 | 0.97 | 1 | 1 |
|  |  | 45-59 | 0.93 | 0.96 | 0.95 | 0.97 | 1 |
|  |  | 60-69 | 0.92 | 0.96 | 0.94 | 0.97 | 1 |
|  |  | 70-79 | 0.96 | 0.99 | 0.97 | 0.96 | 0.95 |
|  |  | 80+ | 0.96 | 0.99 | 0.97 | 0.96 | 0.95 |

Table S6 (continued)

| **WHO Region** |  |  | **Beta** | | | | |
| --- | --- | --- | --- | --- | --- | --- | --- |
| **Country** | **Sex** | **Age** | **TB** | **IHD** | **Stroke** | **Suicide** | **Homi-cide** |
| Kazakhstan | M | 0-4 | 1.11 |  | 1.12 |  | 1.1 |
|  |  | 5-14 | 1.09 |  |  |  | 1.05 |
|  |  | 15-29 | 1.07 |  |  | 1.07 | 1.02 |
|  |  | 30-44 | 1.06 | 1.03 | 1.04 | 1.07 | 1.03 |
|  |  | 45-59 | 1.05 | 1.02 | 1.02 | 1.07 | 1.06 |
|  |  | 60-69 | 1.02 | 1.03 | 1.02 | 1.08 | 1.07 |
|  |  | 70-79 | 1 | 1.01 | 1 | 1.04 | 1.08 |
|  |  | 80+ | 1 | 1.01 | 1 | 1.04 | 1.08 |
| Kazakhstan | F | 0-4 | 1.09 |  | 1.19 |  | 1.1 |
|  |  | 5-14 | 1.09 |  |  |  | 1.1 |
|  |  | 15-29 | 1.09 |  |  | 1.05 | 1.05 |
|  |  | 30-44 | 1.1 | 1.05 | 1.03 | 1.06 | 1.05 |
|  |  | 45-59 | 1.06 | 1.02 | 1.01 | 1 | 1.07 |
|  |  | 60-69 | 1.03 | 1.03 | 1.02 | 1.02 | 1.11 |
|  |  | 70-79 | 1 | 1.02 | 1.01 | 1 | 1.1 |
|  |  | 80+ | 1 | 1.02 | 1.01 | 1 | 1.1 |
| Netherlands | M | 0-4 |  |  |  |  |  |
|  |  | 5-14 |  |  |  |  |  |
|  |  | 15-29 |  |  |  | 1 | 1 |
|  |  | 30-44 |  | 0.97 | 0.97 | 1 | 1 |
|  |  | 45-59 | 1 | 0.95 | 0.98 | 1 | 1 |
|  |  | 60-69 | 0.89 | 0.95 | 0.98 | 0.98 |  |
|  |  | 70-79 | 0.96 | 0.97 | 0.98 | 0.97 |  |
|  |  | 80+ | 0.96 | 0.97 | 0.98 | 0.97 |  |
| Netherlands | F | 0-4 |  |  |  |  |  |
|  |  | 5-14 |  |  |  |  |  |
|  |  | 15-29 |  |  |  | 1 | 1.06 |
|  |  | 30-44 |  | 1 | 1 | 0.98 | 1 |
|  |  | 45-59 | 1 | 0.96 | 1 | 1 | 1 |
|  |  | 60-69 | 1 | 0.97 | 0.99 | 0.97 |  |
|  |  | 70-79 | 0.96 | 0.98 | 0.99 | 0.97 |  |
|  |  | 80+ | 0.96 | 0.98 | 0.99 | 0.97 |  |
| Poland | M | 0-4 |  |  |  |  |  |
|  |  | 5-14 |  |  |  |  |  |
|  |  | 15-29 |  |  | 0.98 | 1.01 | 0.98 |
|  |  | 30-44 | 0.98 | 0.95 | 0.99 | 1 | 0.97 |
|  |  | 45-59 | 0.95 | 0.98 | 0.99 | 1.01 | 0.98 |
|  |  | 60-69 | 0.94 | 1.02 | 1.02 | 1.01 |  |
|  |  | 70-79 | 0.93 | 1.08 | 1.03 | 1 |  |
|  |  | 80+ | 0.93 | 1.08 | 1.03 | 1 |  |
| Poland | F | 0-4 |  |  |  |  |  |
|  |  | 5-14 |  |  |  |  |  |
|  |  | 15-29 |  |  | 0.96 | 1 | 1 |
|  |  | 30-44 | 0.96 | 0.95 | 0.99 | 0.99 | 0.96 |
|  |  | 45-59 | 0.94 | 0.98 | 0.99 | 1.01 | 0.97 |
|  |  | 60-69 | 0.92 | 1.02 | 1 | 1 | 0.97 |
|  |  | 70-79 | 0.95 | 1.1 | 1.03 | 1 | 0.94 |
|  |  | 80+ | 0.95 | 1.1 | 1.03 | 1 | 0.94 |

| **WHO Region** |  |  | **Beta** | | | | |
| --- | --- | --- | --- | --- | --- | --- | --- |
| **Country** | **Sex** | **Age** | **TB** | **IHD** | **Stroke** | **Suicide** | **Homi-cide** |
| Portugal | M | 0-4 |  |  |  |  |  |
|  |  | 5-14 |  |  |  |  |  |
|  |  | 15-29 |  |  |  | 0.98 | 1 |
|  |  | 30-44 |  | 1 |  | 1 | 1 |
|  |  | 45-59 | 0.95 | 0.98 | 0.96 | 0.97 | 0.96 |
|  |  | 60-69 | 0.94 | 0.98 | 0.96 | 1 | 0.95 |
|  |  | 70-79 | 0.98 | 1 | 0.96 | 1 |  |
|  |  | 80+ | 0.98 | 1 | 0.96 | 1 |  |
| Portugal | F | 0-4 |  |  |  |  |  |
|  |  | 5-14 |  |  |  |  |  |
|  |  | 15-29 |  |  |  | 0.93 | 1 |
|  |  | 30-44 |  | 0.97 |  | 0.97 | 1 |
|  |  | 45-59 | 1 | 0.98 | 0.95 | 0.97 | 1 |
|  |  | 60-69 | 0.95 | 0.97 | 0.95 | 1 | 1 |
|  |  | 70-79 | 1 | 1 | 0.96 | 1 |  |
|  |  | 80+ | 1 | 1 | 0.96 | 1 |  |
| Romania | M | 0-4 |  |  |  |  |  |
|  |  | 5-14 |  |  |  |  |  |
|  |  | 15-29 |  |  |  | 1.01 | 0.95 |
|  |  | 30-44 |  | 0.99 |  | 1.02 | 0.97 |
|  |  | 45-59 | 1.02 | 1.01 | 1 | 1.03 | 0.97 |
|  |  | 60-69 | 1.05 | 1.02 | 1.01 | 1.02 |  |
|  |  | 70-79 | 1.07 | 1.02 | 1 | 1 |  |
|  |  | 80+ | 1.07 | 1.02 | 1 | 1 |  |
| Romania | F | 0-4 |  |  |  |  |  |
|  |  | 5-14 |  |  |  |  |  |
|  |  | 15-29 |  |  |  | 0.96 | 1 |
|  |  | 30-44 | 1.02 | 1 |  | 0.97 | 0.96 |
|  |  | 45-59 | 1.04 | 1.01 | 0.99 | 1 | 1 |
|  |  | 60-69 | 1.06 | 1.01 | 1 | 1 |  |
|  |  | 70-79 | 1.09 | 1.01 | 1 | 1 |  |
|  |  | 80+ | 1.09 | 1.01 | 1 | 1 |  |
| Russian Federation | M | 0-4 | 1.09 |  | 1.11 |  | 1.13 |
|  |  | 5-14 | 1.09 |  | 1.06 |  | 1.05 |
|  |  | 15-29 | 1.1 | 1.03 | 1.03 | 1.03 | 1.01 |
|  |  | 30-44 | 1.08 | 1.02 | 1.05 | 1.01 | 1.02 |
|  |  | 45-59 | 1.07 | 1.01 | 1.01 | 1 | 1.05 |
|  |  | 60-69 | 1.03 | 1.03 | 1.03 | 1 | 1.05 |
|  |  | 70-79 | 0.98 | 1 | 1 | 0.99 | 1.04 |
|  |  | 80+ | 0.98 | 1 | 1 | 0.99 | 1.04 |
| Russian Federation | F | 0-4 | 1.11 |  | 1.09 |  | 1.12 |
|  |  | 5-14 | 1.12 |  | 1.05 |  | 1.05 |
|  |  | 15-29 | 1.12 | 1.04 | 1.01 | 1.02 | 1.04 |
|  |  | 30-44 | 1.13 | 1.04 | 1.04 | 1 | 1.02 |
|  |  | 45-59 | 1.1 | 1.01 | 1.01 | 0.97 | 1.03 |
|  |  | 60-69 | 1.02 | 1.01 | 1.01 | 0.97 | 1.04 |
|  |  | 70-79 | 0.96 | 1 | 1.01 | 0.98 | 1.03 |
|  |  | 80+ | 0.96 | 1 | 1.01 | 0.98 | 1.03 |

Table S6 (continued)

| **WHO Region** |  |  | **Beta** | | | | |
| --- | --- | --- | --- | --- | --- | --- | --- |
| **Country** | **Sex** | **Age** | **TB** | **IHD** | **Stroke** | **Suicide** | **Homi-cide** |
| Slovakia | M | 0-4 |  |  |  |  |  |
|  |  | 5-14 |  |  |  |  |  |
|  |  | 15-29 |  |  |  |  | 1 |
|  |  | 30-44 |  | 0.94 | 0.95 |  | 1 |
|  |  | 45-59 | 1 | 0.95 | 0.94 |  | 1 |
|  |  | 60-69 | 1 | 0.98 | 0.97 |  |  |
|  |  | 70-79 | 0.85 | 1.01 | 0.99 |  |  |
|  |  | 80+ | 0.85 | 1.01 | 0.99 |  |  |
| Slovakia | F | 0-4 |  |  |  |  |  |
|  |  | 5-14 |  |  |  |  |  |
|  |  | 15-29 |  |  |  |  | 1 |
|  |  | 30-44 |  | 1 | 0.91 |  | 1 |
|  |  | 45-59 | 1 | 0.94 | 0.92 |  | 1 |
|  |  | 60-69 | 1 | 0.97 | 0.94 |  |  |
|  |  | 70-79 | 0.91 | 1.02 | 0.98 |  |  |
|  |  | 80+ | 0.91 | 1.02 | 0.98 |  |  |
| Spain | M | 0-4 |  |  |  |  |  |
|  |  | 5-14 |  |  |  |  |  |
|  |  | 15-29 | 0.89 |  |  |  | 1 |
|  |  | 30-44 | 0.89 | 0.98 | 0.97 | 1.02 | 1 |
|  |  | 45-59 | 0.91 | 0.99 | 0.96 | 0.99 | 1 |
|  |  | 60-69 | 0.91 | 0.99 | 0.96 | 1 |  |
|  |  | 70-79 | 0.94 | 1.01 | 0.96 | 0.99 |  |
|  |  | 80+ | 0.94 | 1.01 | 0.96 | 0.99 |  |
| Spain | F | 0-4 |  |  |  |  |  |
|  |  | 5-14 |  |  |  |  |  |
|  |  | 15-29 | 0.92 |  |  |  | 1 |
|  |  | 30-44 | 0.93 | 1 | 0.98 | 1.02 | 1 |
|  |  | 45-59 | 0.94 | 0.99 | 0.96 | 1 | 1 |
|  |  | 60-69 | 0.93 | 0.99 | 0.95 | 0.98 |  |
|  |  | 70-79 | 0.96 | 1.02 | 0.96 | 0.98 |  |
|  |  | 80+ | 0.96 | 1.02 | 0.96 | 0.98 |  |
| Sweden | M | 0-4 |  |  |  |  |  |
|  |  | 5-14 |  |  |  |  |  |
|  |  | 15-29 |  |  |  | 0.97 | 1 |
|  |  | 30-44 |  | 0.95 | 0.96 | 0.97 | 1 |
|  |  | 45-59 | 1 | 0.95 | 0.98 | 0.97 | 1 |
|  |  | 60-69 | 1 | 0.95 | 0.98 | 0.98 |  |
|  |  | 70-79 | 0.96 | 0.97 | 1 | 0.98 |  |
|  |  | 80+ | 0.96 | 0.97 | 1 | 0.98 |  |
| Sweden | F | 0-4 |  |  |  |  |  |
|  |  | 5-14 |  |  |  |  |  |
|  |  | 15-29 |  |  |  | 0.97 | 1 |
|  |  | 30-44 |  | 0.95 | 0.96 | 0.95 | 1 |
|  |  | 45-59 | 1 | 0.98 | 0.98 | 0.98 | 1 |
|  |  | 60-69 | 1 | 0.95 | 0.98 | 0.97 |  |
|  |  | 70-79 | 1 | 0.98 | 1 | 0.98 |  |
|  |  | 80+ | 1 | 0.98 | 1 | 0.98 |  |

| **WHO Region** |  |  | **Beta** | | | | |
| --- | --- | --- | --- | --- | --- | --- | --- |
| **Country** | **Sex** | **Age** | **TB** | **IHD** | **Stroke** | **Suicide** | **Homi-cide** |
| Switzerland | M | 0-4 |  |  |  |  |  |
|  |  | 5-14 |  |  |  |  |  |
|  |  | 15-29 |  |  |  | 0.96 | 1 |
|  |  | 30-44 |  | 0.96 | 0.97 | 0.97 | 1 |
|  |  | 45-59 | 1 | 0.96 | 0.97 | 0.99 | 1 |
|  |  | 60-69 | 1 | 0.95 | 0.96 | 0.98 |  |
|  |  | 70-79 | 0.93 | 1 | 0.97 | 1 |  |
|  |  | 80+ | 0.93 | 1 | 0.97 | 1 |  |
| Switzerland | F | 0-4 |  |  |  |  |  |
|  |  | 5-14 |  |  |  |  |  |
|  |  | 15-29 |  |  |  | 0.97 | 1 |
|  |  | 30-44 |  | 1 | 0.95 | 0.96 | 1 |
|  |  | 45-59 | 1 | 0.98 | 0.97 | 0.98 | 1 |
|  |  | 60-69 | 1 | 0.97 | 0.97 | 0.98 |  |
|  |  | 70-79 | 0.95 | 1.01 | 0.97 | 1 |  |
|  |  | 80+ | 0.95 | 1.01 | 0.97 | 1 |  |
| Ukraine | M | 0-4 |  |  |  |  | 1.03 |
|  |  | 5-14 |  |  |  |  | 1 |
|  |  | 15-29 | 1.15 | 1.01 |  | 1.03 | 1.01 |
|  |  | 30-44 | 1.1 | 1.02 | 1.02 | 1.02 | 1.02 |
|  |  | 45-59 | 1.07 | 1.02 | 1 | 1.02 | 1.05 |
|  |  | 60-69 | 1.02 | 1.04 | 1 | 1.02 | 1.05 |
|  |  | 70-79 | 0.98 | 1.01 | 0.98 | 1.02 | 1.04 |
|  |  | 80+ | 0.98 | 1.01 | 0.98 | 1.02 | 1.04 |
| Ukraine | F | 0-4 |  |  |  |  | 1.05 |
|  |  | 5-14 |  |  |  |  | 1.07 |
|  |  | 15-29 | 1.13 | 1.03 |  | 1 | 1.03 |
|  |  | 30-44 | 1.13 | 1.03 | 1.01 | 1.02 | 1.02 |
|  |  | 45-59 | 1.07 | 1.02 | 0.99 | 1 | 1.04 |
|  |  | 60-69 | 1 | 1.02 | 0.99 | 0.99 | 1.05 |
|  |  | 70-79 | 0.95 | 1.01 | 0.98 | 0.99 | 1.05 |
|  |  | 80+ | 0.95 | 1.01 | 0.98 | 0.99 | 1.05 |
| UK | M | 0-4 |  |  |  |  |  |
|  |  | 5-14 |  |  |  |  |  |
|  |  | 15-29 |  |  |  | 0.99 | 1 |
|  |  | 30-44 |  | 0.96 | 0.98 | 1 | 1 |
|  |  | 45-59 | 0.97 | 0.94 | 0.97 | 0.99 | 1 |
|  |  | 60-69 | 0.92 | 0.95 | 0.96 | 0.97 |  |
|  |  | 70-79 | 0.96 | 0.97 | 0.98 | 0.96 |  |
|  |  | 80+ | 0.96 | 0.97 | 0.98 | 0.96 |  |
| UK | F | 0-4 |  |  |  |  |  |
|  | F | 5-14 |  |  |  |  |  |
|  | F | 15-29 |  |  |  | 1 | 0.97 |
|  | F | 30-44 |  | 0.97 | 0.98 | 1 | 1 |
|  | F | 45-59 | 0.94 | 0.94 | 0.98 | 0.97 | 1 |
|  | F | 60-69 | 0.94 | 0.94 | 0.96 | 0.94 |  |
|  | F | 70-79 | 0.98 | 0.97 | 0.99 | 0.95 |  |
|  | F | 80+ | 0.98 | 0.97 | 0.99 | 0.95 |  |

Table S6 (continued)

| **WHO Region** |  |  | **Beta** | | | | |
| --- | --- | --- | --- | --- | --- | --- | --- |
| **Country** | **Sex** | **Age** | **TB** | **IHD** | **Stroke** | **Suicide** | **Homi-cide** |
| Uzbekistan | M | 0-4 |  |  |  |  |  |
|  |  | 5-14 |  |  |  |  |  |
|  |  | 15-29 | 1.15 |  | 0.91 | 1.04 | 0.96 |
|  |  | 30-44 | 1.11 | 0.98 | 0.95 | 1.01 | 0.95 |
|  |  | 45-59 | 1.05 | 1 | 0.99 | 1 | 0.97 |
|  |  | 60-69 | 1.03 | 1.03 | 1 | 1 |  |
|  |  | 70-79 | 1 | 1 | 0.99 | 1 |  |
|  |  | 80+ | 1 | 1 | 0.99 | 1 |  |
| Uzbekistan | F | 0-4 |  |  |  |  |  |
|  |  | 5-14 |  |  |  |  |  |
|  |  | 15-29 | 1.09 |  | 0.9 | 1.03 | 1 |
|  |  | 30-44 | 1.07 | 1 | 0.93 | 1 | 1 |
|  |  | 45-59 | 1 | 1 | 0.98 | 0.96 | 1 |
|  |  | 60-69 | 1 | 1.03 | 1.01 | 0.96 |  |
|  |  | 70-79 | 1 | 1.02 | 1.02 | 0.95 |  |
|  |  | 80+ | 1 | 1.02 | 1.02 | 0.95 |  |
| *WPRO Region* | | | | | | | |
| Australia | M | 0-4 |  |  |  |  |  |
|  |  | 5-14 |  |  |  |  |  |
|  |  | 15-29 |  |  |  | 1 | 1 |
|  |  | 30-44 |  | 0.98 | 0.97 | 1.02 | 1 |
|  |  | 45-59 |  | 0.94 | 0.95 | 1 | 1 |
|  |  | 60-69 | 0.93 | 0.94 | 0.95 | 0.98 |  |
|  |  | 70-79 | 0.95 | 0.96 | 0.97 | 0.98 |  |
|  |  | 80+ | 0.95 | 0.96 | 0.97 | 0.98 |  |
| Australia | F | 0-4 |  |  |  |  |  |
|  |  | 5-14 |  |  |  |  |  |
|  |  | 15-29 |  |  |  | 1 | 0.96 |
|  |  | 30-44 |  | 1 | 0.97 | 1 | 1 |
|  |  | 45-59 |  | 0.93 | 0.95 | 1 | 1 |
|  |  | 60-69 | 1 | 0.93 | 0.94 | 0.96 |  |
|  |  | 70-79 | 1 | 0.97 | 0.98 | 0.97 |  |
|  |  | 80+ | 1 | 0.97 | 0.98 | 0.97 |  |

| **WHO Region** |  |  | **Beta** | | | | |
| --- | --- | --- | --- | --- | --- | --- | --- |
| **Country** | **Sex** | **Age** | **TB** | **IHD** | **Stroke** | **Suicide** | **Homi-cide** |
| Japan | M | 0-4 |  |  |  |  | 0.97 |
|  |  | 5-14 |  |  |  |  | 0.96 |
|  |  | 15-29 |  | 1.08 |  | 1.04 | 0.96 |
|  |  | 30-44 | 0.94 | 1.05 | 0.97 | 1.03 | 0.96 |
|  |  | 45-59 | 0.94 | 1.06 | 0.97 | 1.05 | 1 |
|  |  | 60-69 | 0.93 | 1.04 | 0.98 | 1.05 | 1.03 |
|  |  | 70-79 | 0.97 | 1.01 | 0.97 | 0.99 | 1 |
|  |  | 80+ | 0.97 | 1.01 | 0.97 | 0.99 | 1 |
| Japan | F | 0-4 |  |  |  |  | 0.95 |
|  |  | 5-14 |  |  |  |  | 0.96 |
|  |  | 15-29 |  |  |  | 1.02 | 0.96 |
|  |  | 30-44 | 0.9 | 1.04 | 0.97 | 1.01 | 0.96 |
|  |  | 45-59 | 0.89 | 1.05 | 0.97 | 1 | 1 |
|  |  | 60-69 | 0.94 | 1.03 | 0.96 | 1 | 1 |
|  |  | 70-79 | 1 | 1.01 | 0.97 | 0.96 | 1 |
|  |  | 80+ | 1 | 1.01 | 0.97 | 0.96 | 1 |
| Republic of Korea | M | 0-4 | 0.81 |  | 0.9 |  | 1 |
|  |  | 5-14 | 0.74 |  | 0.86 |  | 1 |
|  |  | 15-29 | 0.84 | 0.96 | 0.9 | 0.97 | 0.93 |
|  |  | 30-44 | 0.89 | 1 | 0.94 | 1.02 | 0.98 |
|  |  | 45-59 | 0.89 | 1.05 | 0.93 | 1.04 | 1 |
|  |  | 60-69 | 0.9 | 1.05 | 0.93 | 1.05 | 1 |
|  |  | 70-79 | 0.96 | 1.05 | 0.95 | 1.07 | 1 |
|  |  | 80+ | 0.96 | 1.05 | 0.95 | 1.07 | 1 |
| Republic of Korea | F | 0-4 | 0.82 |  | 0.91 |  | 1 |
|  |  | 5-14 | 0.77 |  | 0.87 |  | 1 |
|  |  | 15-29 | 0.84 | 0.95 | 0.91 | 0.99 | 1 |
|  |  | 30-44 | 0.85 | 1 | 0.92 | 1.01 | 1.03 |
|  |  | 45-59 | 0.87 | 1.03 | 0.91 | 1.03 | 1.03 |
|  |  | 60-69 | 0.89 | 1.04 | 0.93 | 1.04 | 1 |
|  |  | 70-79 | 0.97 | 1.05 | 0.96 | 1.08 | 1 |
|  |  | 80+ | 0.97 | 1.05 | 0.96 | 1.08 | 1 |
