# Supplementary material for: Projections of Global Mortality and Burden of Disease from 2002 to 2030
Source: PLoS Med. 2006 Nov 28;3(11):e442. doi: 10.1371/journal.pmed.0030442 (PMC1664601; doi:10.1371/journal.pmed.0030442)
Supplement: Table S7 — (67 KB DOC) [file pmed.0030442.st007.doc]

Table S7: Summary of assumptions and inputs for baseline, optimistic and pessimistic projection scenarios

|  | **Baseline scenario** | **Pessimistic scenario** | **Optimistic scenario** |
| --- | --- | --- | --- |
| **Projected covariates** |  |  |  |
| GDP per capita | World Bank projections | Assume GDP annual growth rates from 2005 at 50% of baseline projections, with some regional variations | Assume GDP annual growth rates from 2005 approximately 40% higher than baseline projections, somewhat lower increase for high income countries, China and India |
| Human capital | EIP projections based on projected GDP growth | Approximately 1% lowerannual growth with GDP compared to baseline scenario | Approximately 1% higher annual growth with GDP compared to baseline scenario |
| Smoking intensity | Weighted average based on previous EIP regional projections, country trends in apparent consumption, and lung cancer trends where available. | Weighted average giving more weight to previous EIP regional projections. | Weighted average giving more weight to country trends in apparent consumption. |
| **Adjustment factor for major cause regression coefficients** | | | |
| Time | 0 for AFRO low income countries, 0.25 for other low income countries | 0 for AFRO low income countries, 0 for other low income countries | 0.25 for AFRO low income countries, 0.75 for other low income countries |
| Human capital | 0.5 for low income countries | 0.25 for low income countries | No adjustment |
| **Disease-specific mortality projections** | | | |
| HIV/AIDS | Achievement of 80% ARV coverage by 2012 | Achievement of 60% ARV coverage by 2012 in all regions except Latin America (60% by 2013) | Achievement of 80% ARV coverage by 2012 plus additional prevention activities |
| Tuberculosis | Sustained DOTS scenario: case detection and treatment success rates increase until 2005 and then remain constant to 2030 | Annual trends halfway between sustained DOTS scenario and “No DOTS” scenario from 2006 onwards. “No DOTS” scenario assumes pre-DOTS case detection and cure rates. | Annual trends projected with full implementation of the GLobal Plan to Stop TB 2006-2015. Annual trends from 2015 to 2030 remain constant. |
| Diabetes | Non-overweight death rates declining at 75% of rate for Other Group II deaths | Non-overweight death rates declining at 50% of rate for Other Group II deaths | Non-overweight death rates declining at 100% of rate for Other Group II deaths |
| COPD and asthma | Non-smoker death rates declining at 75% of rate for Other Group II deaths | Non-smoker death rates declining at 50% of rate for Other Group II deaths | Non-smoker death rates declining at 100% of rate for Other Group II deaths |
| Violence | Assume rates constant over time in high income countries | Projected trends for intentional injuries assumed to apply | Assume rates constant over time in high income countries |
| War | Assume rates constant over time in all regions | Assume rates rise for EMRO and AMRO regions between 2002 and 2005 and then remain constant over time in all regions | Assume rates decline at 1.5% per annum from 2006 onwards. |
